# NR5A2 transcriptional activation by BRD4 promotes pancreatic cancer progression by upregulating GDF15

**DOI:** 10.1038/s41420-021-00462-8

**Published:** 2021-04-13

**Authors:** Feng Guo, Yingke Zhou, Hui Guo, Dianyun Ren, Xin Jin, Heshui Wu

**Affiliations:** 1 Department of Pancreatic Surgery, Union Hospital, Tongji Medical College, Huazhong University of Science and Technology, Wuhan, 430022 China; 2 Sino-German Laboratory of Personalized Medicine for Pancreatic Cancer, Union Hospital, Tongji Medical College, Huazhong University of Science and Technology, Wuhan, 430022 China; 3 Department of Breast and Thyroid Surgery, Union Hospital, Tongji Medical College, Huazhong University of Science and Technology, Wuhan, China; 4 Department of Urology, The Second Xiangya Hospital, Central South University, Changsha, Hunan, 410011 China

**Keywords:** Targeted therapies, Cancer

## Abstract

NR5A2 is a transcription factor regulating the expression of various oncogenes. However, the role of NR5A2 and the specific regulatory mechanism of NR5A2 in pancreatic ductal adenocarcinoma (PDAC) are not thoroughly studied. In our study, Western blotting, real-time PCR, and immunohistochemistry were conducted to assess the expression levels of different molecules. Wound-healing, MTS, colony formation, and transwell assays were employed to evaluate the malignant potential of pancreatic cancer cells. We demonstrated that NR5A2 acted as a negative prognostic biomarker in PDAC. NR5A2 silencing inhibited the proliferation and migration abilities of pancreatic cancer cells in vitro and in vivo. While NR5A2 overexpression markedly promoted both events in vitro. We further identified that NR5A2 was transcriptionally upregulated by BRD4 in pancreatic cancer cells and this was confirmed by Chromatin immunoprecipitation (ChIP) and ChIP-qPCR. Besides, transcriptome RNA sequencing (RNA-Seq) was performed to explore the cancer-promoting effects of NR5A2, we found that GDF15 is a component of multiple down-regulated tumor-promoting gene sets after NR5A2 was silenced. Next, we showed that NR5A2 enhanced the malignancy of pancreatic cancer cells by inducing the transcription of GDF15. Collectively, our findings suggest that NR5A2 expression is induced by BRD4. In turn, NR5A2 activates the transcription of GDF15, promoting pancreatic cancer progression. Therefore, NR5A2 and GDF15 could be promising therapeutic targets in pancreatic cancer.

## Introduction

Pancreatic cancer is the seventh leading cause of cancer-related deaths worldwide^1^, making it one of the most lethal types of cancer^2^. According to the GLOBOCAN estimates, in 2018, there were 458,918 pancreatic cancer cases and 432,242 pancreatic cancer-related deaths worldwide^1^. Because most patients do not exhibit symptoms until they are at an advanced stage of the disease, only ~20% of patients newly diagnosed with pancreatic cancer benefit from a potentially curative surgical resection; the remaining 80% are diagnosed with unresectable locally advanced pancreatic cancer or metastatic pancreatic cancer^3^. As most patients with pancreatic cancer do not benefit from radiotherapy or chemotherapy, surgery remains the only curative treatment^4^. Therefore, elucidating the pathogenesis of pancreatic cancer and identifying potential therapeutic targets is paramount to improving survival outcomes in patients with pancreatic cancer.

The nuclear receptor subfamily 5 group A member 2 (NR5A2), also known as liver receptor homolog-1 (LRH-1), is a member of the orphan nuclear hormone receptors. NR5A2 is highly expressed in the pancreas, liver, intestine, and ovaries and has been shown to regulate steroidogenesis and the levels of cholesterol and bile acids^5,6^. According to Luo et al.^7^, NR5A2 is overexpressed in pancreatic cancer and promotes epithelial-to-mesenchymal transition. It is also been reported that NR5A2 overexpression in pancreatic cancer cell lines promoted cell migration, wound healing, cell invasion, and sphere formation^8^. In general, NR5A2 functions as a transcription factor that drives pancreatic cancer progression by activating or inhibiting the transcription of oncogenes and tumor suppressor genes^9^. Interestingly, NR5A2 polymorphisms have been reported to predict a good prognosis in patients with pancreatic cancer, suggesting that NR5A2 may also act as a tumor suppressor^10^. In addition, NR5A2 can suppress oncogenic KRAS-driven pancreatic neoplasia^11,12^. Yet, the precise role of NR5A2 in pancreatic cancer progression remains unclear.

In this study, we found that NR5A2 is a negative prognostic factor in pancreatic cancer and promotes proliferation and migration of pancreatic cancer cells, acting as a negative prognostic factor in pancreatic cancer. NR5A2 overexpression induced by BRD4 enhanced the transcription of GDF15 in pancreatic cancer, suggesting that BRD4/NR5A2/GDF15 axis is a potential therapeutic target in pancreatic cancer.

## Results

### NR5A2 overexpression is correlated with unfavorable prognosis in patients with pancreatic cancer

**Fig. 1:**
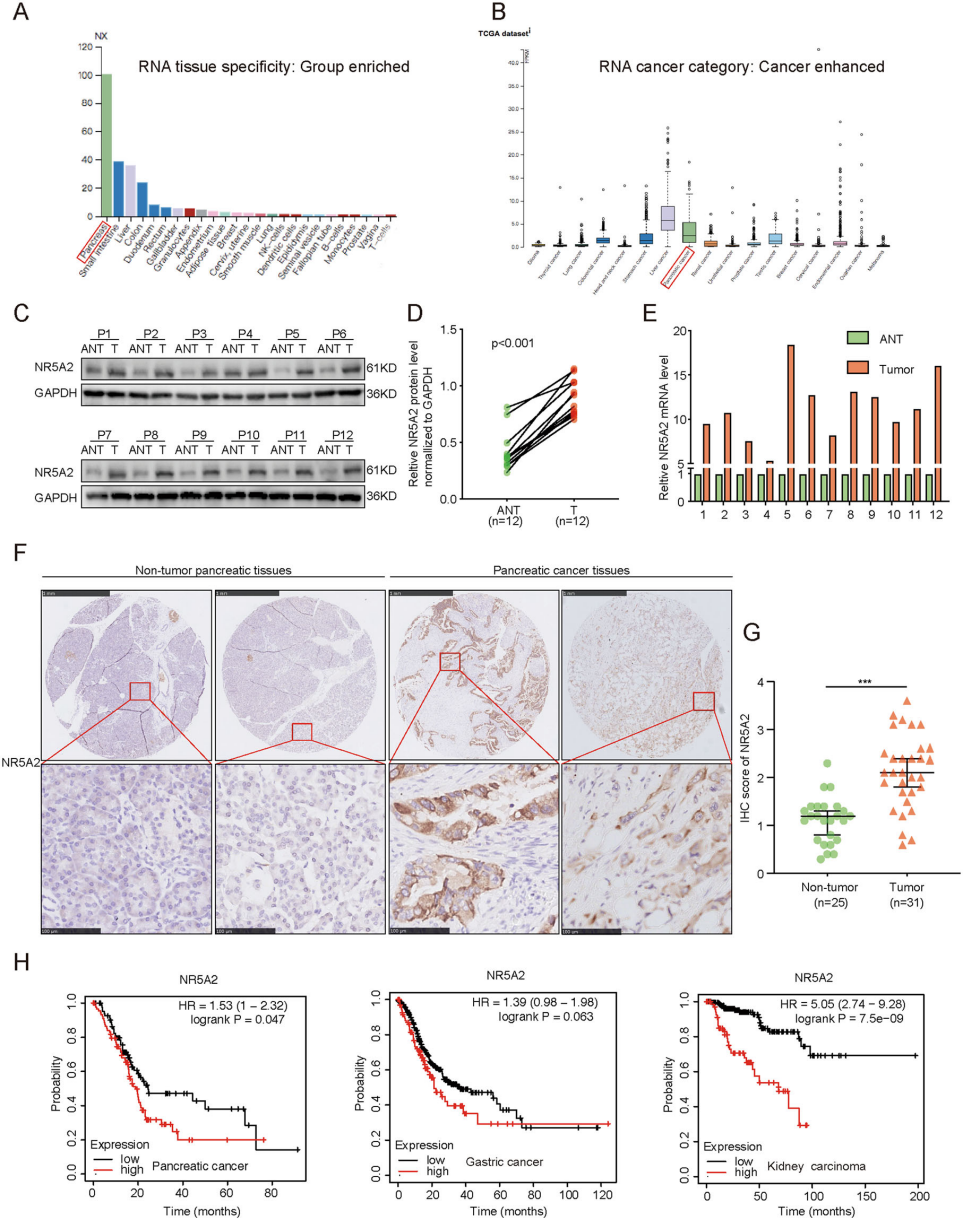
The overexpression of NR5A2 is correlated with unfavorable prognosis in malignant tumors. **A** HPA database was searched for NR5A2 mRNA expression in different normal tissues. **B** HPA database was searched for NR5A2 mRNA expression in different tumor tissues. **C**, **D**. Western blot analysis for the expression of NR5A2 in 12 paired primary PDAC tissues (T) and the matched adjacent normal tissues (ANT) of the same patient (**C**). The protein expression level of NR5A2 were quantified by ImageJ software (**D**). GAPDH served as an internal reference. *p* < 0.001, paired *t* test. **E** RT-qPCR analysis for the expression of NR5A2 in 12 paired primary PDAC tissues (T) and the matched adjacent normal tissues (ANT) of the same patient. GAPDH served as an internal reference. **F**, **G** IHC Images (**F**) and dot plots (**G**) of NR5A2 staining using TMA tissue sections (normal pancreatic specimens: *n* = 25, PDAC TMA specimens: *n* = 31, *P* < 0.001, unpaired *t* test). The scale bars were shown in the figure. **H** K–M plotter was used to analyze the effect of NR5A2 on the overall survival of pancreatic cancer, gastric cancer, and kidney carcinoma.

Analysis of *NR5A2* mRNA expression in different tissues using the HPA database revealed that *NR5A2* is highly expressed in pancreatic tissues compared to other tissues in mRNA level (Fig. 1A). In addition, analysis of TCGA data indicated that the mRNA level of *NR5A2* was relatively high in pancreatic cancer in compare with the most majority of other cancer, except for liver cancer (Fig. 1B). Western blot and real-time quantitative polymerase chain reaction (RT-qPCR) analysis indicated that NR5A2 levels were higher in pancreatic cancer tissues than in paired nonmalignant pancreatic tissues, both at the mRNA and protein levels (Fig. 1C–E). This phenomenon was further confirmed by immunohistochemical (IHC) analysis on a tissue microarray containing normal and tumor tissues (Fig. 1F, G). Notably, *NR5A2* mRNA level was negatively correlated with the survival of patients with pancreatic cancer and other cancer types (gastric cancer and kidney carcinoma) (Fig. 1H). These results suggest that NR5A2 is overexpressed in pancreatic cancer and acts as a negative prognostic factor in pancreatic cancer.

### NR5A2 silencing reduces the malignant potentials of pancreatic cancer cells

**Fig. 2:**
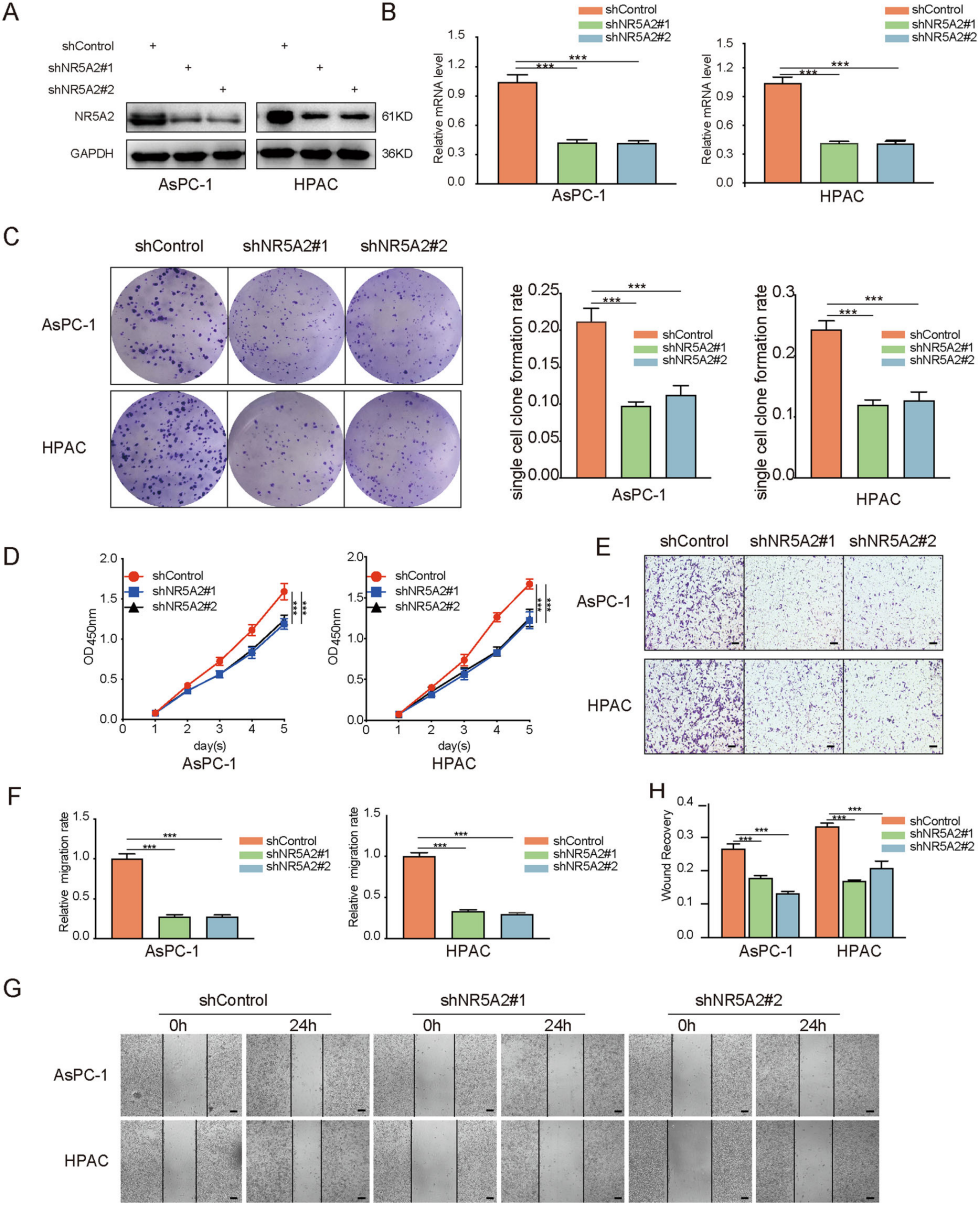
Silencing NR5A2 suppressed potential malignancy of pancreatic cancer cells. **A**, **B** Western blot analysis (**A**) and RT-qPCR (**B**) to show the NR5A2 expression in AsPC-1 and HPAC cells infected with lentivirus vectors expressing control or NR5A2 specific shRNAs. GAPDH served as an internal reference. Data presented as the mean ± SD of three independent experiments. Statistical significance was determined using one-way ANOVA. **C**–**H** AsPC-1 and HPAC cells infected with lentivirus vectors expressing control or NR5A2 specific shRNAs were harvested for colony formation assay (**C**), MTS assay (**D**, *n* = 5), transwell invasion assay (**E**, **F**), and wound-healing assay (**G**, **H**), scale bars: 100 μm. Each bar represents the mean ± SD of three independent experiments. ***P < 0.001, one-way ANOVA.

To elucidate the biological role of NR5A2 in pancreatic cancer cells, we generated NR5A2 knockdown cell lines with two independent shRNAs in AsPC-1 and HPAC cells (Fig. 2A, B). Colony formation and MTS assays indicated that *NR5A2* silencing inhibited the proliferation rate of pancreatic cancer cells (Fig. 2C, D). In addition, transwell invasion (Fig. 2E, F) and wound-healing assays (Fig. 2G, H) revealed that the invasion and migration abilities of pancreatic cancer cells were impaired upon *NR5A2* silencing. These findings suggest that NR5A2 silencing may reduce the malignant potentials of pancreatic cancer cells.

### NR5A2 overexpression enhances the malignant potentials of pancreatic cancer cells

**Fig. 3:**
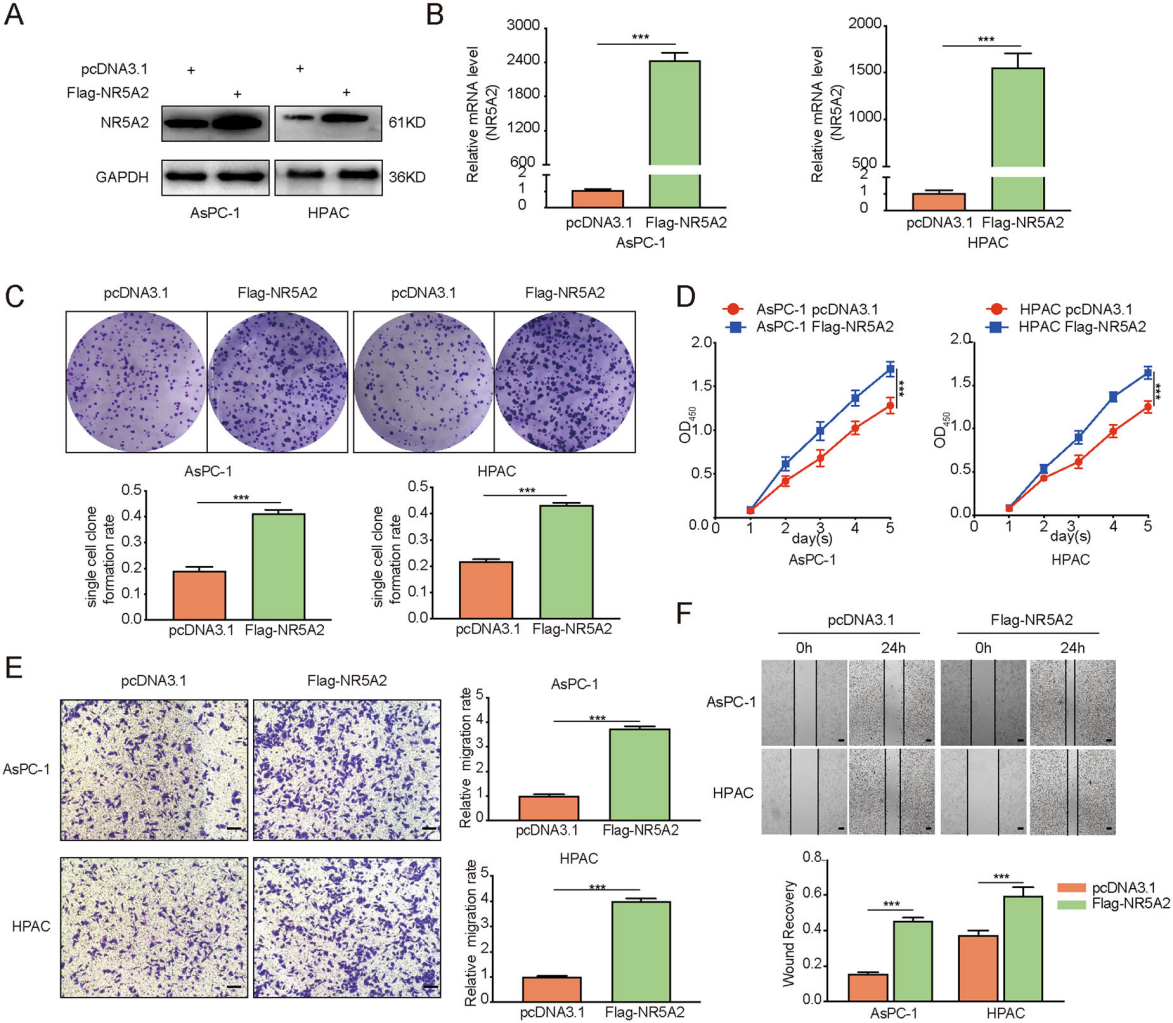
Overexpressed NR5A2 promoted the potential malignancy of pancreatic cancer cells. **A**, **B**. Western blot (**A**) and RT-qPCR (**B**) analysis to show NR5A2 expression in AsPC-1 and HPAC cells infected with pcDNA3.1 or NR5A2 plasmid. GAPDH served as an internal reference. Data presented as the mean ± SD of three independent experiments. Statistical analyses were performed using an unpaired *t* test. **C**–**F** AsPC-1 and HPAC cells infected with pcDNA3.1 or NR5A2 plasmid were harvested for colony formation assay (**C**), MTS assay (**D**, *n* = 5), transwell invasion assay (**E**), and wound-healing assay (**F**), scale bars: 100 μm. Each bar represents the mean ± SD of three independent experiments. **p < 0.01; ***p < 0.001, one-way ANOVA.

As described above, *NR5A2* silencing inhibited pancreatic cancer cell proliferation, migration, and invasion, we sought to determine whether NR5A2 overexpression would increase tumor cell malignancy by stably overexpressing NR5A2 in AsPC-1 and HPAC cells (Fig. 3A, B). Colony formation and MTS assays indicated that NR5A2 overexpression accelerated the proliferation of pancreatic cancer cells (Fig. 3C, D). Moreover, NR5A2 overexpression increased the invasion and migration potentials of pancreatic cancer cells (Fig. 3E, F). These data indicated that NR5A2 overexpression may increase the malignant potentials of pancreatic cancer cells.

### BRD4 transcriptionally activates NR5A2 expression in pancreatic cancer cells

**Fig. 4:**
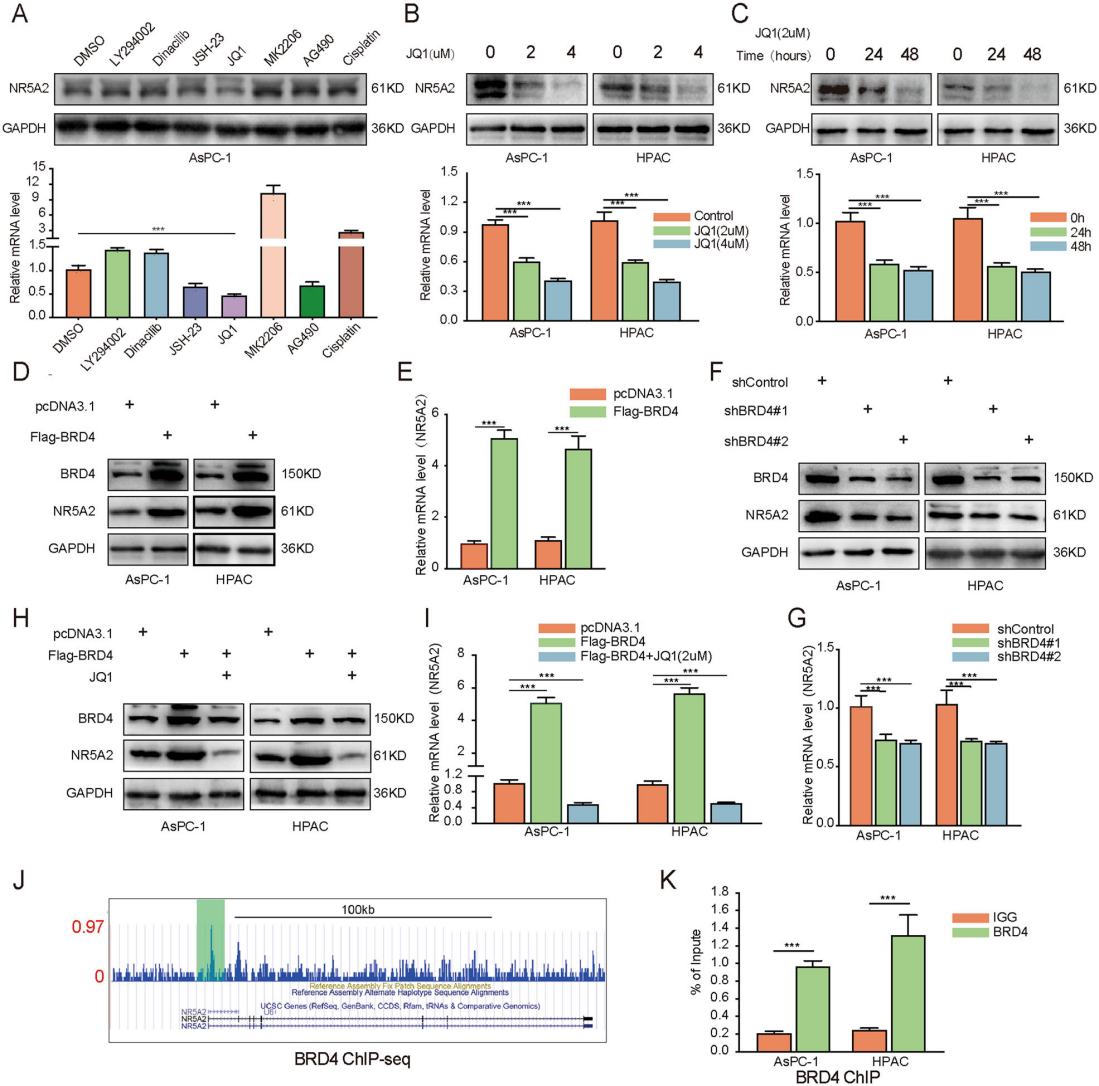
BRD4 transcriptionally activates NR5A2 expression in pancreatic cancer cells. **A** Western blot analysis and RT-qPCR assay to show the NR5A2 expression in AsPC-1 cells treated with different inhibitors. GAPDH served as an internal reference. Data presented as the mean ± SD of three independent experiments. ***p < 0.001, one-way ANOVA. **B** AsPC-1 and HPAC cells treated with JQ-1 for different concentrations were harvested for Western blots and RT-qPCR analysis. GAPDH served as an internal reference. Data presented as the mean ± SD of three independent experiments. ***p < 0.001, one-way ANOVA. **C** AsPC-1 and HPAC cells treated with JQ-1 for different durations were harvested for Western blots and RT-qPCR analysis. GAPDH served as an internal reference. Data presented as the mean ± SD of three independent experiments. ***p < 0.001, one-way ANOVA. **D**, **E** AsPC-1 and HPAC cells infected with pcDNA3.1 or BRD4 plasmid were harvested for Western blots (**D**) and RT-qPCR analysis (**E**). GAPDH served as an internal reference. All the data are mean ± S.D. from experiments with three replicates. ***p < 0.001. Statistical analyses were performed using unpaired *t* test. **F**, **G** AsPC-1 and HPAC cells infected with shControl or shNR5A2s were harvested for Western blots (**F**) and RT-qPCR analysis (**G**). GAPDH served as an internal reference. All the data are mean ± S.D. from experiments with three replicates. ***p < 0.001, one-way ANOVA. **H**, **I** AsPC-1 and HPAC cells infected with pcDNA3.1 or BRD4 plasmid and treated with or without JQ-1 were harvested for Western blots (**H**) and RT-qPCR analysis (**I**). GAPDH served as an internal reference. All the data are mean ± S.D. from experiments with three replicates. ***p < 0.001, one-way ANOVA. **J** A UCSC Genome Browser was used to show BRD4 ChIP-seq signal profiles in the NR5A2 gene locus^13^. **K** ChIP-qPCR of BRD4 in AsPC-1 and HPAC cells. All data are shown as the mean values ± SD from three replicates. ***p < 0.001, unpaired *t* test.

To further investigate the mechanisms driving the overexpression of NR5A2 in pancreatic cancer cells, we treated AsPC-1 cells with various inhibitors and examined the changes in NR5A2 expression levels. The IC50 of pancreatic cancer cells to these inhibitors was used as a working concentration in this experiment (Fig. S1). Western blot analysis showed that the treatment of bromodomain-containing protein 4 (BRD4) inhibitor, JQ1, significantly decreased the expression of NR5A2 on mRNA and protein level (Fig. 4A). Moreover, the ability of JQ1 to suppress NR5A2 expression in pancreatic cancer cells was dose-dependent (Fig. 4B) and time-dependent (Fig. 4C).

Given that the BRD4 inhibitor JQ1 downregulated NR5A2 expression, we speculated that BRD4 might transcriptionally activate the expression of *NR5A2*. BRD4 overexpression in pancreatic cancer cells significantly upregulated NR5A2 both at the mRNA and protein levels (Fig. 4D, E). Consistently, *BRD4* silencing profoundly decreased NR5A2 expression on mRNA and protein levels in pancreatic cancer cells (Fig. 4F, G). We also found that JQ1 treatment attenuated the ability of BRD4 overexpression in the point of NR5A2 expression (Fig. 4H, I). Furthermore, we identified a remarkable BRD4-binding peak at the promoter region of the *NR5A2* gene by analyzing a publicly available BRD4 ChIP-seq data^13^ (Fig. 4J). This result was further confirmed by chromatin Immunoprecipitation quantitative real-time PCR (ChIP-qPCR) analysis in pancreatic cancer cells (Fig. 4K). Taken together, these results demonstrate that BRD4 transcriptionally activates NR5A2 expression in pancreatic cancer.

### NR5A2 transcriptionally regulates GDF15 in pancreatic cancer cells

**Fig. 5:**
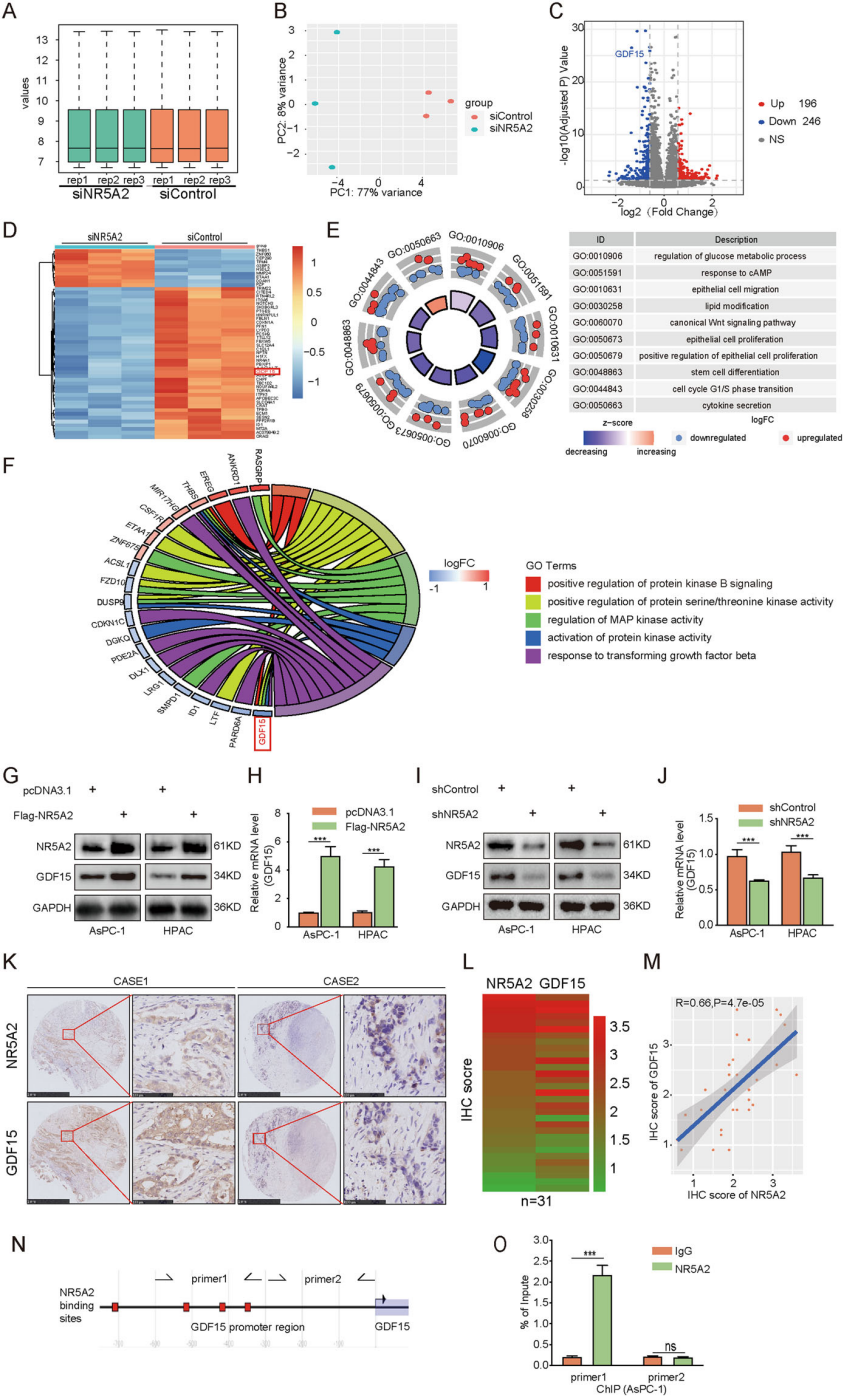
Overexpressed NR5A2 transcriptionally increased the expression of GDF15 in pancreatic cancer. **A**, **B** PANC-1 cells infected by siControl or siNR5A2 were harvested for Transcriptome RNA sequencing. Boxplot (**A**) and Principle Component Analysis (**B**) to show the quality control of RNA sequencing. **C**, **D** Volcano map (**C**) and heat map (**D**) to show that GDF15 was significantly downregulated in NR5A2 silenced PANC-1 cells. **E** Gocircle plot to show that several cancer-related GO terms were downregulated in NR5A2-silenced PANC-1 cells. **F** GO Chord plot indicated that GDF15 was a component of multiple downregulated tumor-promoting gene sets. **G**, **H**. AsPC-1 and HPAC cells infected with shControl or shNR5A2 were harvested for Western blots (**G**) and RT-qPCR analysis (**H**). GAPDH served as an internal reference. All the data are mean ± S.D. from experiments with three replicates. ***p < 0.001. Statistical analyses were performed using an unpaired *t* test. **I**, **J**. AsPC-1 and HPAC cells infected with pcDNA3.1 or NR5A2 plasmid were harvested for Western blots (**I**) and RT-qPCR analysis (**J**). GAPDH served as an internal reference. All the data are mean ± S.D. from experiments with three replicates. ***p < 0.001, unpaired *t* test. **K**–**M** IHC Images (**K**) of NR5A2 and GDF15 staining using TMA tissue sections (*n* = 31). The scale bars were shown as indicated. Heatmap (**L**) and dot plot (**M**) to show the correlation of IHC scores for the expression of the NR5A2 and GDF15 proteins in pancreatic cancer patient specimens. (*r* = 0.66 for spearman correlation coefficients, *p* = 4.7e−05). **N** The GDF15 promoter sequences and putative NR5A2 binding sites were obtained from the Eukaryotic Promoter Database (EPD) (https://epd.epfl.ch//index.php). **O** ChIP-qPCR of NR5A2 in AsPC-1 and HPAC cells. All data are shown as the mean values ± SD from three replicates. ns not significant; ***p < 0.001, unpaired *t* test.

To elucidate how NR5A2 promotes pancreatic cancer progression, we performed RNA-seq analysis in control and NR5A2-silenced PANC-1 cells (Fig. 5A, B). A total of 196 upregulated genes and 246 downregulated genes (including *GDF15*) were identified in PANC-1 cells after *NR5A2* silencing (Fig. 5C, D). Gene ontology (GO) analysis showed that several cancer-related GO terms were downregulated in NR5A2-silenced PANC-1 cells (Fig. 5E). Further analysis indicated GDF15 is a component of multiple downregulated tumor-promoting gene sets (Fig. 5F). Hence, we sought to investigate that if NR5A2 promotes pancreatic cancer cell malignancy by activating GDF15 expression. Western blot and RT-qPCR showed that the mRNA and protein levels of GDF15 were significantly increased in NR5A2 overexpressed pancreatic cancer cells (Fig. 5G, H). Accordingly, *NR5A2* silencing significantly decreased the expression of GDF15 (Fig. 5I, J). IHC analysis on a TMA of pancreatic cancer samples indicated that there exists a positive correlation between the expression of NR5A2 and GDF15 (Fig. 5K–M). Analysis in EPD identified NR5A2-binding sites in the promoter of GDF15, and this finding was confirmed by ChIP-qPCR analysis (Fig. 5N–O). These results demonstrate that NR5A2 functions as a positive transcriptional regulator of GDF15 in pancreatic cancer cells.

### NR5A2 enhances the malignancy of pancreatic cancer cells in vitro by upregulating GDF15

**Fig. 6:**
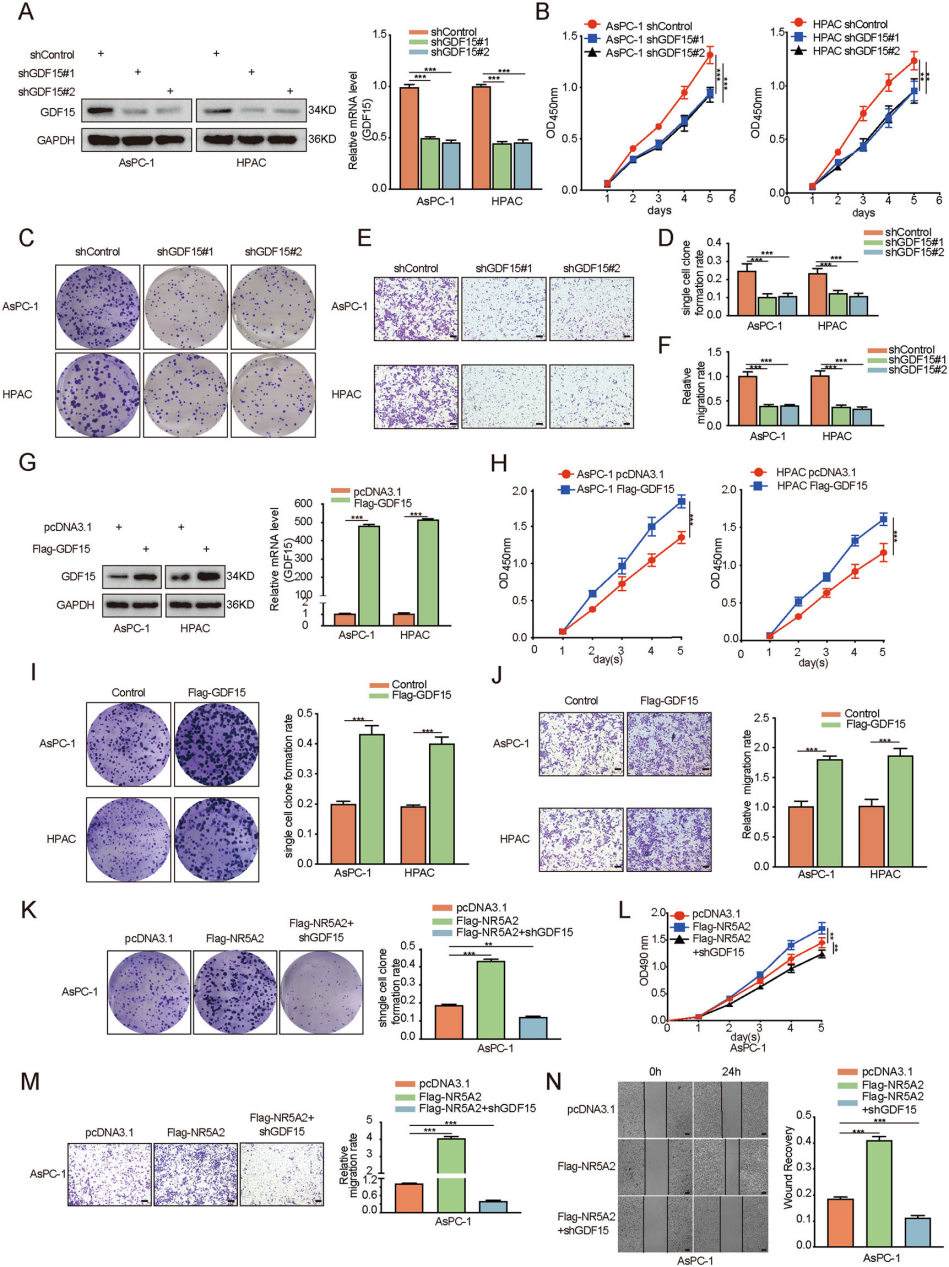
NR5A2 promoted the potential malignancy of pancreatic cancer by upregulating GDF15 expression in vitro. **A** Western blot analysis and RT-qPCR to show the GDF15 expression in AsPC-1 and HPAC cells infected with pcDNA3.1 or GDF15 plasmid. GAPDH served as an internal reference. Data presented as the mean ± SD of three independent experiments, ***p < 0.001, one-way ANOVA. **B**–**F** AsPC-1 and HPAC cells infected with pcDNA3.1 or GDF15 plasmid were harvested for MTS assay (B, *n* = 5), colony formation assay (**C**, **D**), and transwell invasion assay (**E**, **F**). Each bar represents the mean ± SD of three independent experiments. ***p < 0.001, one-way ANOVA. **G** Western blot analysis and RT-qPCR to show the GDF15 expression in AsPC-1 and HPAC cells infected with shControl or shGDF15. GAPDH served as an internal reference. Data presented as the mean ± SD of three independent experiments, ***p < 0.001, unpaired *t* test. **H**–**J** AsPC-1 and HPAC cells infected with shControl or shGDF15 were harvested for MTS assay (**H**, *n* = 5), colony formation assay (**I**), and transwell invasion assay (**J**), scale bars: 100 μm. Each bar represents the mean ± SD of three independent experiments. ***p < 0.001, unpaired *t* test. **K**–**M** AsPC-1 cells infected with pcDNA3.1, NR5A2, or NR5A2 + shGDF15 were harvested for colony formation assay (**K**), MTS assay (**L**, *n* = 5), Invasion assay (**M**) and wound healing assay (**N**), scale bars: 100 μm. Each bar represents the mean ± SD of three independent experiments. ***p < 0.001, one-way ANOVA.

To test if NR5A2 promotes the progression of pancreatic cancer via GDF15, we first carried out MTS, colony formation, and transwell invasion assays in pancreatic cancer cells after *GDF15* silencing or overexpression. *GDF15* silencing significantly inhibited the proliferation and invasion ability of pancreatic cancer cells (Fig. 6A–F), whereas *GDF15* overexpression remarkably enhanced pancreatic cancer cell proliferation and invasion (Fig. 6G–J). Furthermore, *GDF15* silencing attenuated cell proliferation (Fig. 6K, L), invasion (Fig. 6M), and wound healing ability (Fig. 6N) in pancreatic cancer cells overexpressing NR5A2. These results indicate that NR5A2 promotes pancreatic cancer cell proliferation and invasion by upregulating GDF15.

### BRD4 upregulates GDF15 by inducing NR5A2 expression

**Fig. 7:**
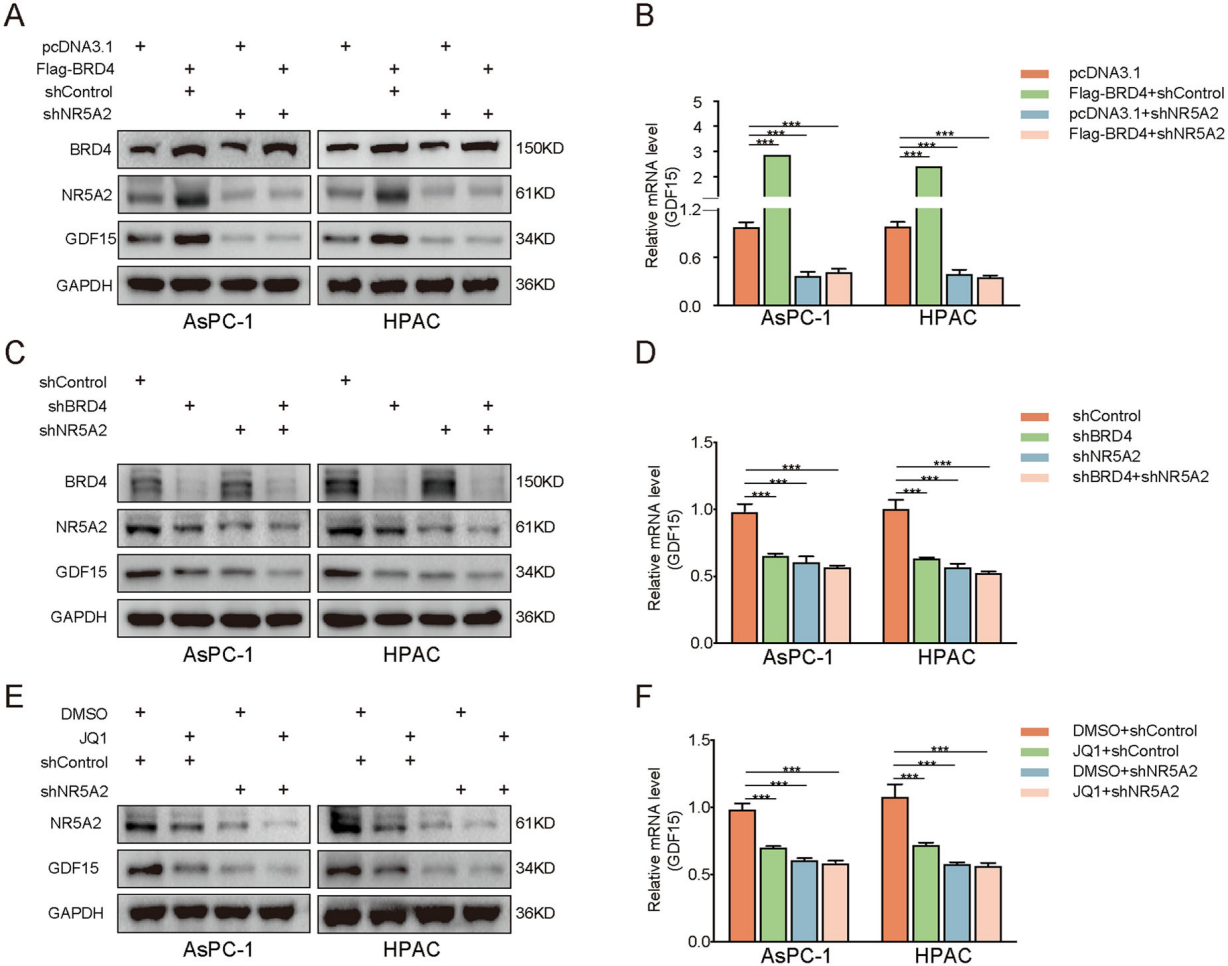
BRD4 upregulated the expression of GDF15 by inducing NR5A2 expression. **A**, **B**. Western blot analysis (**A**) and RT-qPCR (**B**) to show the GDF15 expression in AsPC-1 and HPAC cells infected with pcDNA3.1, BRD4, shNR5A2, or BRD4 + shNR5A2. GAPDH served as an internal reference. Each bar represents the mean ± SD of three independent experiments. ***p < 0.001. Statistical analyses were performed using one-way ANOVA. **C**, **D** Western blot analysis (**C**) and RT-qPCR (**D**) to show the GDF15 expression in AsPC-1 and HPAC cells infected with shControl, shBRD4, shNR5A2, or shBRD4 + shNR5A2. GAPDH served as an internal reference. Each bar represents the mean ± SD of three independent experiments. ***p < 0.001, one-way ANOVA. **E**, **F** Western blot analysis (**E**) and RT-qPCR (**F**) to show the GDF15 expression in AsPC-1 and HPAC cells infected with shControl or shNR5A2 and treated with or without JQ1. GAPDH served as an internal reference. Each bar represents the mean ± SD of three independent experiments. ***p < 0.001, one-way ANOVA.

Since BRD4 transcriptionally activates NR5A2 expression (Fig. 4), and NR5A2 induces GDF15 expression (Fig. 5). Hence, we hypothesized that BRD4 upregulated GDF15 by inducing NR5A2 expression in pancreatic cancer. Indeed, BRD4 overexpression in pancreatic cancer cells increased GDF15 expression at both the mRNA and protein levels; this effect was reversed by NR5A2 silencing (Fig. 7A, B). Notably, BRD4 inhibition in pancreatic cancer downregulated GDF15 at the mRNA and protein levels, and GDF15 expression was recovered by *NR5A2* silencing (Fig. 7C, D). Consistent phenomena were observed when we treated control cells and NR5A2 silenced cells with JQ-1 (Fig. 7E, F). Taken together, these results suggest that BRD4 upregulates GDF15 by inducing NR5A2 expression in pancreatic cancer cells.

### NR5A2 silencing inhibits tumor growth in vivo by downregulating GDF15

**Fig. 8:**
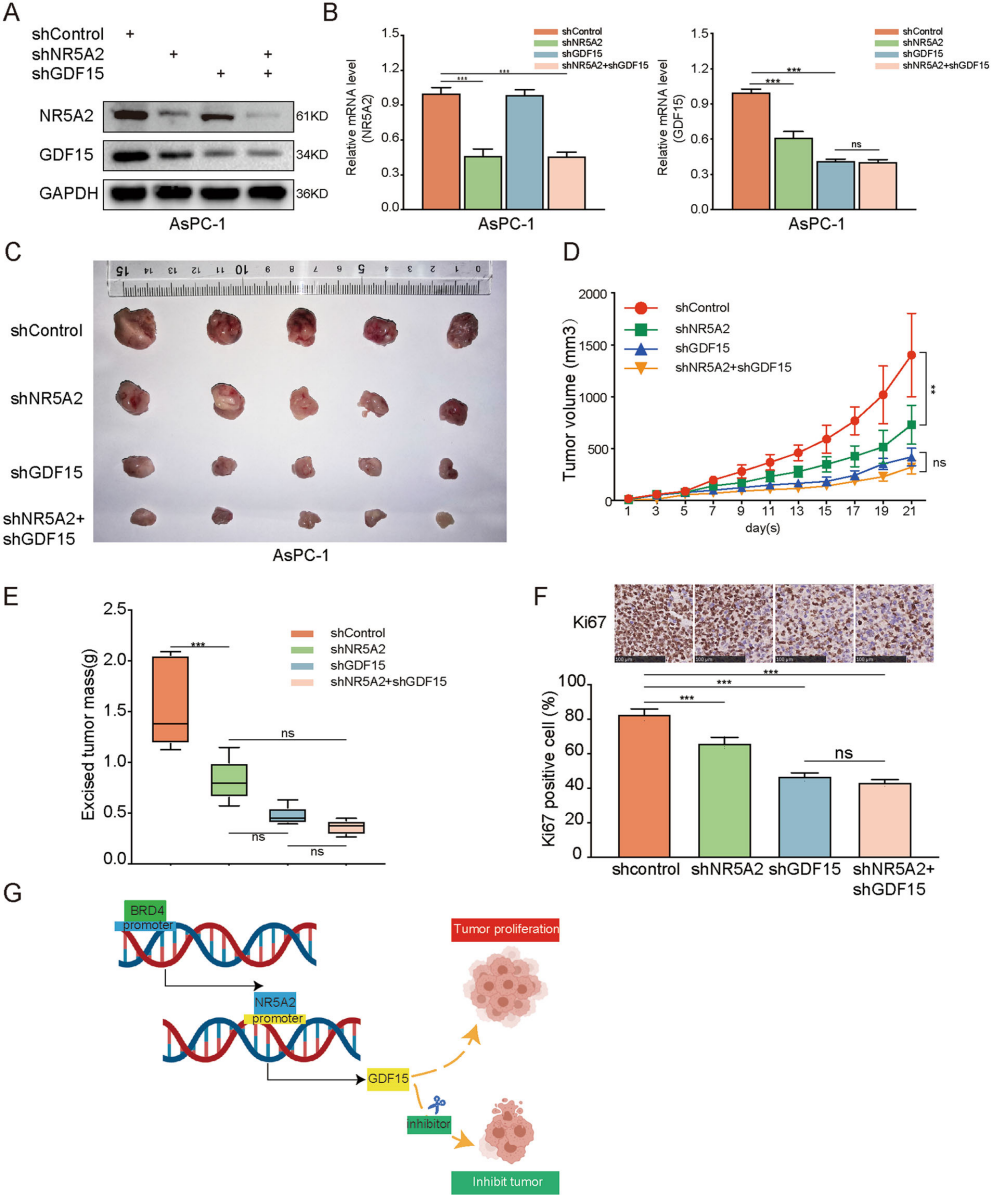
NR5A2 silencing inhibited tumor growth by downregulating GDF15 expression in vivo. **A**, **B** Western blot analysis (**A**) and RT-qPCR (**B**) to show the NR5A2 and GDF15 expression in AsPC-1 and HPAC cells infected with shControl, shNR5A2, shGDF15, or shNR5A2 + shGDF15. GAPDH served as an internal reference. Each bar represents the mean ± SD of three independent experiments. ***p < 0.001, one-way ANOVA. **C**–**E** AsPC-1 cells infected with shControl, shNR5A2, shGDF15, or shNR5A2 + shGDF15 were subcutaneously injected into nude mice. The tumors were harvested and photographed (**C**) on day 21. Data on tumor volume (**D**) and tumor mass (**E**) were shown as means ± SD (*n* = 5). Statistical analyses were performed with one-way ANOVA. ns not significant; ***p < 0.001. **F** IHC analysis for Ki-67 expression was performed in tumors harvested from xenografts, and percent of the Ki-67-positive cells were quantified, the scale bars were shown in the figure. All data were shown as mean ± SD (*n* = 5). ns not significant; **p < 0.01, one-way ANOVA. **G** The schematic diagram of this study: NR5A2 transcriptional activation by BRD4 promotes pancreatic cancer progression by upregulating GDF15.

Next, we examined the effects of the silencing of NR5A2 and GDF15 on tumor growth in vivo (Fig. 8A, B). AsPC-1 cells were then subcutaneously injected into the left flank of nude mice. Interestingly, tumors with concurrent NR5A2 and GDF15 silencing were significantly smaller than control tumors; tumors formed by shNR5A2 + shGDF15 AsPC-1 cells were similar in size to tumors formed by shGDF15 AsPC-1 cells (Fig. 8C–E). Ki-67 staining of the tumors also showed similar results (Fig. 8F). These results suggest that NR5A2 silencing inhibits tumor growth in vivo by downregulating GDF15 expression. Subsequently, we exhibited the schematic diagram of this study (Fig. 8G).

## Discussion

The orphan nuclear receptor NR5A2 participates in several biological processes, including cholesterol and glucose metabolism in the liver, resolution of endoplasmic reticulum stress, intestinal glucocorticoid production, pancreatic development, and acinar differentiation^14–16^. Recently, single nucleotide polymorphisms in NR5A2 have been associated with the risk of pancreatic cancer development^17,18^. Studies have also shown that NR5A2 cooperates with mutant KRAS to promote pancreatic cancer progression^11^. The oncogenic functions of NR5A2 remain controversial, with some studies showing that NR5A2 inhibits tumor progression^10–12^, and others suggesting that NR5A2 promotes cancer cell proliferation and invasion^19^, tumor growth and metastasis^20^, and drug resistance^21^. In this study, we confirmed that NR5A2 acts as a negative prognostic factor in pancreatic cancer and that NR5A2 promotes cell proliferation, migration, and invasion in pancreatic cancer cells in vitro and in vivo. Hence, our data suggest NR5A2 is a promising therapeutic target in pancreatic cancer.

BRD4 is a member of the bromodomain and extra-terminal (BET) domain family, which binds to acetylated lysine residues on histones and non-histone proteins^22^. BRD4 is involved in various biological processes, including cell cycle, cell proliferation, invasion, and autophagy^23^. In addition to interacting with acetylated histones, BRD4 also promotes cancer progression by regulating the interaction with transcription factors in cancer cells^24–26^. Wang et al.^27^ showed that BRD4 was significantly upregulated in pancreatic cancer cell lines and that BRD4 promoted cell proliferation and gemcitabine resistance in pancreatic cancer. Moreover, BRD4 promoted tumor progression by inducing the expression of several oncogenes and immunomodulatory genes, including c-Myc^24^, PD-L1^28^, and B7-H3^29^. Recently, several small molecule inhibitors, such as JQ1 and I-BET762, have been developed to specifically target the bromodomains of BET proteins^30^.

There are existing studies related to the regulation of NR5A2 expression. Zhu et al.^31^ also revealed that the downregulation of microRNA-27b-3p increased NR5A2 expression in breast cancer; miR-374b has been shown to inhibit colon cancer cell proliferation and invasion through the downregulation of NR5A2 expression^19^. Furthermore, microRNA-219-5p and microRNA-136 have been demonstrated to regulate the expression level of NR5A2 in cancer cells^32,33^. However, it is possible that NR5A2 expression is regulated by additional mechanisms that are yet to be elucidated. In this study, we identified BRD4 as a transcriptional activator of NR5A2 in pancreatic cancer cells and provided another viable strategy to target oncogenic NR5A2.

The nuclear receptor NR5A2 is a transcription factor regulating the expression of the nuclear receptor NR5A2 is a transcription factor regulating the expression of cell type-specific and tissue-specific target genes. NR5A2 promotes tumor growth and metastasis by activating Wnt/beta-catenin signaling^20^. Chromatin immunoprecipitation analyses have shown that NR5A2 directly regulates *CDKN1A* transcription by binding to the *CDKN1A* promoter^34^. Ye et al.^35,36^ also showed that NR5A2 upregulated the expression of Nanog. In this study, we showed that NR5A2 promoted pancreatic cancer progression by inducing GDF15 expression, suggesting that the pro-tumorigenic roles of NR5A2 and BRD4 in pancreatic cancer could be reversed by GDF15 inhibition, making GDF15 a promising therapeutic target (Fig. 8G).

GDF15, also known as NSAID-activated gene-1 (NAG-1), has been implicated in numerous biological processes and diseases, including energy homeostasis, cancer, inflammation, cardiovascular diseases, and obesity^37,38^. Elevated GDF15 mRNA and protein levels have been reported in cancer biopsies^39,40^, suggesting a pro-tumorigenic role for GDF15. However, controversy exists regarding the role of GDF-15 in cancer development and progression, which may depend on the tumor type and study model^41,42^. In our study, we found that GDF15 was transcriptionally induced by NR5A2 and that GDF15 overexpression promoted pancreatic cancer cell proliferation and invasion.

In conclusion, our study suggests that NR5A2 promoted cell proliferation, migration, and invasion in pancreatic cancer cells. In addition, we showed that the expression of NR5A2 in pancreatic cancer cells was regulated by BRD4. We further confirmed that NR5A2 induced the transcription of *GDF15* by directly binding to the promoter region of *GDF15* gene. More importantly, we demonstrated that NR5A2 promoted pancreatic cancer progression by upregulating GDF15 expression in vitro and in vivo. Overall, our study suggests that BRD4/NR5A2/GDF15 axis is a promising therapeutic target in pancreatic cancer.

## Materials and methods

### Cell culture

AsPC-1(#TCHu 8) and 293T(#SCSP-502) cell lines were purchased from the National Collection of Authenticated Cell Cultures (Shanghai, China). HPAC cell line (#CRL-2119) was purchased from ATCC. Cell lines were authenticated periodically and mycoplasma contamination was regularly examined. AsPC-1 cells were cultured in RMPI-1640 (#88365, Thermo Fisher), 293T cells were maintained in Dulbecco modified Eagle medium (DMEM) (#30030, Thermo Fisher), both media are supplemented with 10% fetal bovine serum (FBS) (#10099141, Thermo Fisher) and 1% penicillin/streptomycin. HPAC cells were maintained in 1∶1 mixture of DMEM and Ham’s F12 medium supplemented with 5% FBS and 1% penicillin/streptomycin. All cell lines were kept at 37 °C in a 5% CO_2_ incubator.

### Antibodies and chemicals

NR5A2 antibody (#22460-1-AP, working dilution 1:800) and GDF15 antibody (#27455-1-AP, working dilution 1:1000) were purchased from Proteintech. A BRD4 antibody (#13440, working dilution 1:800) was acquired from Cell Signaling Technology. A GAPDH antibody (#ab8245, working dilution 1:3000) was obtained from Abcam. JQ1 (#HY-13030), LY-294002 (#HY-10108), JSH23 (#HY-13982), MK2206 (#C2206, Sigma-Aldrich), AG490 (#HY-12000), Cisplatin (#HY-17394), and Dinaciclib (#HY-10492) were procured from MedChamExpress (USA).

### Immunohistochemistry

Tissue microarray was purchased from Outdo Biobank (Shanghai, China) (HPan-Ade060CD-01). IHC analysis was performed with NR5A2 (#22460-1-AP, working dilution 1:5000, Proteintech), GDF15 (#27455-1-AP, working dilution 1:500, Proteintech), Ki67(27309-1-AP, working dilution 1:3000, Proteintech) antibodies. Two independent pathologists, who were uninformed about the patient data and histopathological features of the samples, were responsible for reviewing and scoring the degree of immunostaining separately. Staining intensity scoring was performed as described previously^43^.

### Western blot analysis

Cells were harvested and lysed for 30 min with lysis buffer containing 1% protease and phosphatase inhibitors on ice (#ST505, #P1081, # P1082, Beyotime). The resulting cell lysates were centrifuged at 12,000 rpm for 20 min at 4 °C to remove undissolved impurities and collect the supernatants. Protein concentration was then determined using a protein quantification kit (#P0012S, Beyotime) to ensure that equal amounts of total protein were loaded in each well of sodium dodecyl sulfate-polyacrylamide gel electrophoresis gels. The gels were subsequently transferred onto PVDF membranes, blocked in 5% nonfat milk for 1 h at room temperature, and incubated with primary antibody overnight at 4 °C. On the following day, the membranes were washed with 1× TBST for 30 min and incubated with a secondary antibody for 1 h. After incubation, the membranes were again washed 3 times with PBS and exposed to X-ray films using ECL detection reagents (#WP20005, Thermo Fisher). Ethical approval for the use of human tissues (12 pairs of matched pancreatic cancer and adjacent noncancerous tissues) was obtained by the local ethics committee (Tongji Medical College, China). Written informed consent was acquired from all patients before surgery.

### Quantitative RT-PCR assay

Total RNA was prepared using a Trizol reagent (#15596026, Invitrogen). RNA samples (1 μg) were reverse-transcribed using a PrimeScript^™^ RT reagent Kit (#RR047A, TAKARA, JPN). Quantitative real-time PCR was performed using a TB Green™ Fast qPCR Mix kit (#RR430A, TAKARA, JPN). All the values obtained were normalized by GAPDH, and the 2^−^^ΔCt^ method was used to quantify fold change. The primer sequences for RT-qPCR are shown in supplementary data 1 (Table S1).

### RNA sequence

Total RNA was prepared using a Trizol reagent (#15596026, Invitrogen), and RNA sequence was performed by NOVOGENE (Beijing, China) based on the Illumina sequencing platform. RNA degradation and contamination were monitored on 1% agarose gels and RNA purity was checked using the NanoPhotometer spectrophotometer (IMPLEN, CA, USA), RNA integrity was assessed using the RNA Nano 6000 Assay Kit of the Bioanalyzer 2100 system (Agilent Technologies, CA, USA). The clustering of the index-coded samples was performed on a cBot Cluster Generation System using TruSeq PE Cluster Kit v3-cBot-HS (Illumia) according to the manufacturer’s instructions. After cluster generation, the library preparations were sequenced on an Illumina Novaseq platform and 150 bp paired-end reads were generated.

### RNA interference

shControl and gene-specific shRNAs were procured from Sigma-Aldrich. 293T cells were transfected with shRNA plasmids and packaging plasmids (pVSV-G and pEXQV) in Lipofectamine 2000 (#11668019, Thermo Fisher). Twenty-four-hour after transfection, the Lipofectamine 2000 medium was replaced with fresh DMEM containing 10% FBS and 1 mM sodium pyruvate. Forty-eight hours post transfection, the virus culture medium was collected and added to pancreatic cancer cells and left to culture for 24 h, after which the infected cells were selected with 1 μg/ml of puromycin. shRNA sequences are shown in Supplementary Data 1 (Table S2).

### ChIP and ChIP-qPCR

ChIP was performed with a Chromatin Extraction Kit (#ab117152, Abcam) and a ChIP Kit Magnetic—One Step (#ab156907, Abcam) following the manufacturer’s instructions. Purified DNA was analyzed using real-time PCR and a TB Green^™^ Fast qPCR Mix kit (#RR430A, TAKARA, JPN) according to the manufacturer’s protocols. Primers used for ChIP-qPCR are shown in Supplementary Data 1 (Table S3).

### Colony formation assay

Tumor cell proliferation was assessed using the colony formation assay. For this procedure, AsPC-1 and HPAC cells were plated into 6-well plates (500 cells per well) and incubated in the prescribed medium with 10% FBS at 37 °C. After 12–14 days of culture, the cells were fixed in methanol for 30 min, stained with 1% Crystal Violet Staining Solution for another 30 min, and washed 3 times with PBS. In the end, the number of colonies was counted. All assays were performed in triplicates.

### Cell invasion assay

Cell invasion ability was assessed using the Transwell assay. For this test, 24-well Corning Costar inserts with 8 μm pores were used according to the manufacturer’s instructions. Tumor cells were inoculated into the Matrigel-coated invasion upper chamber of the inserts, and DMEM or RMPI-1640 containing 30% FBS was placed in the lower chambers and incubated for 12–24 h at 37 °C and 5% CO_2_. Following incubation, the cells were fixed in methanol for 30 min and then stained with a 1% Crystal Violet Staining Solution for another 30 min. Invasion cells were observed under a microscope at five fields per well. All assays were performed in triplicates.

### Wound-healing assay

AsPC-1 and HPAC cells were plated in 6-well plates and cultured for 24 h. After the 24 h of culturing, each well was scraped with a 10 μL pipette tip to create wounds, and a medium with 2% FBS was added. Wound healing was determined as the percent change in wound size over the healing period. All assays were performed in triplicates.

### MTS assay

Tumor proliferative capacity was assessed using (3-(4,5-dimethylthiazol-2-yl)-5-(3-carboxymethoxyphenyl)-2-(4-sulfophenyl)-2H-tetrazolium) (MTS reagent) (Abcam, #ab197010, USA). 2000 cells were plated in 96-well plates filled with 200 μl DMEM containing 10% FBS and cultured for 3 days. Twenty microlitres of the MTS reagent was added to each well three hours before the end of the incubation period following the manufacturer’s instructions. Absorbance in each well was measured with a microplate reader at 490 nm.

### Bioinformatic analysis

Bioinformatic analyses were carried out using the R Bioconductor (version 3.6.3). The ggplot2 package (version 3.3.2) was used to create the volcano map, the heatmap package (version 1.0.12) was used to generate the heatmap, and the clusterProfiler package^44^ (version 3.14.3) was used for enrichment analysis. The GOplot package^45^ (version 1.0.2) was used to generate Gocircle and GOChord plots displaying the relationship between a list of selected genes and terms. Survival analyses were performed using the K–M plotter (http://kmplot.com/), and statistical significance was determined using the log-rank test. UCSC genome browser was used to display BRD4 chromatic immunoprecipitation-sequencing (ChIP-seq) signals in the *NR5A2* gene locus^13^. *GDF15* promoter sequences and putative NR5A2-binding sites were obtained from the Eukaryotic Promoter Database (EPD; https://epd.epfl.ch//index.php).

### Pancreatic cancer xenografts in nude mice

BALB/c-nu mice (4–5 weeks old, male)) were purchased from Vitalriver (Beijing, China) and used for the xenograft test. AsPC-1 cells (5 × 10^6^) infected with shControl, shNR5A2, shGDF15, or shNR5A2 + shGDF15 lentivirus were dispersed in 100 μL PBS and inoculated subcutaneously into the left dorsal flank of nude mice. Power analysis was used to calculate the sample size required for animal experiments and animals were randomized to the different groups. There is no blinding of researchers or participants. Tumor sizes were assessed with a digital Vernier caliper every two days. The animals were sacrificed on day 21, and the tumors were excised and weighed. Statistical analyses were performed using a two-sided paired Student’s t-test to compare the difference in tumor weights between the two groups. Experimental procedures were performed according to guidelines set forth by the Chinese National Institutes of Health and approved by the Ethical Committee on Animal Experiments of the Huazhong University of Science and Technology in Wuhan, China.

### Statistical analysis

Statistical analyses were performed using the unpaired or paired Student’s *t* test for single comparisons and one-way ANOVA with a post hoc test for multiple comparisons. Statistical significance was evaluated using the GraphPad Prism 8 software (GradPad Software, Inc.). A *p* value < 0.05 was considered statistically significant. All the values are expressed as the mean ± SD. The variance between the statistically compared groups are similar. No estimate of variation has been performed within each group of data prior to statistical analysis.

## Supplementary information

figure s1

supplementary data1 primer.docx

supplementary data2 RNA-seq-rawcounts.txt

supplementary data3 original WB.pdf

supplementary data4 overview of transcriptome sequencing.pdf
