# Supplementary figures and images for: NR5A2 transcriptional activation by BRD4 promotes pancreatic cancer progression by upregulating GDF15
Source: Cell Death Discov. 2021 Apr 13;7:78. doi: 10.1038/s41420-021-00462-8 (PMC8044179; doi:10.1038/s41420-021-00462-8)

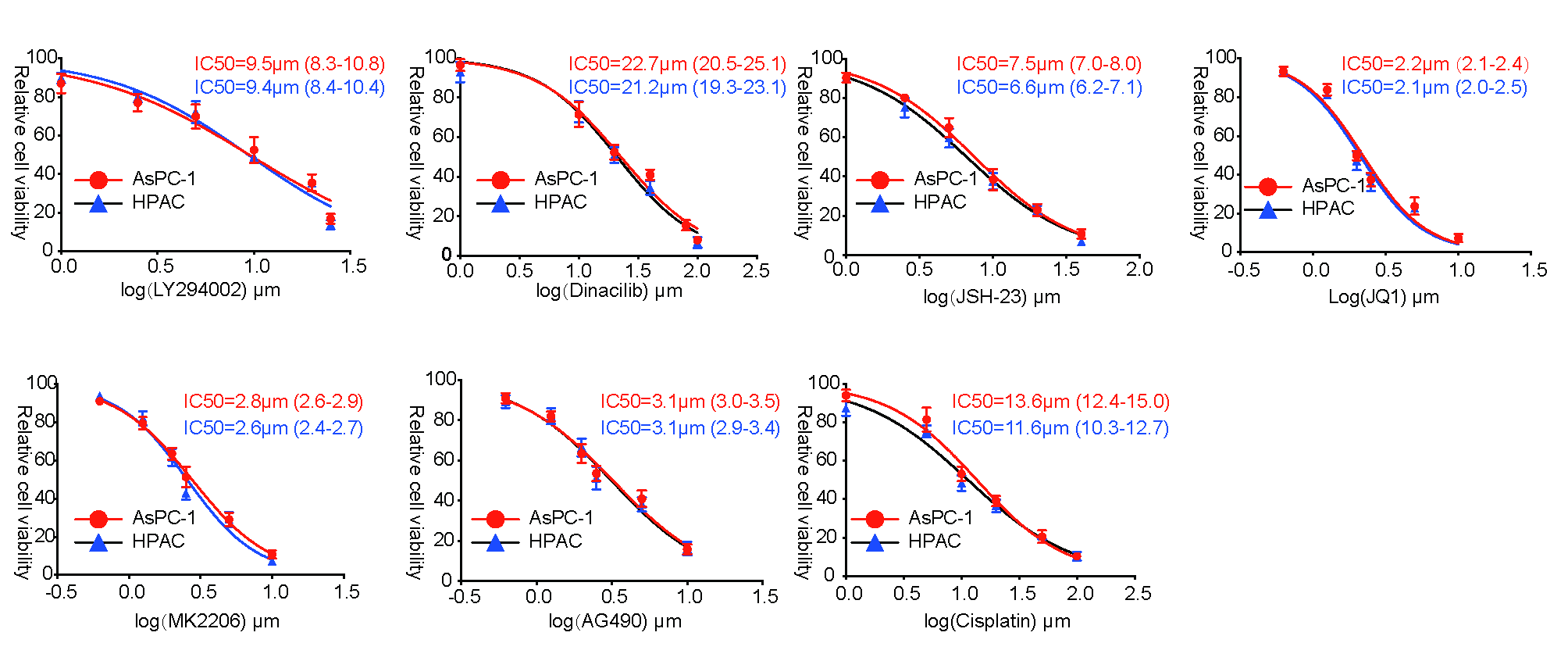

Supplement: Supplementary file 1 — figure s1 [file 41420_2021_462_MOESM1_ESM.tif]

70KD  
55KD

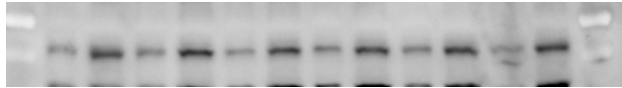

61KD NR5A2

70KD

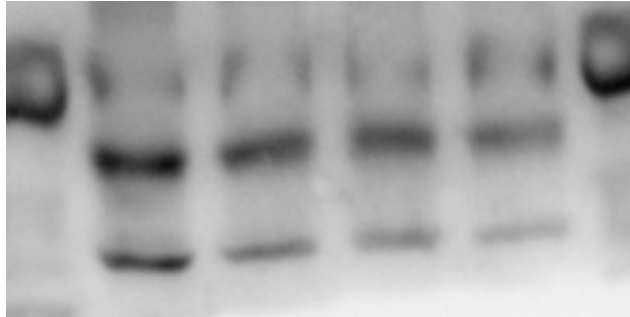

61KD NR5A2

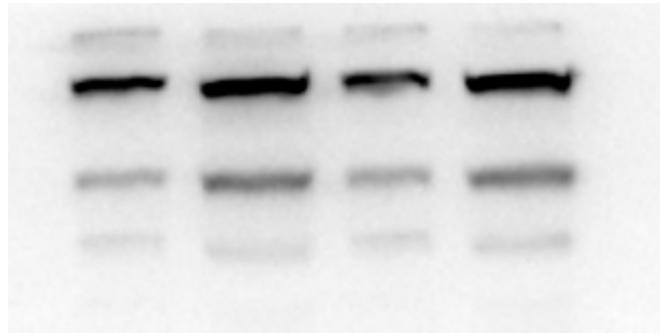

150KD BRD4

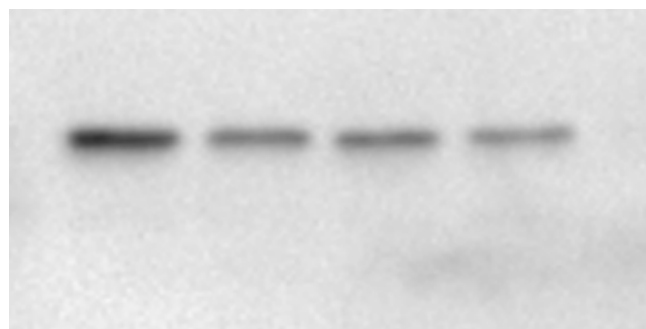

34KD GDF15

Supplement: Supplementary file 4 — supplementary data3 original WB.pdf [file 41420_2021_462_MOESM4_ESM.pdf]
