# Supplementary material for: NR5A2 transcriptional activation by BRD4 promotes pancreatic cancer progression by upregulating GDF15
Source: Cell Death Discov. 2021 Apr 13;7:78. doi: 10.1038/s41420-021-00462-8 (PMC8044179; doi:10.1038/s41420-021-00462-8)
Supplement: Supplementary file 2 — supplementary data1 primer.docx [file 41420_2021_462_MOESM2_ESM.docx]

**Supplementary Data**

**Table S1. The primer sequences for RT-qPCR.**

| Gene | Forward primer (**5**′ - 3′) | Reverse primer (**5**′ - 3′) |
| --- | --- | --- |
| GAPDH | ATGACAATGAATACGGCTACAGCA | GCAGCGAACTTTATTGATGGTATT |
| NR5A2 | CTTTGTCCCGTGTGTGGAGAT | GTCGGCCCTTACAGCTTCTA |
| BRD4 | ACCTCCAACCCTAACAAGCC | TTTCCATAGTGTCTTGAGCACC |
| GDF15 | GACCCTCAGAGTTGCACTCC | GCCTGGTTAGCAGGTCCTC |

**Table S2. The shRNA sequences.**

| Sh-NR5A2 #1 | CCGGGCGTTGTCCTTACTGTCGTTTCTCGAGAAACGACAGTAAGGACAACGCTTTTT |
| --- | --- |
| Sh-NR5A2 #2 | CCGGCCGAGTCCATAATGGGCTATTCTCGAGAATAGCCCATTATGGACTCGGTTTTT |
| Sh-BRD4 #1 | GTACCGGTGAACCTCCCTGATTACTATACTCGAGTATAGTAATCAGGGAGGTTCATTTTTTG |
| Sh-BRD4 #2 | CCGGCCTGGAGATGACATAGTCTTACTCGAGTAAGAC TATGTCATCTCCAGGTTTTTG |
| Sh-GDF15 #1 | CCGGTCTCAGATGCTCCTGGTGTTGCTCGAGCAACACCAGGAGCATCTGAGATTTTTG |
| Sh-GDF15 #2 | CCGGAGAGTTGCGGAAACGCTACGACTCGAGTCGTAGCGTTTCCGCAACTCTTTTTTG |

**Table S3. The primer sequences for ChIP-qPCR.**

| Gene | Forward primer (**5**′ - 3′) | Reverse primer (**5**′ - 3′) |
| --- | --- | --- |
| **NR5A2** | ACTGGAATTTCCCTCCGAAT | TCCCCATGTTTTCTTTTTCG |
| **GDF15 primer1** | TTTCCTGGTGAGGAAACAGG | CCCATGGGATTTCCTCTCTT |
| **GDF15 primer2** | AGGCTGGAATGGTGTCCTC | CCCTGCCTGCAGAGTAAACA |
