# Supplementary material for: NR5A2 transcriptional activation by BRD4 promotes pancreatic cancer progression by upregulating GDF15
Source: Cell Death Discov. 2021 Apr 13;7:78. doi: 10.1038/s41420-021-00462-8 (PMC8044179; doi:10.1038/s41420-021-00462-8)
Supplement: Supplementary file 5 — supplementary data4 overview of transcriptome sequencing.pdf [file 41420_2021_462_MOESM5_ESM.pdf]

提供领先的基因科技解决方案
Providing leading genomic services & solutions

1 项目简介
2 建库测序
3 信息分析流程
4 附录

诺禾致源医学转录组分析报告

合同信息	合同内容
项目编号	X101SC19120536-Z01-J008
项目名称	华中科技大学同济医学院附属协和医院100个样本转录组测序分析技术服务（委托）合同
报告时间	2020-07-25
报告编号	X101SC19120536-Z01-J008-B8-16

1 项目简介

转录组是指特定组织或细胞在某个时间或某个状态下转录出来的所有RNA的总和，主要包括mRNA和非编码RNA。转录组测序是基于Illumina测序平台，研究特定组织或细胞在某个时期转录出来的所有mRNA，是基因功能与结构研究的基础，对理解生物体的:发育和疾病的发生具有重要作用[1]。随着基因测序技术的发展以及测序成本的降低，RNA-seq凭借高通量、高灵敏度、应用范围广等优势，已成为转录组研究的主要方法。RNA-seq技术流程主要包含两个部分：建库测序和生物信息分析。

研究中涉及的方法见中文版methods (src/methods_Chinese.pdf)和英文版methods (src/methods.pdf)

2 建库测序

从RNA样品提取到最终数据获得，样品检测、建库、测序等每一环节都会直接影响数据的数量和质量，从而影响后续数据分析的结果。为从源头保证测序数据准确可靠，诺禾致源在数据的所有生产环节都严格把关，从根源上确保高质量数据的产出。建库测序的流程图如下所示。

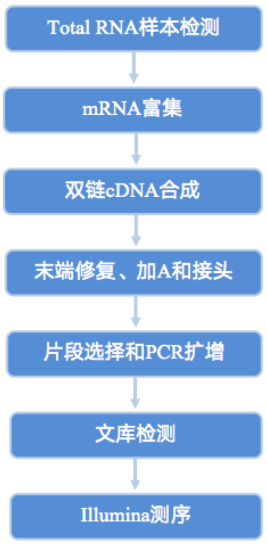

提供领先的基因科技解决方案
Providing leading genomic services & solutions
北京诺禾致源科技股份有限公司

2.1 RNA提取与检测

诺禾致源采用标准提取方法从组织或细胞中提取RNA，随后对RNA样品进行严格质控，质控标准主要包括以下3个方面。

- 琼脂糖凝胶电泳：分析样品RNA完整性及是否存在DNA污染。
- NanoPhotometer spectrophotometer：检测RNA纯度（OD260/280及OD260/230比值）。
- Agilent 2100 bioanalyzer：精确检测RNA完整性。

注：以RNA完整性和总量为主要参考指标。

2.2 文库构建与质检

mRNA的获取主要有两种方式：一是利用真核生物大部分mRNA都带有polyA尾的结构特征，通过Oligo(dT)磁珠富集带有polyA尾的mRNA。二是从总RNA中去除核糖体RNA，从而得到mRNA。随后在NEB Fragmentation Buffer中用二价阳离子将得到的mRNA随机打断，按照NEB普通建库方式或链特异性建库方式进行建库[2]。

- NEB普通建库：以片段化的mRNA为模版，随机寡核苷酸为引物，在M-MuLV逆转录酶体系中合成cDNA第一条链，随后用RNaseH降解RNA链，并在DNA polymerase I 体系下，以dNTPs为原料合成cDNA第二条链。纯化后的双链cDNA经过末端修复、加A尾并连接测序接头，用AMPure XP beads筛选250-300bp左右的cDNA，进行PCR扩增并再次使用AMPure XP beads纯化PCR产物，最终获得文库。建库用试剂盒为**NEBNext® Ultra™ RNA Library Prep Kit for Illumina®**。
- 链特异性建库：逆转录合成cDNA第一条链方法与NEB普通建库方法相同，不同之处在于合成第二条链时，dNTPs中的dTTP由dUTP取代，之后同样进行cDNA末端修复、加A尾、连接测序接头和长度筛选，然后先使用USER酶降解含U的cDNA第二链再进行PCR扩增并获得文库。链特异性文库具有诸多优势，如相同数据量下可获取更多有效信息；能获得更精准的基因定量、定位与注释信息；能提供反义转录本及每一isoform中单一exon的表达水平。建库所用试剂盒为**NEBNext® Ultra™ Directional RNA Library Prep Kit for Illumina®**。

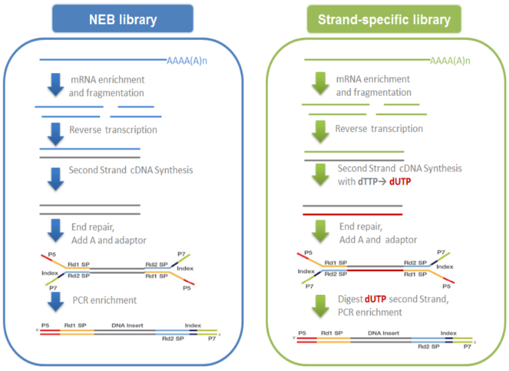

图2.2 文库构建原理示意图

注：测序接头：包括P5/P7，index和Rd1/Rd2 SP三个部分（如上图所示）。其中P5/P7是PCR扩增引物及flow cell上引物结合的部分，index提供区分不同文库的信息，Rd1/Rd2 SP即read1/read2 sequence primer，是测序引物结合区域，测序过程理论上由Rd1/Rd2 SP向后开始进行。

文库构建完成后，先使用Qubit2.0 Fluorometer进行初步定量，稀释文库至1.5ng/ul，随后使用Agilent 2100 bioanalyzer对文库的insert size进行检测，insert size符合预期后，qRT-PCR对文库有效浓度进行准确定量（文库有效浓度高于2nM），以保证文库质量。

提供领先的基因科技解决方案
Providing leading genomic services & solutions
北京诺禾致源科技股份有限公司

2.3 上机测序

库检合格后，把不同文库按照有效浓度及目标下机数据量的需求pooling后进行Illumina测序。测序的基本原理是边合成边测序（Sequencing by Synthesis）。在测序的flow cell中加入四种荧光标记的dNTP、DNA聚合酶以及接头引物进行扩增，在每一个测序簇延伸互补链时，每加入一个被荧光标记的dNTP就能释放出相对应的荧光，测序仪通过捕获荧光信号，并通过计算机软件将光信号转化为测序峰，从而获得待测片段的序列信息。测序过程如下图所示。

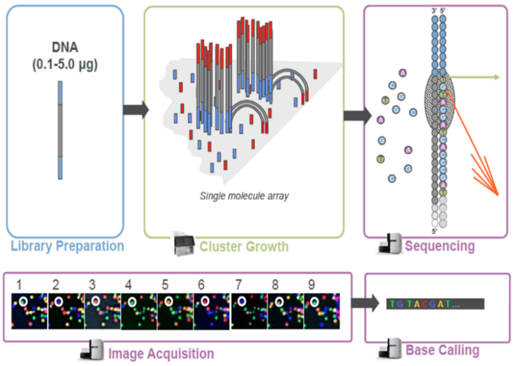

图2.3 Illumina测序原理示意图

2.3.1 建库测序常见问题和解答

相关名词解释

- **adapter**：接头，用于上机测序。建库时引入的接头序列与测序芯片（flow cell）上固定的接头相互识别
- **index**：测序的标签，用于测定混合样本，通过每个样本添加的不同标签进行数据区分，鉴别测序样品
- **Single-read**：单端测序,首先将RNA随机打断，引物序列连接到随机片段的一端，然后末端加上接头，将片段固定在flowcell上生成DNA簇，上机测序单端读取序列，下机数据只有一个read。
- **Paied-end**：双端测序，构建文库时在两端的接头上都加上测序引物结合位点，正向链完成测序后，新合成的部分链因变性被去除，3'端解封，双链DNA簇通过桥式扩增而重新形成。正向链在其链的基部切离，仅留下新合成的反向链与芯片结合，在形成配对序列数据之前对反向链测序。下机数据有成对的read1和read2。

3 信息分析流程

RNA-seq的核心是基因表达差异的显著性分析，使用统计学方法，比较两个条件或多个条件下的基因表达差异，从中找出与条件相关的特异性基因，然后进一步分析这些特异性基因的生物学意义，分析过程包括质控、比对、定量、差异显著性分析、功能富集等环节。另外可变剪接，变异位点，融合基因预测也是RNA-seq的重要分析内容。同时，诺禾致源根据不同的研究需求，推出转录组个性化分析内容，如基因共表达网络构建（WGCNA）、体细胞突变检测等。信息分析流程如下图所示：

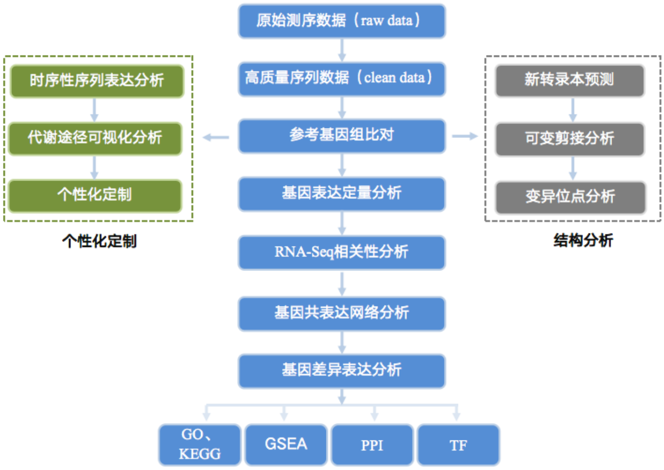

图3.1 RNA-seq信息分析技术流程

对于上图分析内容，若其存在于合同信息分析内容中，则进行此项分析；若不存在，则不进行。

3.1 数据质控

3.1.1 测序数据说明

测序片段被高通量测序仪测得的图像数据经CASAVA碱基识别转化为序列数据（reads），文件为fastq格式，其中主要包含测序片段的序列信息以及其对应的测序质量信息。

fastq格式文件中每个read由四行描述信息组成，如下所示：

```
@ST-E00310:278:HF3GJALXX:5:1101:6745:1924 1:N:0:ACGCTCGA
TTTGGGCCCTTGGCAATGAATGTTGCCACCACTGTTCTGGGTGCAGAGGGGAAATGG
+
AA&lt-FJFJ7J&ltJJFFJJFJJJ&ltFJJJJJJJFJJJJFJJJJJAFJ7FJJJJ
```

上述文件中第一行以“@”开头，随后为Illumina测序标识符(Squence Identifiers)和描述文字；第二行是测序片段的碱基序列；第三行以“+”开头，随后为Illumina测序标识符(也可为空)；第四行是测序片段每个碱基相对应的测序质量值，该行中每个字符对应的ASCII值减去33，即为该碱基的测序质量值。

3.1.2 测序错误率分布

测序过程本身存在机器错误的可能性，测序错误率分布检查可以反映测序数据的质量，序列信息中每个碱基的测序质量值保存在fastq文件中。如果测序错误率用e[3]表示，Illumina的碱基质量值用Q_{phred}表示，则有：Q_{phred}=-10log10(e)。Illumina Casava 1.8版本碱基识别与Phred分值之间的简明对应关系见下表。

表3.1 Illumina Casava 1.8版本碱基识别与Phred分值之间的简明对应关系

Copy Print Download Search:

Phred分值	不正确的碱基识别	碱基正确识别率	Q.sorce
10	1/10	90%	Q10
20	1/100	99%	Q20
30	1/1000	99.9%	Q30
40	1/10000	99.99%	Q40

Showing 1 to 4 of 4 entries Previous 1 Next

当前RNA-seq测序技术，测序错误率分布存在以下两个特征。

- 测序错误率随着测序序列(Sequenced Reads)长度的增加而升高。这是由测序过程中化学试剂的消耗导致的，为Illumina高通量测序平台所具有的特征。
- 前6个碱基具有较高的测序错误率，此长度恰好为RNA-seq建库过程中反转录所需的随机引物长度。前6个碱基测序错误率较高是因为随机引物和RNA模版的不完全结合(Jiang et al.)。

样本测序错误率分布见结果文件QC/1.Error，我们同时提供PDF和svg两种形式的图片文件。

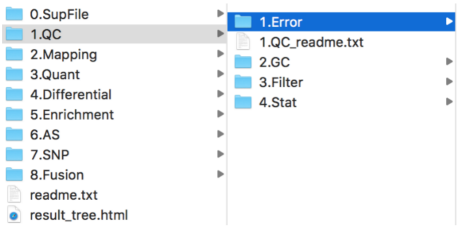


```
##
## Attaching package: 'shiny'
```

```
## The following objects are masked from 'package:DT':
##
##     dataTableOutput, renderDataTable
```

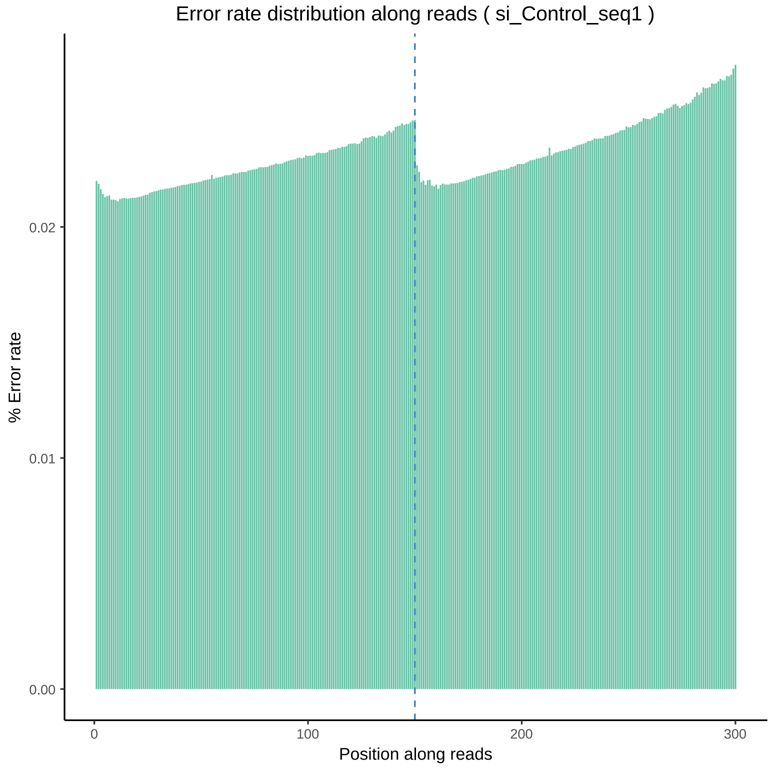

图3.2 测序数据错误率分布

图中横坐标为reads的碱基位置，纵坐标为单碱基错误率

3.1.3 GC含量分布

核苷酸序列中鸟嘌呤(G)和胞嘧啶(C)所占的比例称为GC含量。GC含量在物种间存在一定特异性，但由于反转录过程中所使用的6bp随机引物，会引起前几位碱基在核苷酸组成上有一定偏好性，产生正常波动，随后则趋于稳定。对于NEB普通建库方法，由于序列的随机性打断和双链互补等原则，理论上测序读段在每个位置的GC及AT含量应分别相等，且在整个测序过程基本稳定不变，呈水平线。而对于链特异性建库而言，由于只保留了单链信息，可能会出现AT分离或GC分离现象。本项目各个样本的GC含量分布如下图所示，见结果文件：QC/1.GC，我们同时提供PDF和svg两种形式的图片文件。

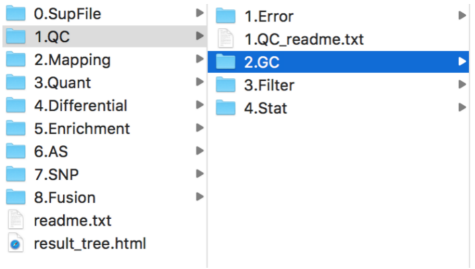

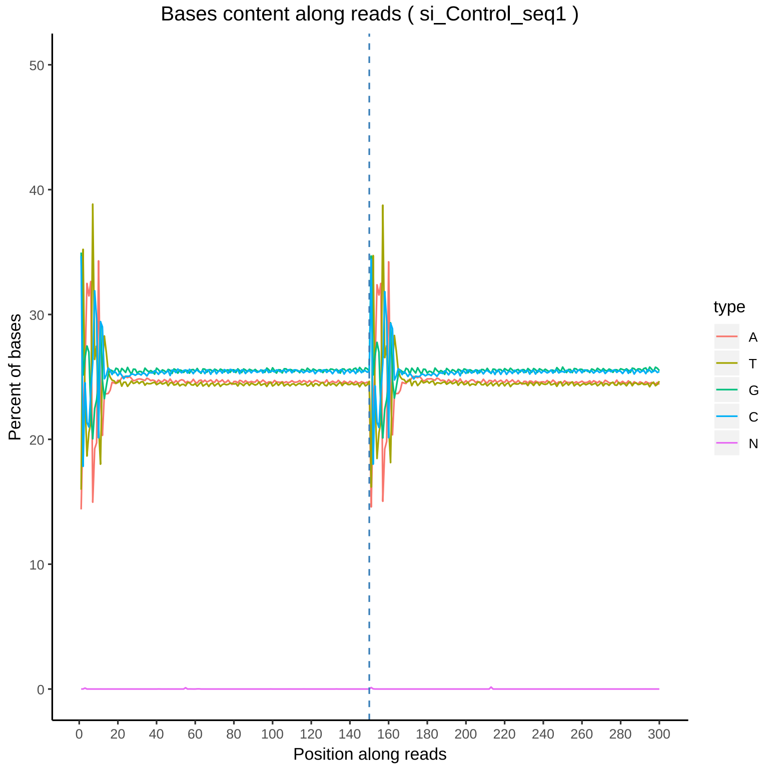

图3.3 GC含量分布

图中横坐标为reads的碱基位置，纵坐标为ATGCN五种碱基类型的百分比

3.1.4 测序数据过滤

测序获得的原始数据中包含少量带有测序接头或测序质量较低的reads，如下图所示。为了保证数据分析的质量及可靠性，需要对原始数据进行过滤，过滤内容如下。

- 去除带接头(adapter)的reads；
- 去除含N(N表示无法确定碱基信息)的reads；
- 去除低质量reads(Q_{phred}<=20的碱基数占整个read长度的50%以上的reads)。

每个样本的测序数据过滤情况，见结果文件：QC/3.Filter，如下图所示。

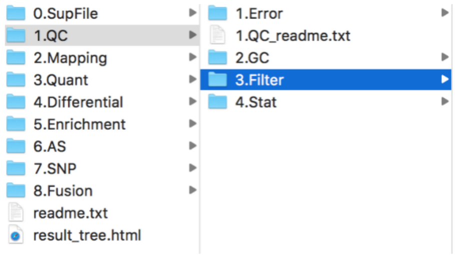

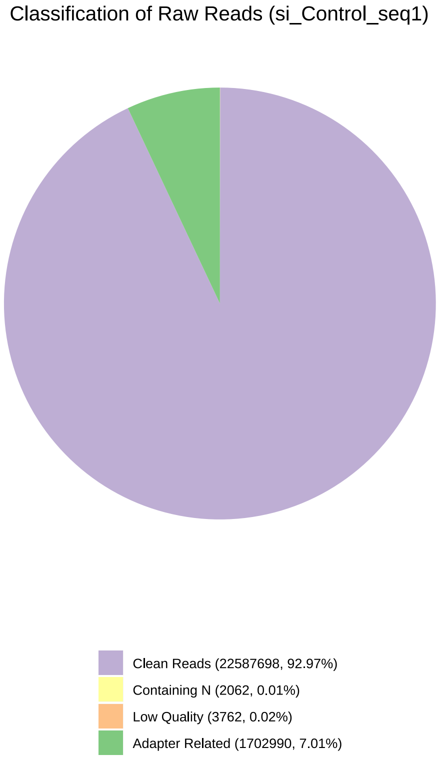

图3.4 样本测序数据过滤情况

注：图中不同颜色比例分别代表不同组分的比例

- **Adapter related**：带接头的reads所占比例。
- **Containing N**：带N碱基的reads所占比例。
- **Low quality**：测序质量低的reads所占比例。
- **Clean reads**：clean reads所占比例。

3.1.5 数据质量汇总

经过原始数据过滤、测序错误率检查、GC含量分布检查，获得后续分析使用的clean reads，本项目通过对XX样品进行测序，平均每个样品Clean data不少于 XX Gb。数据汇总如下表所示，见结果文件：QC/5.Stat/data_table.xls。

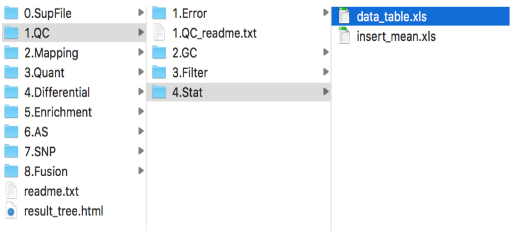

表3.2 样本测序数据质量汇总

sample	library	raw_reads	clean_reads	clean_bases	error_rate	Q20	Q30	GC_pct
si_NR5A2_seq1	FRAS202136674-1r	46859688	44426894	6.66G	0.02	98.77	96.48	50.02
si_Control_seq3	FRAS202136673-1r	45517758	43039674	6.46G	0.02	98.76	96.43	50.63
si_NR5A2_seq3	FRAS202136676-1r	45648882	44160512	6.62G	0.02	98.80	96.47	49.50
si_Control_seq2	FRAS202136672-1r	45255620	43780522	6.57G	0.02	98.70	96.24	50.49
si_NR5A2_seq2	FRAS202136675-1r	47110866	45759906	6.86G	0.02	98.76	96.36	49.91
si_FGD3_seq3	FRAS202136679-1r	47864816	46080938	6.91G	0.02	98.77	96.38	48.43
si_Control_seq1	FRAS202136671-1r	46884210	45175396	6.78G	0.02	98.69	96.19	50.92
si_FGD3_seq2	FRAS202136678-1r	45748696	44431706	6.66G	0.02	98.33	95.20	49.15
si_FGD3_seq1	FRAS202136677-1r	46053902	44654194	6.7G	0.02	98.69	96.15	49.18

注：我们对该表格设置了Copy/Print/Download以及Search功能，点击Copy可以复制表格中数据，用户可以任意新建excel或文本文档，可以将数据粘贴其中，并保留原有的行和列的格式；点击Print则可以打印表格中数据（链接打印机的情况下）；点击Download可以以CSV、Excel、PDF的三种格式下载表格中数据。在Search方框中输入指定信息，则会在下方表格中显示包含该信息的所有内容。以下表格中涉及的Copy/Print/Search功能与此处相同。

- **sample**：样品名称
- **library**：文库编号
- **raw_reads**：原始数据中的reads数
- **clean_reads**：原始数据过滤后的reads数
- **clean_bases**：原始数据过滤后的碱基数（clean base=clean reads*150bp）
- **error_rate**：数据整体测序错误率
- **Q20**：Phred数值大于20的碱基占总碱基的百分比
- **Q30**：Phred数值大于30的碱基占总碱基的百分比
- **GC_pct**：clean reads中G与C占四种碱基的百分比

Novogene 诺禾致源 | 提供领先的基因科技解决方案 Providing leading genomic services & solutions 北京诺禾致源科技股份有限公司

3.1.6 质控常见问题和解答

相关名词解释

- **Q20**：Phred数值大于20的碱基占总体碱基的百分比，其中Phred=-10log10(e)
- **Q30**：Phred数值大于30的碱基占总体碱基的百分比，其中Phred=-10log10(e)

测序错误率会随着测序长度的增加而升高，错误率在多少是可以接受的范围?

诺禾的测序会进行严格的数据质量把控。一般情况下，单个碱基位置的测序错误率应该低于1%，最高不超过6%。

3.2 参考基因组比对

测序片段（fragments）是mRNA随机打断的，为了确定这些一段由哪些基因转录来，需要将质控后的clean reads比对到参考基因组上。使用HISAT2 (http://ccb.jhu.edu/software/hisat2/faq.shtml)软件将Clean Reads与参考基因组进行快速精确的比对，获取Reads在参考基因组上的定位信息[4]。HISAT2软件官方手册 (https://ccb.jhu.edu/software/hisat2/manual.shtml#usage)。

如果参考基因组组装的较为完善，而且所测物种与参考基因组一致，且相关实验不存在污染，那么实验所产生的测序reads成功比对到基因组的比例会高于70% (Total Mapped Reads or Fragments)。本项目所用参考基因组为XX，下载地址：XX。基因组结构注释文件：XX。

Novogene 诺禾致源 | 提供领先的基因科技解决方案 Providing leading genomic services & solutions 北京诺禾致源科技股份有限公司

3.2.1 Reads与参考基因组比对情况统计

为了统计read1和read2各自的mapping率，下表中的total_reads数是read1和read2的总和，也就是上面数据质量汇总表格中clean_reads，真实reads数目应以数据质量汇总表格为准，样本与参考基因组比对情况见结果文件：QC/5.Stat/align_pct.xls。

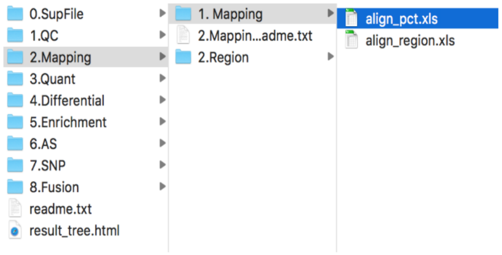

表3.3 样本与参考基因组比对情况统计

sample	total_reads	total_map	unique_map	multi_map	read1_map	read2_map	positive_map
si_NR5A2_seq2	45759906	44125128(96.43%)	42987578(93.94%)	1137550(2.49%)	21528321(47.05%)	21459257(46.9%)	21472575(46.92%)
si_FGD3_seq1	44654194	42963541(96.21%)	41871950(93.77%)	1091591(2.44%)	20978492(46.98%)	20893458(46.79%)	20920944(46.85%)
si_NR5A2_seq3	44160512	42587566(96.44%)	41506286(93.99%)	1081280(2.45%)	20778697(47.05%)	20727589(46.94%)	20735350(46.95%)
si_FGD3_seq3	46080938	44458528(96.48%)	43329713(94.03%)	1128815(2.45%)	21695331(47.08%)	21634382(46.95%)	21645851(46.97%)
si_Control_seq2	43780522	42206804(96.41%)	41143352(93.98%)	1063452(2.43%)	20606897(47.07%)	20536455(46.91%)	20548863(46.94%)
si_FGD3_seq2	44431706	42656816(96.01%)	41576380(93.57%)	1080436(2.43%)	20872880(46.98%)	20703500(46.6%)	20773849(46.75%)
si_Control_seq1	45175396	43404775(96.08%)	42243404(93.51%)	1161371(2.57%)	21158860(46.84%)	21084544(46.67%)	21094580(46.69%)
si_NR5A2_seq1	44426894	42651063(96.0%)	41509195(93.43%)	1141868(2.57%)	20778805(46.77%)	20730390(46.66%)	20735565(46.67%)
si_Control_seq3	43039674	41299380(95.96%)	40198989(93.4%)	1100391(2.56%)	20123847(46.76%)	20075142(46.64%)	20074731(46.64%)

- **sample**：样品名称
- **total_reads**：测序数据在质控后的clean reads数
- **total_map**：比对到基因组上的reads数及其百分比
- **unique_map**: 比对到参考基因组唯一位置的reads数及其百分比(用于后续定量数据分析reads)
- **multi_map**：比对到参考基因组多个位置的reads数及其百分比
- **read1_map**：比对到参考基因组的read1数及其百分比
- **read2_map**：比对到参考基因组的read2数及其百分比
- **positive_map**：比对到参考基因组正链上的reads数及其百分比
- **negative_map**：比对到参考基因组负链上的reads数及其百分比
- **splice_map**：拆分比对到基因组上的reads数及其百分比
- **unsplice_map**：未拆分比对到基因组上的reads数及其百分比
- **proper_map**：成对的read1和read2同时比对到基因组的reads数及其百分比

3.2.2 比对区域分布

根据比对结果，分别统计reads在基因组外显子区域，内含子区域以及基因间区所占的比例。一般模式物种的基因注释较为完善（如人和小鼠），其比对到外显子区域的比例很高。比对到内含子区域的reads可能来源于前体mRNA或可变剪接事件滞留的内含子。比对到基因间区的reads，可能来源于ncRNA或少许DNA片段污染，也可能是基因注释还不够完善。所有样本的测序reads在基因组区域分布情况如下图所示，见结果文件：QC/4.Region。

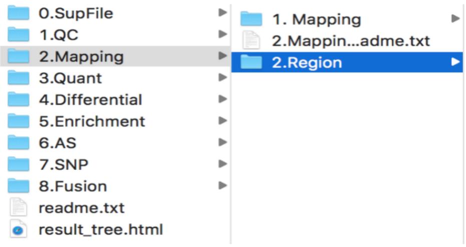

Percent of genome regions (si_Control_seq1)

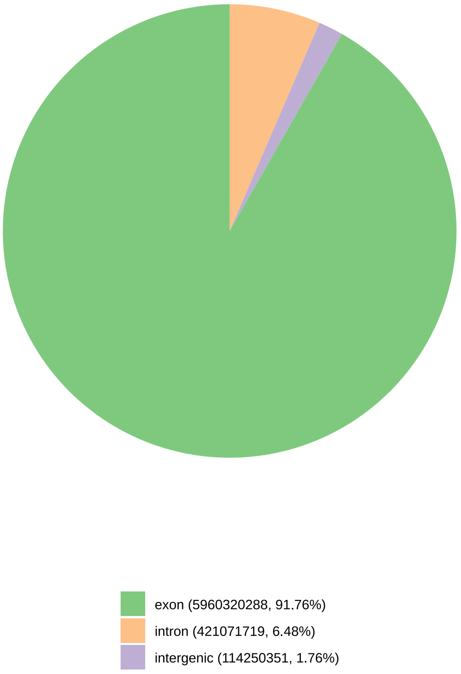

图3.5 测序reads在基因组区域分布情况

注：图中不同颜色比例分别代表比对到不同区域的reads比例

- **exon**：比对到基因组外显子区域的reads数及其占clean reads数的比例。
- **Intron**：比对到基因组内含子区域的reads数及其占clean reads数的比例。
- **Intergenic**：比对到基因间区的reads数及其占clean reads数的比例。

Novogene 诺禾致源 | 提供领先的基因科技解决方案 Providing leading genomic services & solutions 北京诺禾致源科技股份有限公司

3.2.3 比对可视化

Hisat2软件比对所得结果为sam格式文件，我们用samtools将它转为bam格式文件，bam文件是压缩的二进制文件，查看起来较为困难。客户可以结合物种参考基因组和注释文件使用IGV (Integrative Genomics Viewer) 浏览器对bam文件进行可视化浏览。IGV浏览器具有以下特点：(1)能在不同尺度下显示单个或多个读段在基因组上的位置，包括读段在各个染色体上的分布情况和在注释的外显子、内含子、剪接接合区、基因间区的分布情况等；(2)能在不同尺度下显示不同区域的读段丰度，以反映不同区域的转录水平；(3)能显示基因及其剪接异构体的注释信息；(4)能显示其他注释信息；(5)既可以从远程服务器端下载各种注释信息，又可以从本地加载注释信息。IGV浏览器使用方法可参考我们提供的使用说明文档IGVQuickStart (src/IGVQuickStart.pdf)

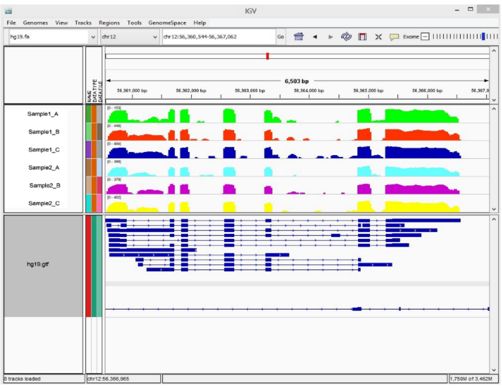

图3.6 IGV浏览器对比对结果的可视化展示

Novogene 诺禾致源 | 提供领先的基因科技解决方案 Providing leading genomic services & solutions 北京诺禾致源科技股份有限公司

3.2.4 比对常见问题和解答

RNA数据的比对软件和DNA数据的比对软件有何区别?

由于转录过程中存在可变剪接，测得的reads有很大一部分跨越不同外显子，所有RNA数据的比对软件要支持reads的splice比对。

造成mapping率较低的原因可能有哪些?

- 参考基因组组装不好
- 所测物种与参考基因组的亲缘关系较远
- 样品的特殊处理或外源污染

mapping时用的是read全长，还是头尾有处理?

实验方面，我们使用标准的RNA-seq试剂盒，其index处于Adapter中间，在测序中由Index read完成，由此测序得到的read1和read2的各个碱基全都是样本的序列，因此mapping时，头尾可以不用处理。即便read被测穿而带有接头或含有低质量的碱基，我们也是将整条read去掉。

3.3 定量分析

根据基因比对在参考基因组上的位置信息，从而统计每个基因（包括新预测基因）从起始到终止范围内覆盖的reads数。分别过滤掉比对质量值低于10的reads，非成对比对上的reads，比对到基因组多个区域的reads。该部分分析采用subread软件中的featureCounts工具[6]。本报告中仅对表格中的部分部分进行展示，详细信息见结果文件。

Novogene 诺禾致源 | 提供领先的基因科技解决方案 Providing leading genomic services & solutions 北京诺禾致源科技股份有限公司

3.3.1 基因表达定量

我们对每个样本分别进行基因表达水平的定量分析，再合并得到所有样本的表达矩阵[7]，第一列为基因的ID，其余列为各样本的原始read count值，如下表所示，见结果文件：Quant/gene_count.xls。

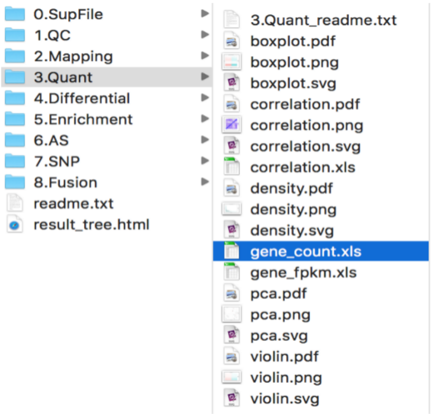

表3.4 基因表达定量结果展示

gene_id	si_NR5A2_seq2	si_FGD3_seq1	si_NR5A2_seq3	si_FGD3_seq3	si_Control_seq2	si_FGD3_seq2	si_Control_seq1
ENSG00000198804	303870	272685	287493	308606	360505	253776	418008
ENSG00000198712	151349	142286	145888	165290	155156	144233	175255
ENSG00000198886	132602	120937	122214	120050	151869	124765	175217
ENSG00000210082	132298	108364	114649	135977	118784	113036	149169
ENSG00000198938	114894	94848	107750	108183	123600	95344	144078
ENSG00000156508	98154	106653	95073	118173	84126	104475	91406
ENSG00000074800	82144	75210	74077	79616	85473	74712	96044
ENSG00000198899	70942	58826	61041	61537	87194	62317	105768

- **gene_id**：基因编号
- **sample**：各样本定量所得的原始read count值
- **gene_name**：基因名称
- **gene_chr**：基因所在的染色体名称
- **gene_start**：基因所在染色体的起始位置
- **gene_end**：基因所在染色体的终止位置
- **gene_strand**：基因所在染色体的正负链信息
- **gene_length**：基因长度，基因起始到终止所有exon非重叠区域的总和
- **gene_biotype**：基因类型，如编码蛋白基因，长链非编码基因等
- **gene_description**：基因功能描述
- **gene_tf_family**：基因转录因子家族注释

3.3.2 基因表达分布

由于测序深度和基因长度的影响，RNA-seq的基因表达值一般不用read count来表示，而是用FPKM，FPKM先后对测序深度和基因长度进行了校正[8]。我们计算各样本所有基因的表达值(FPKM)后，通过盒形图展示不同样本基因表达水平的分布情况，如下图所示。图中横坐标为样品名称，纵坐标为log2(FPKM+1)，每个区域的盒形图对五个统计量(至上而下分别为最大值，上四分位数，中位数，下四分位数和最小值)，见结果文件：Quant/boxplot.svg。

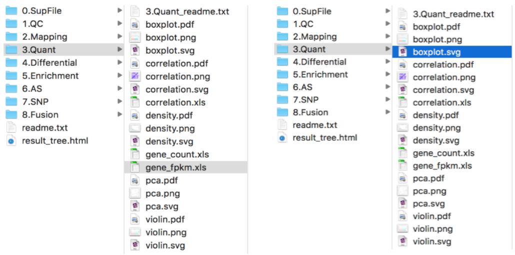

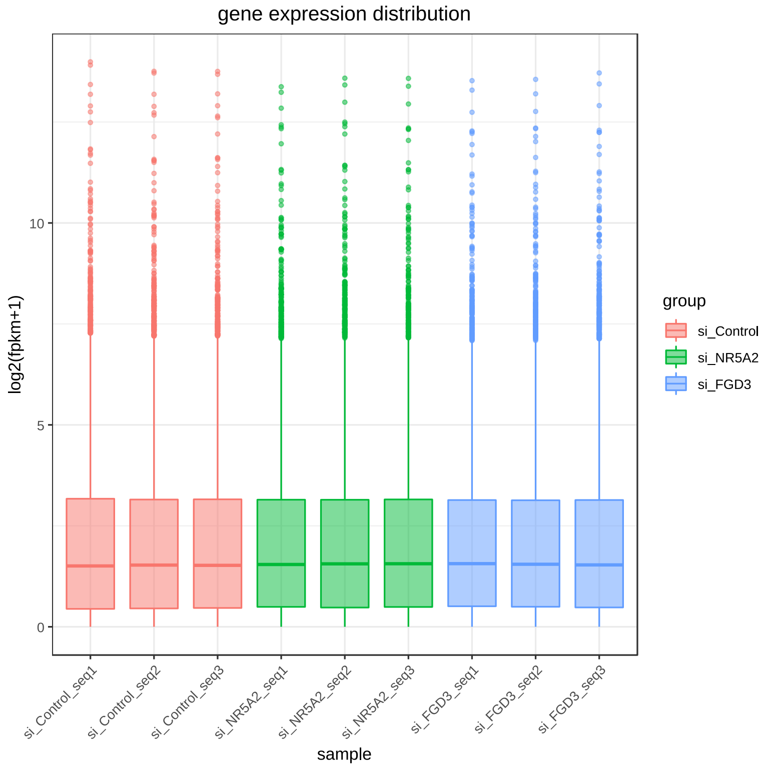

图3.7 样本基因表达量分布盒形图

图中横坐标为样品名称，纵坐标为log2(FPKM+1)

3.3.3 样本间相关性

生物学重复通常是任何生物学实验所必须的，目前主流期刊也基本要求生物学重复。生物学重复主要有两个用途：一个是证明所涉及的生物学实验操作不是偶然，而是可重复的。另一个是为了确保后续的差异基因分析得到更可靠的结果。样品间基因表达水平相关性是检验实验可靠性和样本选择是否合理的重要指标。相关系数越接近1，表明样品之间表达模式的相似度越高。Encode计划建议皮尔逊相关系数的平方(R^2)大于0.92(理想的取样和实验条件下)。具体的项目操作中，我们要求生物学重复样品间R^2至少要大于0.8，否则需要对样品做出合适的解释，或者重新进行实验。根据各样本所有基因的FPKM值计算组内及组间样本的相关性系数，绘制成热图，可直观显示组间样本差异及组内样本重复情况。样本间相关性系数越高，其表达模式越为接近，样本相关性热图如下图所示，见结果文件：Quant/correlation.svg。

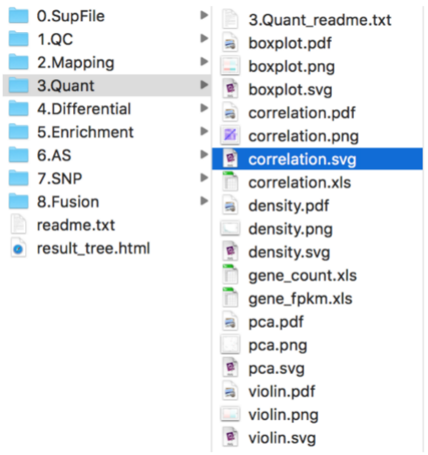

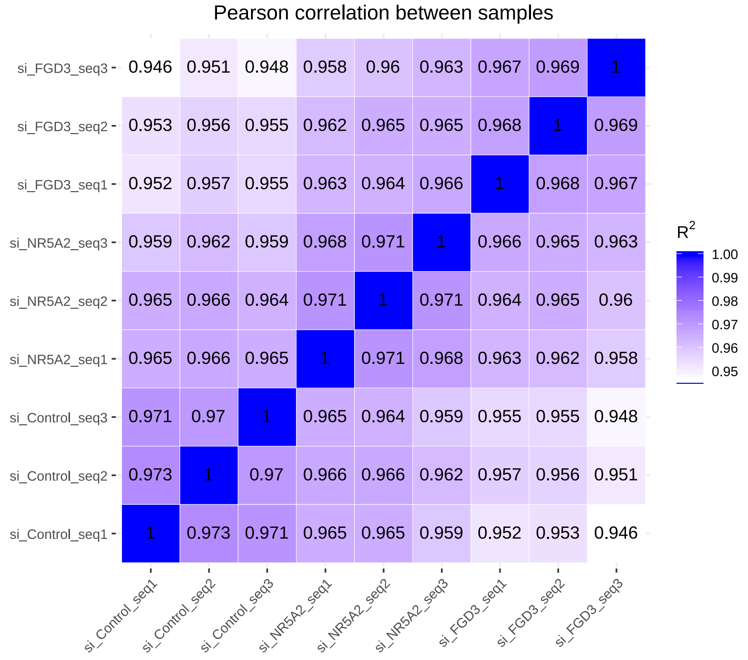

图3.8 样本间相关性热图

图中横纵坐标为各样本相关系数的平方

Novogene 诺禾致源 | 提供领先的基因科技解决方案 Providing leading genomic services & solutions 北京诺禾致源科技股份有限公司

3.3.4 主成分分析

主成分分析（PCA）也常用来评估组间差异及组内样本重复情况，PCA采用线性代数的计算方法，对数以万计的基因变量进行降维及主成分提取。我们对所有样本的基因表达值（FPKM）进行PCA分析，如下图所示。理想条件下，PCA图中，组间样本应该分散，组内样本应该聚在一起，见结果文件：Quant/pca.svg。

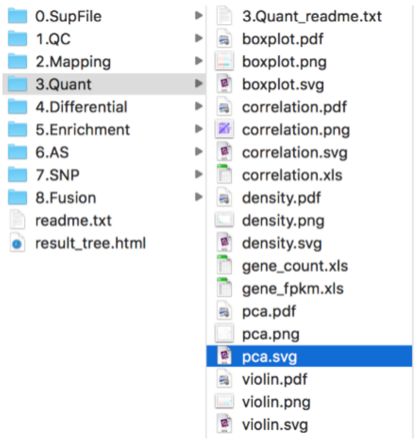

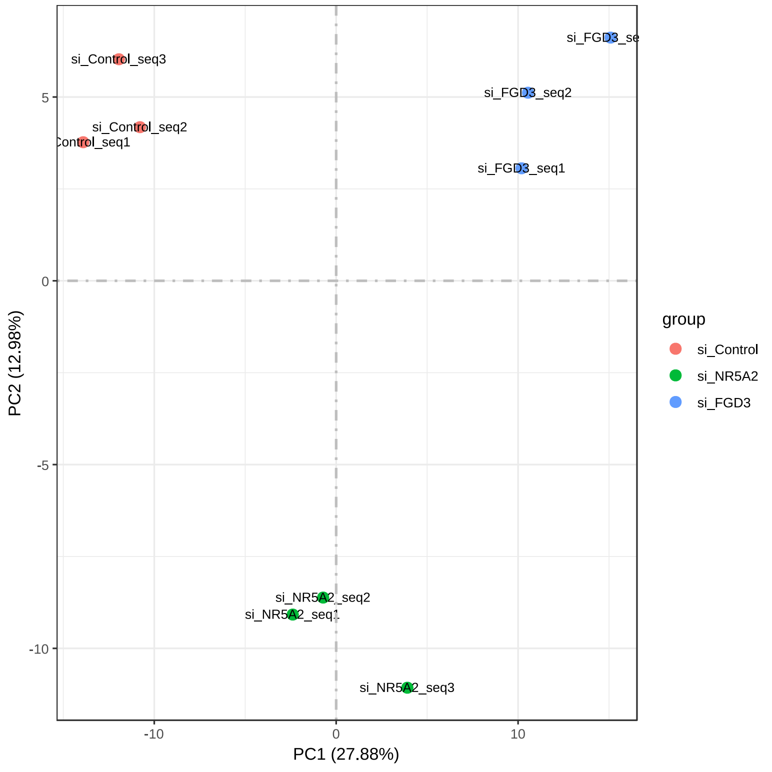

图3.9 主成分分析结果图

图中横坐标为第一主成分，纵坐标为第二主成分

3.3.5 定量分析常见问题及解答

定量过程中，比对到基因组多个位置的reads（multi mapped）是如何处理的?

这些reads比对到基因组多个位置，无法确定是由哪个基因转录而来，所以这部分reads在定量过程中直接被过滤掉。

有重叠区域的两个基因，重叠区域的reads在定量时如何分配?

基因重叠区域的reads也无法确定由哪个基因转录而来，这部分的reads也是被过滤处理。

定量分析表格中，基因的转录因子家族注释是如何进行的?

一方面通过转录因子数据库（AnimalTFDB/PlantTFDB）进行注释（针对数据库已收录的物种），一方面通过Pfam/SUPERFAMILY等蛋白结构域数据库进行预测

FPKM是如何计算的?

FPKM(expected number of Fragments Per Kilobase of transcript sequence per Millions base pairs sequenced)是指每百万fragments中来自某一基因每千碱基长度的fragments——成对的reads数目，其同时考虑了测序深度和基因长度对fragments计数的影响，是目前常用的基因表达水平估算方法(Trapnell, Cole, et al., 2010)。

基因表达的阈值是多少？为什么设置为这个阈值?

一般认为FPKM大于1时基因表达，这个阈值是主流杂志推荐的。

样品间的相关性有何意义？如何计算?

样品间的相关性反应了样品间基因表达模式的相似程度，在一定程度上可反应生物学重复的好坏。相关系数越接近1，样品间的相似度越高。理论上，生物学重复间的样品的相关系数应大于生物学重复外的样品的相关系数。相关系数的计算方法常见的有三种：A. Pearson correlation; B. Spearman rank correlation; C. Kendall’s τ。我们使用Pearson相关系数进行计算。

主成分分析是什么?

主成分分析(Principal Component Analysis, PCA) 是一种多元降维分析方法。PCA的核心思想在于，在尽可能保留数据的差异的前题下，降低数据的维度，也就是抽象出更少的互不相关的变量来描述各数据。数据集是一群在多维空间中的点，在保持这一群点的相对空间位置不变的情况下，旋转到一个新的坐标系（坐标轴就是各PC），使得各点在新的坐标轴上的坐标（投影）的方差最大，而投影方差最大的坐标轴即为PC1，其次为PC2。

3.4 差异分析

基因表达定量完成后，需要对其表达数据进行统计学分析，筛选样本在不同状态下表达水平显著差异的基因。差异分析主要分为三个步骤。

- 首先对原始的readcount进行标准化（normalization），主要是对测序深度的校正。
- 然后统计学模型进行假设检验概率（pvalue）的计算
- 最后进行多重假设检验校正，得到FDR值（错误发现率，padj是其常见形式）[10-12]。

针对不同的实验情况，我们选用合适的软件进行基因表达差异显著性分析，具体如下表所示。

表3.5 表达差异分析所用软件及差异基因筛选标准

类型	软件	标准化方法	pvalue计算模型	FDR计算方法	差异基因筛选标准
有生物学重复	DESeq2(Anders et al, 2014)	DESeq	负二项分布	BH	$\lvert log2(FoldChange) \rvert > 0$ & padj < 0.05
无生物学重复	edgeR(Robinson et al, 2010)	TMM	负二项分布	BH	$\lvert log2(FoldChange) \rvert > 1$ & padj < 0.05

若按照以上标准筛选得到的差异基因过少（低于100），很有可能导致后面的功能富集分析没有显著性结果，所以，我们会根据项目的具体情况，适当地降低筛选差异基因的阈值标准。若项目实验只关注某几个基因的表达情况（如基因敲除），不在意富集结果，从下面的差异分析表格中筛选关注的那几个基因即可。

一般来说，如果一个基因在两组样品中的表达量差异达到两倍以上，我们认为这样的基因是具有表达差异的。为了判断两个样品之间的表达量差异究竟是由于各种误差导致的还是本质差异，我们需要对所有基因在这两个样本中的表达量数据进行假设检验。而转录组分析是针对成千上万个基因进行的，这样会导致假阳性的累积，基因数目越多，假设检验的假阳性累积程度会越高，所以引入padj对假设检验的P-value进行校正，从而控制假阳性的比例[13]。

差异基因的筛选标准是非常重要的，我们给出的标准|log2(FoldChange)| > 1 & padj< 0.05是常用的经验值，在实际项目中可以根据情况灵活选择。例如，差异倍数可以选择1.5倍，也可以选择3倍，padj常用的阈值包括0.01、0.05、0.1等。若按照以上标准筛选得到的差异基因过少，很有可能导致后面的功能富集分析没有显著性结果。若项目实验只关注某几个基因的表达情况（如基因敲除），不在意富集结果，从下面的差异分析表格中筛选关注的那几个基因即可。反之，如果得到的差异基因数目过多，不利于后续目标基因的筛选，这个时候可使用更严格的阈值标准进行筛选，则可以使用更严格的阈值标准进行筛选。

3.4.1 差异基因列表

每个比较组合的差异显著性分析如下表所示，表格展示的是第一个比较组合的所有基因的差异显著性结果的前30行，见结果文件：Differential/1.deglist。

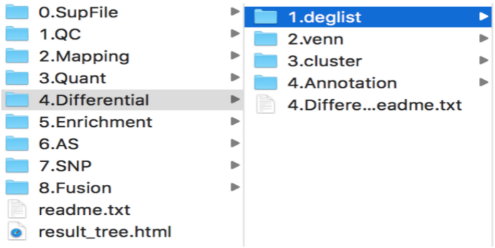

表3.6 差异基因列表部分结果展示

gene_id	si_NR5A2_seq1	si_NR5A2_seq2	si_NR5A2_seq3	si_Control_seq1	si_Control_seq2	si_Control_seq3	si_NR5A2
ENSG00000105323	6348.384	6432.576	6223.5433	12194.793	10981.395	11291.317	6334.8342
ENSG00000077942	10633.154	10797.945	10460.5581	20757.312	18536.853	18684.095	10630.5523
ENSG00000124762	6470.917	6581.795	6267.5363	11501.861	10704.408	10684.346	6440.0826
ENSG00000167460	10366.693	10884.435	11150.7594	6667.686	6462.677	6421.001	10800.6291
ENSG00000130513	823.695	741.343	644.2531	1899.943	1849.945	1858.328	736.4303
ENSG00000142669	1223.386	1310.656	1192.6991	2918.901	2536.345	2610.805	1242.2473
ENSG00000124466	2046.109	2020.635	1927.8711	3537.226	3280.367	3432.918	1998.2049
ENSG00000108518	8133.866	8168.079	7743.7459	12637.330	11866.942	11823.456	8015.2304

- **gene_id**：基因编号
- **sample**：各样本标准化后的readcount值
- **group**：各组readcount均值
- **log2FoldChange**：处理组与对照组基因表达水平的比值，再经过差异分析软件收缩模型处理，最后以2为底取对数
- **pvalue**：显著性检验的值
- **padj**：多重假设检验校正后的p值
- **gene_name**：基因名称
- **gene_chr**：基因所在的染色体名称
- **gene_start**：基因在染色体的起始位置
- **gene_end**：基因在染色体的终止位置
- **gene_strand**：基因所在染色体的正负链信息
- **gene_length**：基因长度，基因起始到终止所有exon非重叠区域的总和
- **gene_biotype**：基因类型，如编码蛋白基因，长链非编码基因等
- **gene_description**：基因功能描述
- **gene_tf_family**：基因转录因子家族注释

每个基因在所有比较组合的差异显著性分析如下表所示，见结果文件：Differential/1.deglist/all_compare.xls。

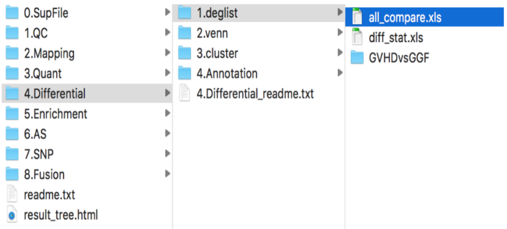

表3.9 每个基因在所有比较组合的差异显著性分析部分结果展示

表3.7 每个基因在所有比较组合的差异显著性分析部分结果 展示

gene_id	si_NR5A2_seq2_count	si_FGD3_seq1_count	si_NR5A2_seq3_count	si_FGD3_seq3_count	si_Control_seq2_count	si_FGD3_seq2_count
ENSG00000198804	303870	272685	287493	308606	360505	253776
ENSG00000198712	151349	142286	145888	165290	155156	144233
ENSG00000198886	132602	120937	122214	120050	151869	124765
ENSG00000210082	132298	108364	114649	135977	118784	113036
ENSG00000198938	114894	94848	107750	108183	123600	95344
ENSG00000156508	98154	106653	95073	118173	84126	104475
ENSG00000074800	82144	75210	74077	79616	85473	74712
ENSG00000198899	70942	58826	61041	61537	87194	62317

- **gene_id**：基因编号
- **sample_count**：各样本的原始readcount值
- **sample_fpkm**：各样本的fpkm值
- **compare_treat**：某个比较组合处理组标准化readcount的均值
- **compare_control**：某个比较组合对照组标准化readcount的均值
- **compare_log2FoldChange**：某个比较组合处理组与对照组基因表达水平的比值，再以2为底取对数
- **compare_pvalue**：某个比较组合显著性检验的p值
- **compare_padj**：某个比较组合多重假设检验校正后的p值
- **gene_name**：基因名称

- **gene_chr**：基因所在的染色体名称
- **gene_start**：基因在染色体的起始位置
- **gene_end**：基因在染色体的终止位置
- **gene_strand**：基因所在染色体的正负链信息
- **gene_length**：基因长度，基因起始到终止所有exon非重叠区域的总和
- **gene_biotype**：基因类型，如编码蛋白基因，长链非编码基因等
- **gene_description**：基因功能描述
- **gene_tf_family**：基因转录因子家族注释

3.4.2 差异基因统计

每个比较组合的差异基因（包括上调和下调）数目统计以及筛选差异的标准如下表所示，见结果文件：Differential/1.deglist/diff_stat.xls。

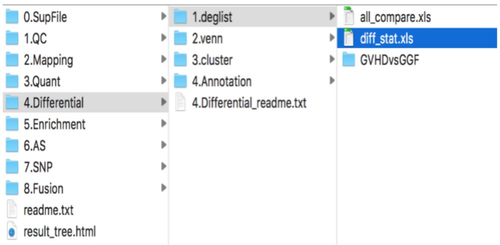

表3.8 差异基因统计结果展示

compare	all	up	down	threshold
si_NR5A2vssi_Control	3515	1450	2065	DESeq2 padj<0.05 |log2FoldChange|>0.0
si_FGD3vssi_Control	5861	2569	3292	DESeq2 padj<0.05 |log2FoldChange|>0.0

- **compare**：比较组合名称
- **all**：该比较组合差异基因总数
- **up**：该比较组合差异基因上调的数目
- **down**：该比较组合差异基因下调的数目
- **threshold**：该比较组合进行差异基因筛选的软件及阈值

对每个比较组合的差异基因数（包含上调和下调）用柱状图进行展示：

diff_stat

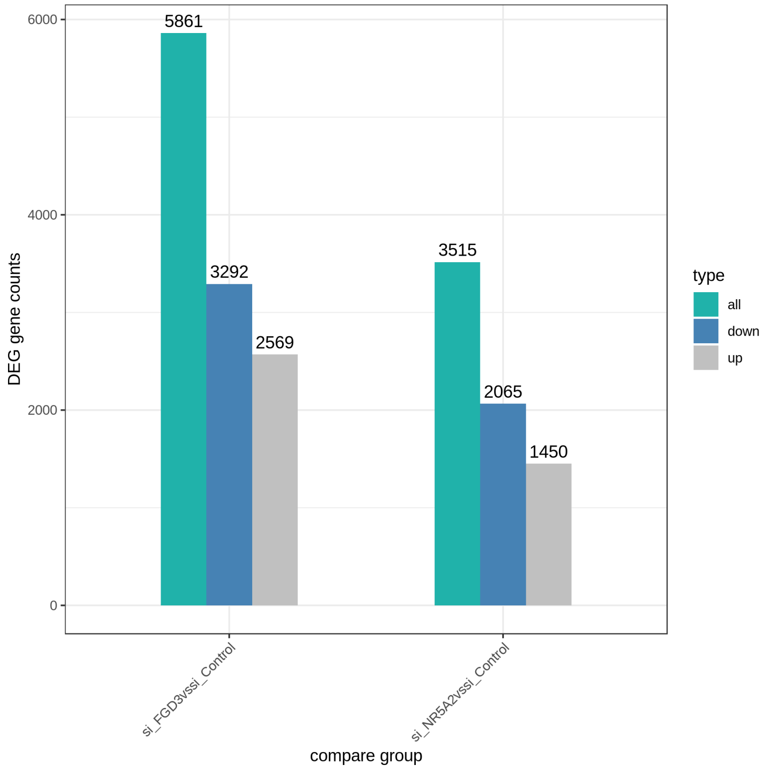

图3.10 差异比较组合差异基因数目统计柱状图

注：蓝色和灰色分别表示上调和下调的差异基因，柱子上的数字表示差异基因数目

火山图可直观展示每个比较组合的差异基因分布情况，如下图所示。图中横坐标表示基因在处理和对照两组中的表达倍数变化(log2FoldChange)，纵坐标表示基因在处理和对照两组中表达差异的显著性水平(-log10padj或-log10pvalue)。为上调基因用红色点表示，下调基因用绿色点表示，见结果文件：Differential/1.deglist/{比较组合}/_volcano.png。

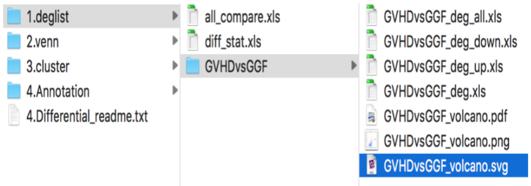

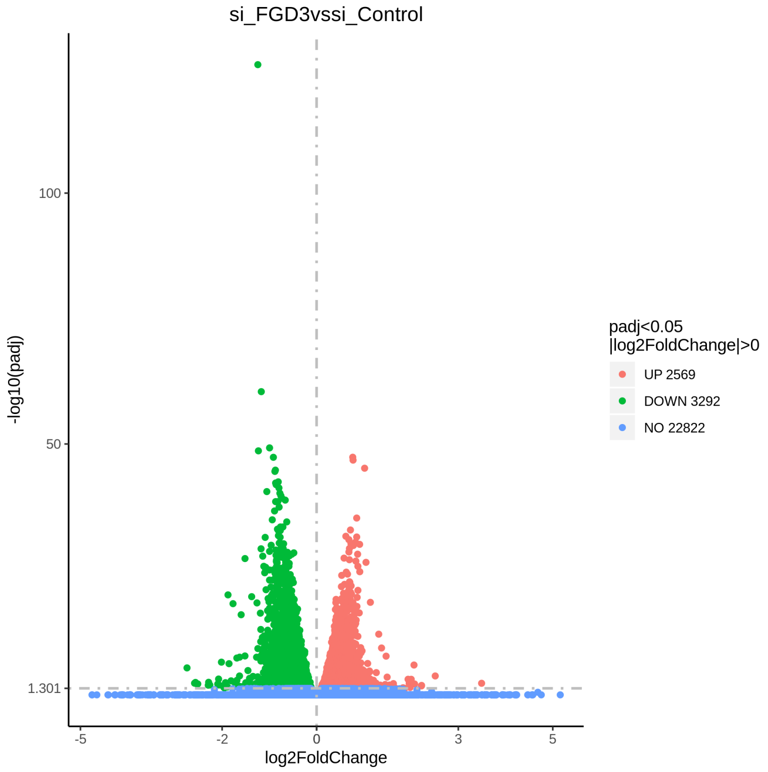

图3.11 差异基因火山图

图中横坐标为log2FoldChange值，纵坐标为-log10padj或-log10pvalue，蓝色的虚线表示差异基因筛选标准的阈值线

3.4.3 差异基因韦恩图

韦恩图可展示不同比较组合间差异基因的重叠情况，通过韦恩图，可筛选某几个比较组合共有或独有的差异基因。若只有一个差异比较组合，我们默认绘制该比较组合处理组与对照组的共表达基因韦恩图。韦恩图中圈内所有数字之和代表该比较组合差异基因总数，重叠区域表示组合间共有的差异基因个数，具体如下图所示，见结果文件：Differential/2.venn。

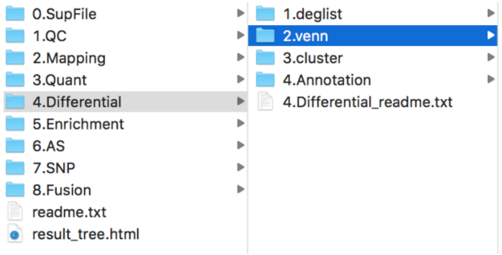

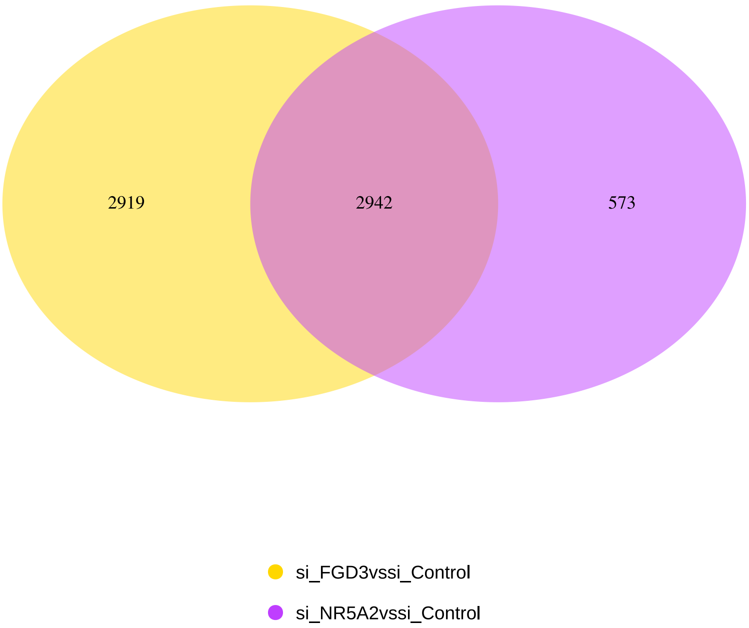

图3.12 差异基因韦恩图

注：不同的颜色表示不同的比较组合

3.4.4 差异基因聚类

将所有比较组的差异基因取并集之后作为差异基因集。两组以上的实验，可对差异基因集进行聚类分析，将表达模式相近的基因聚在一起。我们采用主流的层次聚类对基因的FPKM值进行聚类分析，对行（row）进行均一化处理（Z-score）。热图中表达模式相近的基因或样本会被聚集在一起，每个方格中的颜色反映的不是基因表达值，而是表达数据的行进行均一化处理后得到的数值（一般在-2到2之间），所以热图中的颜色只能横向比较（同一基因在不同样本中的表达情况），不能纵向比较（同一样本不同基因的表达情况）。结果文件中既有组间的聚类，也有样品间的聚类。结题报告展示了样品间的聚类，具体如下图所示，见结果文件：Differential/3.cluster/cluster1/cluster1_heatmap.png。

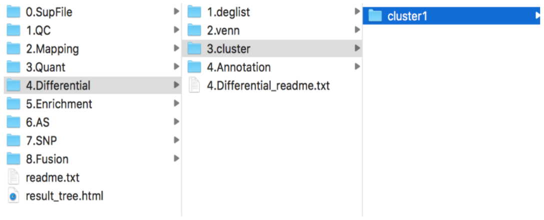

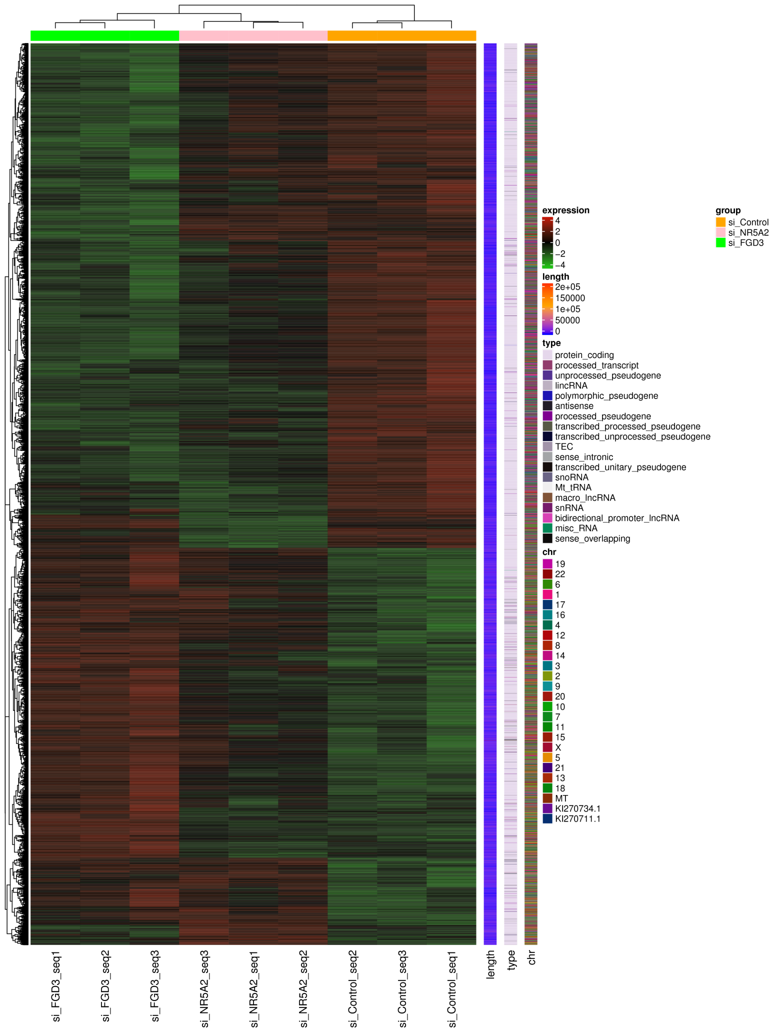

图3.12 差异表达基因聚类热图

图中横坐标为样品名，纵坐标为差异基因FPKM归一化后的数值，颜色越红，表达量越高，越绿，表达量越低。热图中还添加了每个基因所属的染色体，基因长度以及基因的生物学类型

Novogene 诺禾致源 | 提供领先的基因科技解决方案 Providing leading genomic services & solutions 北京诺禾致源科技股份有限公司

3.4.5 差异分析常见问题及解答

能否用FPKM进行差异分析?

大部分差异分析软件（DESeq，DESeq2和edgeR）用原始的read count作为输入文件，使用负二项分布模型来估算样本间基因差异表达的概率。这些软件自身对会read count做一些校正（主要是测序深度），而FPKM是校正后的表达值，所以用FPKM做差异分析相当于做了两次校正，是不合理的。

为什么要做多重假设检验计算padj值，而不是直接使用pvalue来筛选差异基因?

单次假设检验使用pvalue没有问题，但在差异分析过程中，我们要对每个基因进行一次假设检验，一个物种往往有几万个基因，也就要做几万次假设检验，那样假阳性就大大增加。假设pvalue为0.05（一百个差异基因只有五个是假阳性），对一个被做假设检验的基因来说，这种准确性是够了，但对于整体的几万个基因，准确性就远远不够了，因为每检验一万个基因，就会有500个是假阳性。为了合理控制，有必要引入一个更加严格的指标，也就是校正后的pvalue。当然，若是差异基因过少，也可以退而求其次使用pvalue，有生物学意义并能通过实验验证即可。

差异基因筛选条件最大能设的阈值是多少？适当降低阈值标准可以吗?

一般来说，等级较高的文章阈值的设置会比较严格；而在某些文章中，差异基因筛选阈值会适当放宽：如在一些无生物学重复的文章中，只将padj作为差异基因筛选标准，不考虑log2foldchange；有的文章则将pvalue作为差异基因的筛选标准。

如何对差异基因进行实验验证呢?

可参考我们整理的实验验证 (src/experimental_verification.pdf)文档。

聚类分析有哪些步骤?

先对所有样本的基因表达值（FPKM表达矩阵）进行对数转换，使数据近似于理想的正态分布（聚类等分析技术依赖于一个提前假设：数据要服从理想化的统计分布，通常是正态分布），再对转换后的所有数据点进行距离计算（欧式距离），最后通过层次聚类中的complete聚类方法，将N个对象分类到k个类群，每个类群中的对象彼此相似。层次聚类算法主要分为三个步骤，如下所示。

- 初始时刻，所有点本身是一个聚类
- 找到距离最近的两个聚类（刚开始也就是两个点），形成一个聚类，两个聚类的距离指的是聚类中最近的两个点之间的距离
- 重复第二步，直到所有点都被聚类到聚类中

聚类分析中均一化是如何计算的?

表达矩阵每行数据的各个数值减去每行数据的均值，再除以每行数据的标准差，均一化处理后的数值范围一般在[-2,2]区间内。

转录因子如何预测?

动物转录因子鉴定使用动物转录因子数据库-animalTFDB 3.0；植物转录因子鉴定使用植物转录因子数据库-PlantTFDB 4.0。对于数据库中有收录的物种，如果是Ensembl geneid则直接筛选转录因子；非Ensembl geneid的基因，通过interproscan软件进行SUPERFAMILY和Pfam注释，得到每个基因的SUPERFAMILY和Pfam的ID，然后利用DBD（Transcription factor prediction database）数据库中已经注释好的每个SUPERFAMILY和Pfam对应的转录因子家族的信息进行预测。真菌转录因子预测通过interproscan软件进行SUPERFAMILY和Pfam注释，得到每个基因的SUPERFAMILY和Pfam的ID，然后利用DBD（Transcription factor prediction database）数据库中已经注释好的每个SUPERFAMILY和Pfam对应的转录因子家族的信息进行预测。

Novogene 诺禾致源 | 提供领先的基因科技解决方案 Providing leading genomic services & solutions 北京诺禾致源科技股份有限公司

3.5 富集分析

我们根据基因表达量分析得到差异基因之后，必须进一步落到基因的功能上来。对于转录组分析而言，往往涉及到成千上万个基因，这会使分析变得很复杂。解决思路是将一个基因列表分成多个部分，从而减少分析的复杂度。为了解决怎么分成不同类，通常会对基因功能进行富集分析, 期望发现在生物学过程中起关键作用的生物通路, 从而揭示和理解生物学过程的基本分子机制。功能富集分析可以将成百上千个基因、蛋白或者其他分子分到不同的通路中，以减少分析的复杂度。另外，在两种不同实验条件下，激活的通路显然比简单的基因或蛋白列表更有说服力。基因功能富集分析首先要构建基因集（gene set，如GO和KEGG数据库等），也就是基因组注释信息进行分类。然后再把我们的目标基因集（差异基因集或者其他基因集）映射到背景基因集上，注意区分注释与富集。

我们采用clusterProfiler软件对差异基因集进行GO功能富集分析，KEGG通路富集分析等。富集分析基于超几何分布原理，其中差异基因集为差异显著分析所得差异基因并注释到GO或KEGG数据库的基因集，背景基因集为所有进行差异显著分析的基因并注释到GO或KEGG数据库的基因集。富集分析结果是对每个差异比较组合的所有差异基因集、上调差异基因集、下调差异基因集进行富集。本报告中展示的表格是选取某一个比较组合的富集分析结果，图片是所有组合的富集分析结果。

$$P = 1 - \sum_{i=0}^{m-1} \frac{\binom{M}{i}\binom{N-M}{n-i}}{\binom{N}{n}}$$

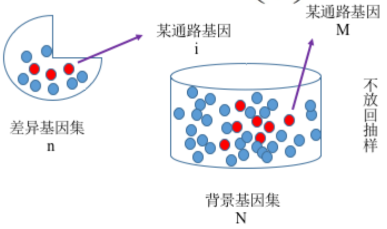

图3.13 基因富集分析原理图

Novogene 诺禾致源 | 提供领先的基因科技解决方案 Providing leading genomic services & solutions 北京诺禾致源科技股份有限公司

3.5.1 GO功能富集分析

GO(Gene Ontology)是描述基因功能的综合性数据库，可分为生物过程（biological process）和细胞组成（cellular component）分子功能（Molecular Function）三个部分。GO功能富集以padj小于0.05作为为显著性富集的阈值，富集结果如下表所示，见结果文件：Enrichment/GO。

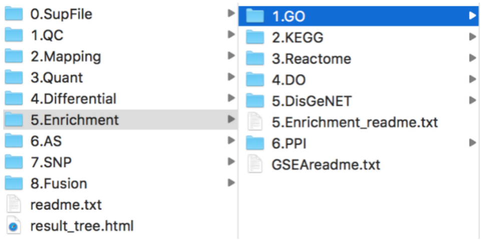

表3.9 差异基因GO富集分析结果部分展示

Category	GOID	Description	GeneRatio	BgRatio	pvalue
BP	GO:0008380 (GO:0008380)	RNA splicing	152/3192	412/13834	0
BP	GO:0006397 (GO:0006397)	mRNA processing	166/3192	465/13834	0
BP	GO:0043312 (GO:0043312)	neutrophil degranulation	147/3192	405/13834	0
BP	GO:0036230 (GO:0036230)	granulocyte activation	150/3192	417/13834	0
BP	GO:0002283 (GO:0002283)	neutrophil activation involved in immune response	147/3192	407/13834	0
BP	GO:0042119 (GO:0042119)	neutrophil activation	148/3192	413/13834	0
BP	GO:0002446 (GO:0002446)	neutrophil mediated immunity	148/3192	417/13834	0
BP	GO:0000375 (GO:0000375)	RNA splicing, via transesterification reactions	119/3192	319/13834	0

- **Category**：GO数据库分类，包括生物过程BP、细胞组分CC、分子功能MF
- **GOID**：GO编号
- **Description**：GO编号对应的功能描述
- **GeneRatio**：注释到GO编号上的差异基因数与差异基因总数的比值
- **BgRatio**：注释到GO编号上的背景基因数与背景基因总数的比值
- **pvalue**：显著性检验p值
- **padj**：多重假设检验校正后的p值
- **geneID**：注释到GO编号上的差异基因ID
- **geneName**：注释到GO编号上的差异基因名称
- **Count**：注释到GO编号上的差异基因数
- **Up**: 与该Term相关的上调的差异基因个数
- **Up_Gene_id**: 与该Term相关的上调的差异基因ID
- **Down**: 与该Term相关的下调的差异基因个数
- **Down_Gene_id**: 与该Term相关的下调的差异基因ID

从GO富集分析结果中，选取最显著的30个Term绘制柱状图进行展示，若不足30个，则绘制所有Term，如下图所示。图中横坐标为GO Term，纵坐标为GO Term富集的显著性水平，数值越高越显著，不同颜色分别代表BP，CC，MF三个GO子类。见结果文件：Enrichment/GO。

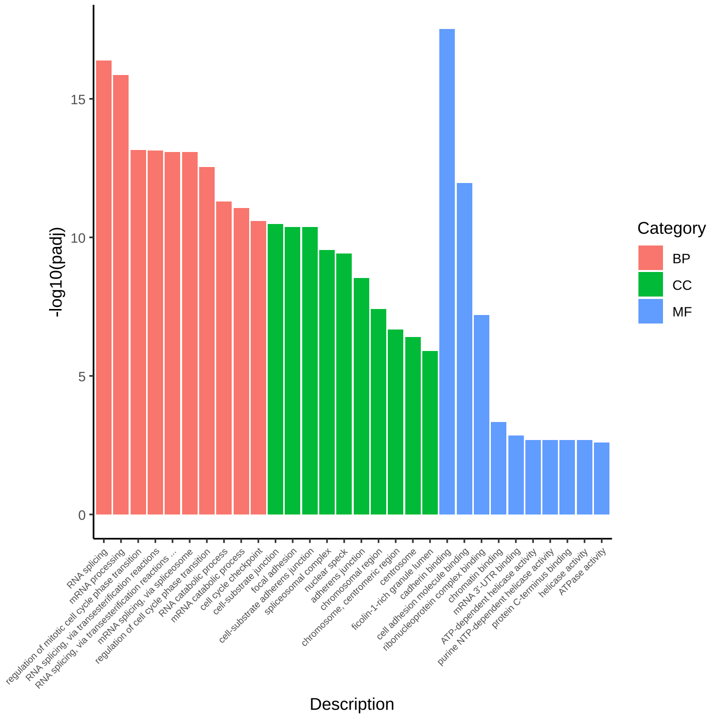

图3.14 GO富集分析柱状图

注：图中横坐标为GO Term，纵坐标为GO Term富集的显著性水平，用-log10(padj)表示，不同的颜色分别表示不同的功能分类

从GO富集分析结果中，选取最显著的30个Term绘制柱状图进行展示，若不足30个，则绘制所有Term，按生物过程、细胞组分和分子功能三大类别及差异基因上下调分类画的柱状图。

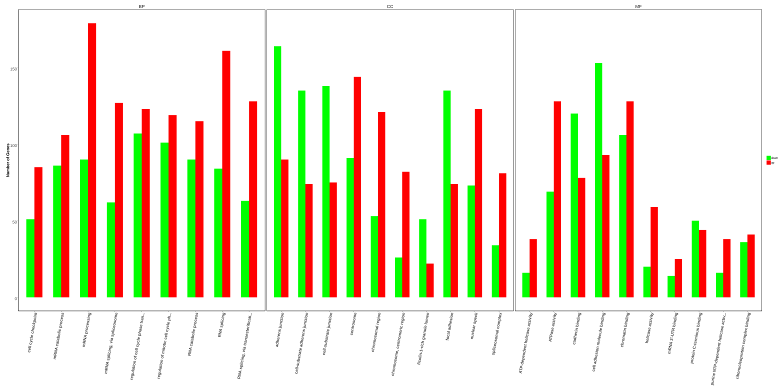

图中横坐标为GO Term，纵坐标为GO Term富集的显著性水平

从GO富集分析结果中，选取最显著的30个Term绘制散点图进行展示，若不足30个，则绘制所有Term，如下图所示。图中横坐标为注释到GO Term上的差异基因数与差异基因总数的比值，纵坐标为GO Term，点的大小代表注释到GO Term上的基因数，颜色从红到紫代表富集的显著性大小，见结果文件：Enrichment/GO。

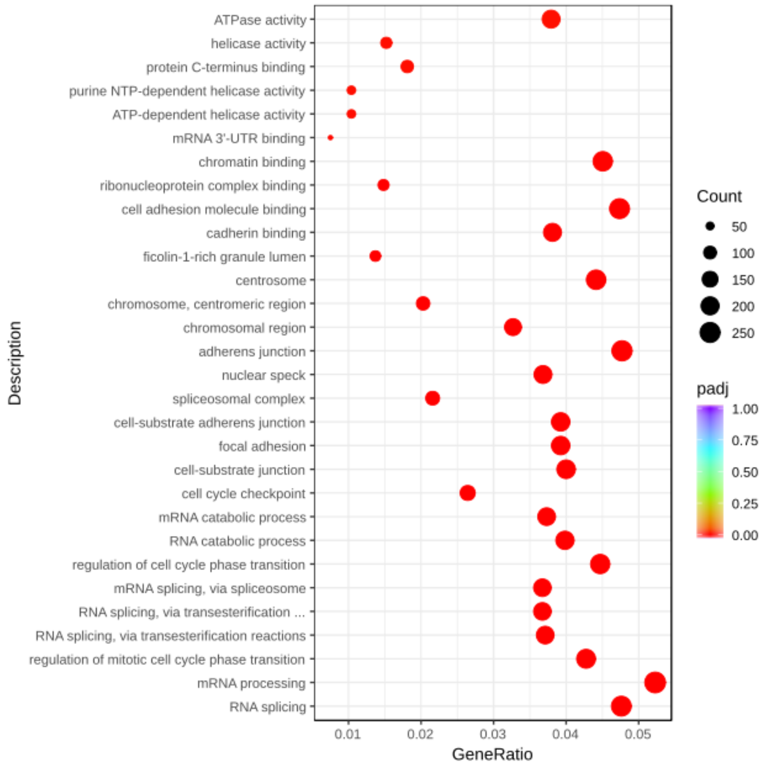

图3.15 GO富集分析散点图

图中横坐标为注释到GO Term上的差异基因数与差异基因总数的比值，纵坐标为GO Term

有向无环图(Directed Acyclic Graph，DAG)为差异基因GO富集分析结果的图形化展示方式。图中，分支代表包含关系，从上至下所定义的功能范围越来越小，选取每个差异比较组合的GO富集结果最显著性前5位的GO Term作为有向无环图的主节点，并通过包含关系，将相关联的GO Term一起展示，颜色的深浅代表富集程度。我们的项目中分别绘制生物过程、分子功能和细胞组分的DAG图。见结果文件：Enrichment/GO。

si_FGD3vssi_Control.GObp

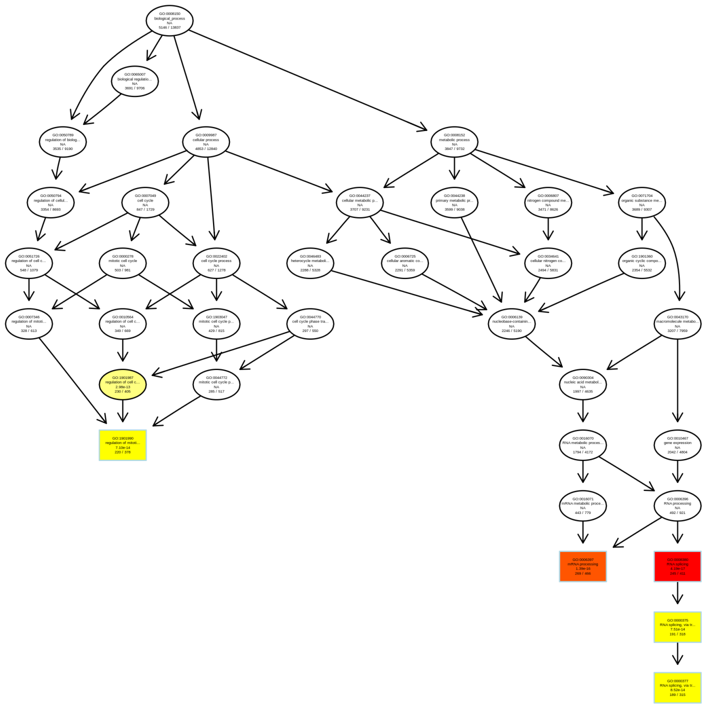

图3.16 GO富集分析DAG图

每个节点代表一个GO术语，方框代表的是富集程度为TOP5的GO，颜色的深浅代表富集程度，颜色越深就表示富集程度越高，每个节点上展示了该TERM的名称及富集分析的padj

3.5.2 KEGG通路富集分析

KEGG(Kyoto Encyclopedia of Genes and Genomes)是整合了基因组、化学和系统功能信息的综合性数据库。KEGG通路富集以padj小于0.05作为显著性富集的阈值，富集结果如下表所示，见结果文件：Enrichment/KEGG。

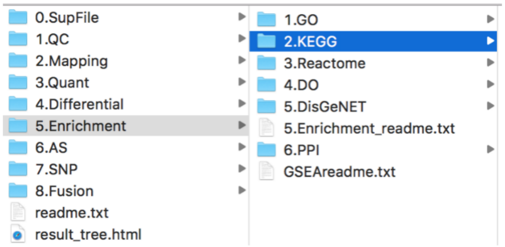

表3.10 KEGG富集分析部分结果展示

KEGGID	Description	GeneRatio	BgRatio	pvalue	padj
hsa04115	p53 signaling pathway	35/1462	72/5669	0.0000236	0.0072974
hsa04510	Focal adhesion	69/1462	178/5669	0.0000770	0.0118972
hsa05165	Human papillomavirus infection	104/1462	294/5669	0.0001167	0.0120163
hsa03040	Spliceosome	50/1462	123/5669	0.0001928	0.0148915
hsa05220	Chronic myeloid leukemia	33/1462	74/5669	0.0003235	0.0179939
hsa05110	Vibrio cholerae infection	23/1462	46/5669	0.0003494	0.0179939
hsa04142	Lysosome	46/1462	115/5669	0.0005268	0.0232529
hsa05222	Small cell lung cancer	36/1462	86/5669	0.0007750	0.0299333

- **KEGGID**：KEGG通路编号
- **Description**：KEGG通路编号对应的功能描述
- **GeneRatio**：注释到KEGG通路编号上的差异基因数与差异基因总数的比值
- **BgRatio**：注释到KEGG通路编号上的背景基因数与背景基因总数的比值
- **pvalue**：显著性检验p值
- **padj**：多重假设检验校正后的p值
- **geneID**：注释到KEGG通路编号上的差异基因ID
- **geneName**：注释到KEGG通路编号上的差异基因名称
- **keggID**：注释到KEGG通路编号上的差异基因keggID
- **Count**：注释到KEGG通路编号上的差异基因数
- **Up**: 与该Term相关的上调的差异基因个数
- **Up_Gene_id**: 与该Term相关的上调的差异基因ID
- **Down**: 与该Term相关的下调的差异基因个数
- **Down_Gene_id**: 与该Term相关的下调的差异基因ID

Novogene 诺禾致源 | 提供领先的基因科技解决方案 Providing leading genomic services & solutions 北京诺禾致源科技股份有限公司

从KEGG富集结果中，选取最显著的20个KEGG通路绘制柱状图进行展示，若不足20个，则绘制所有通路，如下图所示。图中横坐标为KEGG通路，纵坐标为通路富集的显著性水平，数值越高越显著，见结果文件：Enrichment/KEGG。

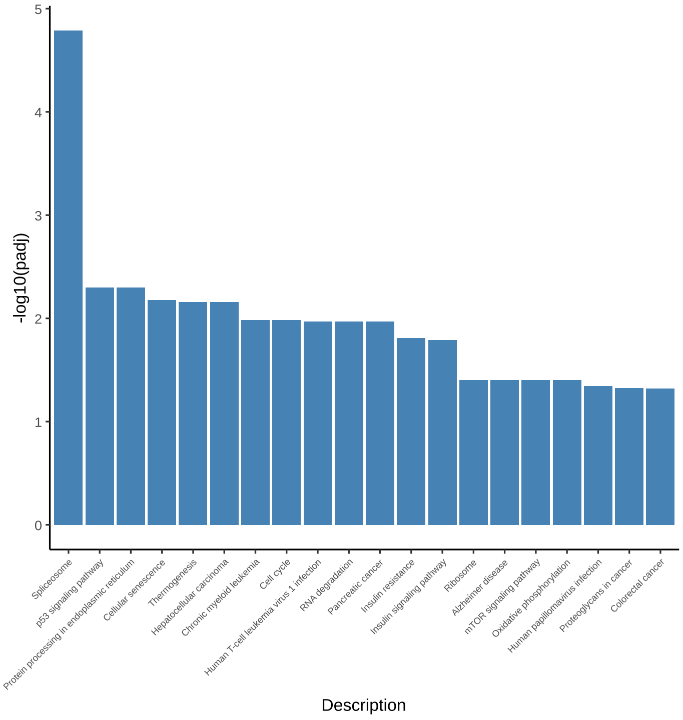

图3.17 KEGG富集分析柱状图

图中横坐标为KEGG通路，纵坐标为通路富集的显著性水平

从KEGG富集结果中，选取最显著的20个KEGG通路绘制散点图进行展示，若不足20个，则绘制所有通路，如下图所示。图中横坐标为注释到KEGG通路上的差异基因数与差异基因总数的比值，纵坐标为KEGG通路，点的大小代表注释到KEGG通路上的基因数，颜色从红到紫代表富集的显著性大小，见结果文件：Enrichment/KEGG。

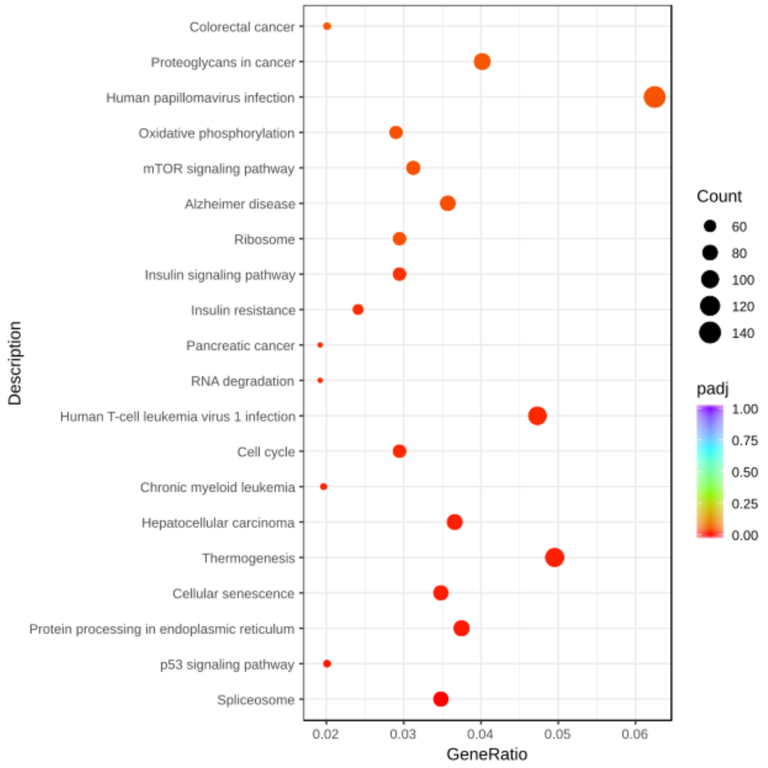

图3.18 KEGG富集散点图

图中横坐标为注释到KEGG通路上的差异基因数与差异基因总数的比值，纵坐标为KEGG通路

html文件可通过点击链接，交互查看显著富集的KEGG通路图。其中，包含上调基因的KEGG节点标红色，包含下调基因的KEGG节点标绿色，包含上下调的标黄色。鼠标悬停于标记的KEGG节点，弹出差异基因细节框，标色同上，括号中数字为log2(Foldchange)。以上步骤可脱机实现，如连接互联网，点击各个节点，可以连接到KEGG官方数据库中各个KEGG的具体信息页，见结果文件：Enrichment/KEGG。

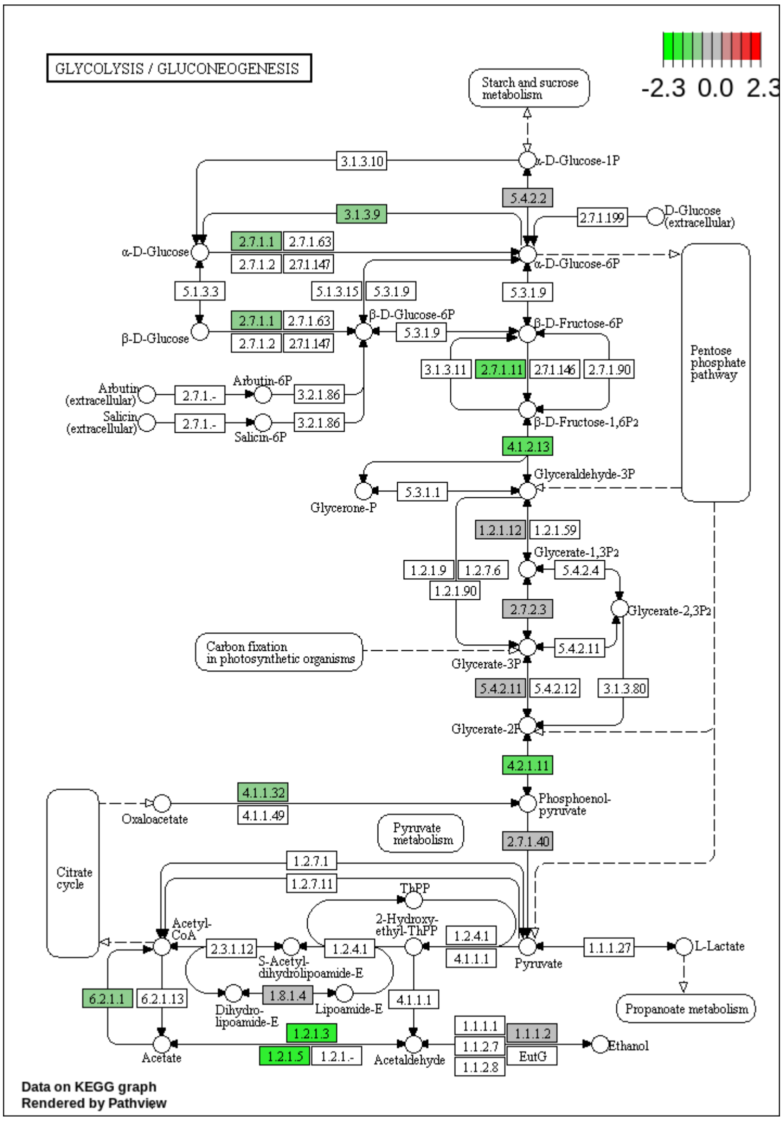

Novogene 诺禾致源 | 提供领先的基因科技解决方案 Providing leading genomic services & solutions   北京诺禾致源科技股份有限公司

3.5.3 Reactome功能富集分析

Reactome数据库汇集了人类等模式物种各项反应及生物学通路。Reactome通路富集以padj小于0.05作为显著性富集的阈值，富集结果如下表所示，见结果文件：Enrichment/Reactome。

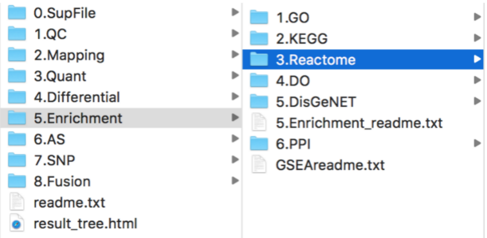

表3.11 Reactome富集分析部分结果展示

ReactomeID	Description	GeneRatio	BgRatio	pvalue	padj
R-HSA-72172	mRNA Splicing	83/2168	187/8559	0.00e+00	0.0000065
R-HSA-72163	mRNA Splicing - Major Pathway	80/2168	179/8559	0.00e+00	0.0000065
R-HSA-72203	Processing of Capped Intron-Containing Pre-mRNA	94/2168	236/8559	5.00e-07	0.0002075
R-HSA-114608	Platelet degranulation	51/2168	110/8559	1.30e-06	0.0003951
R-HSA-6798695	Neutrophil degranulation	143/2168	401/8559	1.60e-06	0.0004052
R-HSA-76005	Response to elevated platelet cytosolic Ca2+	51/2168	115/8559	6.30e-06	0.0013180
R-HSA-3700989	Transcriptional Regulation by TP53	125/2168	357/8559	2.03e-05	0.0036150
R-HSA-71387	Metabolism of carbohydrates	89/2168	244/8559	5.96e-05	0.0093024

- **ReactomeID**：Reactome通路编号
- **Description**：Reactome通路编号对应的功能描述
- **GeneRatio**：注释到Reactome通路编号上的差异基因数与差异基因总数的比值
- **BgRatio**：注释到Reactome通路编号上的背景基因数与背景基因总数的比值
- **pvalue**：显著性检验p值
- **padj**：多重假设检验校正后的p值
- **geneID**：注释到Reactome通路编号上的差异基因ID
- **geneName**：注释到Reactome通路编号上的差异基因名称
- **keggID**：注释到Reactome通路编号上的差异基因keggID
- **Count**：注释到Reactome通路编号上的差异基因数
- **Up**: 与该Term相关的上调的差异基因个数
- **Up_Gene_id**: 与该Term相关的上调的差异基因ID
- **Down**: 与该Term相关的下调的差异基因个数
- **Down_Gene_id**: 与该Term相关的下调的差异基因ID

Novogene 诺禾致源 | 提供领先的基因科技解决方案 Providing leading genomic services & solutions 北京诺禾致源科技股份有限公司

从Reactome富集分析结果中，选取最显著的20个Reactome通路绘制柱状图进行展示，若不足20个，则绘制所有通路，如下图所示。图中横坐标为Reactome通路，纵坐标为通路富集的显著性水平，数值越高越显著。见结果文件：Enrichment/Reactome

bsselect(state_plots,type="img",height='40%',width='60%',live_search=TRUE,show_tick=TRUE,style='btn-info')

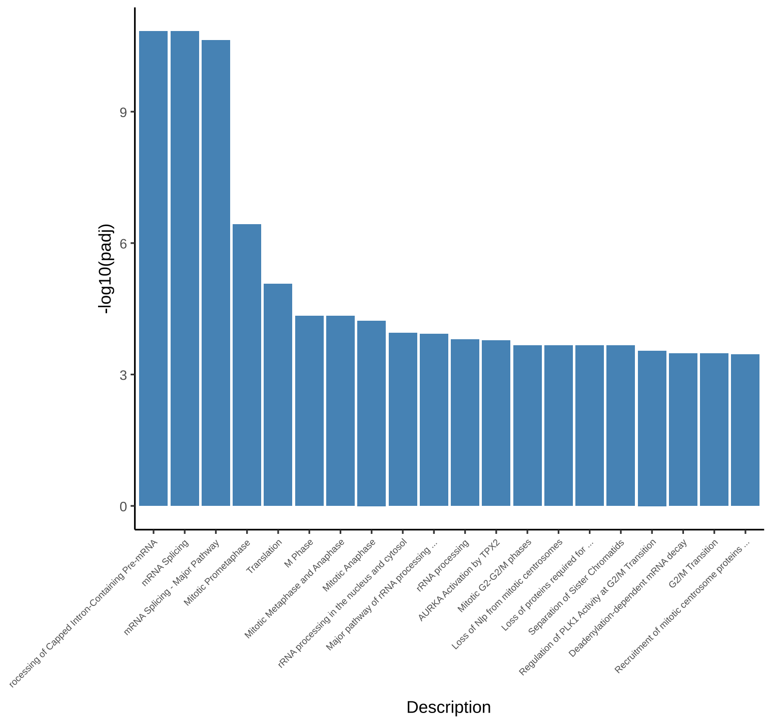

图3.19 Reactome富集分析柱状图

图中横坐标为Reactome通路，纵坐标为通路富集的显著性水平

从Reactome富集分析结果中，选取最显著的20个Reactome通路绘制散点图进行展示，若不足20个，则绘制所有通路，如下图所示。图中横坐标为注释到Reactome通路上的差异基因数与差异基因总数的比值，纵坐标为Reactome通路，点的大小代表注释到Reactome通路上的基因数，颜色从红到紫代表富集的显著性大小。见结果文件：Enrichment/Reactome

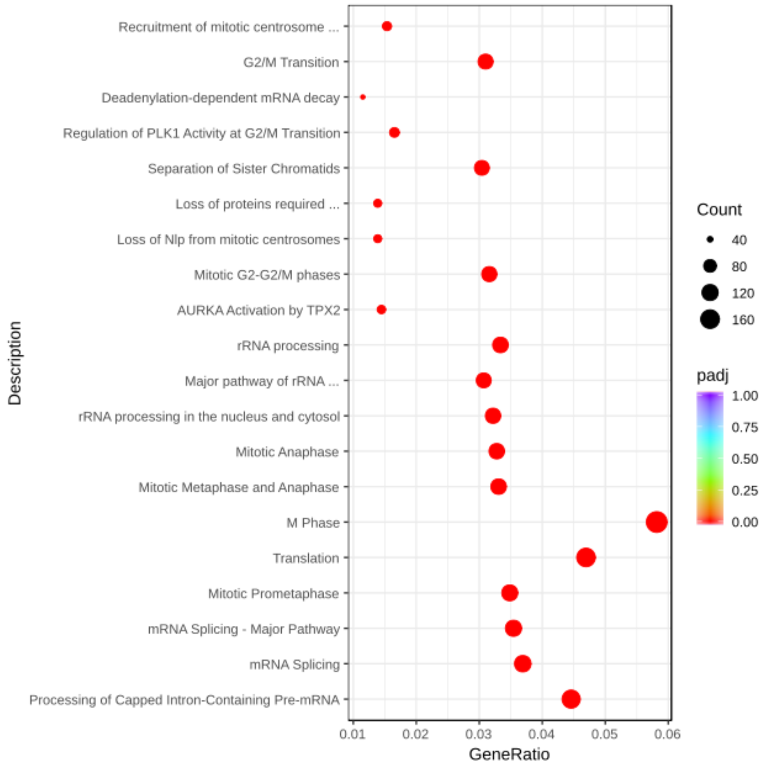

图3.20 Reactome富集分析散点图

图中横坐标为注释到Reactome通路上的差异基因数与差异基因总数的比值，纵坐标为Reactome通路，见结果文件：Enrichment/Reactome

3.5.4 DO功能富集分析

DO(Disease Ontology)是描述人类基因功能与疾病相关的数据库。DO富集以padj小于0.05作为显著性富集的阈值，富集结果下表如所示。见结果文件：Enrichment/DO

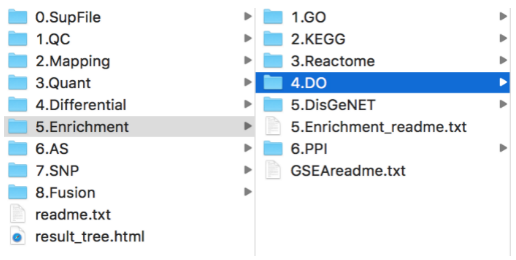

表3.12 DO富集分析部分结果展示

DOID	Description	GeneRatio	BgRatio	pvalue	padj
DOID:1612	breast cancer	159/1614	450/6195	0.0000038	0.0013638
DOID:5093	thoracic cancer	159/1614	450/6195	0.0000038	0.0013638
DOID:3459	breast carcinoma	123/1614	334/6195	0.0000054	0.0013638
DOID:3347	osteosarcoma	75/1614	194/6195	0.0000625	0.0119039
DOID:184	bone cancer	81/1614	216/6195	0.0001102	0.0167880
DOID:3905	lung carcinoma	143/1614	431/6195	0.0003844	0.0488194
DOID:201	connective tissue cancer	111/1614	325/6195	0.0005337	0.0580936
DOID:2355	anemia	68/1614	185/6195	0.0007338	0.0608994

- **DOID**：DO编号
- **Description**：DO编号对应的功能描述
- **GeneRatio**：注释到DO编号上的差异基因数与差异基因总数的比值
- **BgRatio**：注释到DO编号上的背景基因数与背景基因总数的比值
- **pvalue**：显著性检验p值

- **padj**：多重假设检验校正后的p值
- geneID**：注释到DO编号上的差异基因ID
- **geneName**：注释到DO编号上的差异基因名称
- **keggID**：注释到DO编号上的差异基因keggID
- **Count**：注释到DO编号上的差异基因数
- **Up**: 与该Term相关的上调的差异基因个数
- **Up_Gene_id**: 与该Term相关的上调的差异基因ID
- **Down**: 与该Term相关的下调的差异基因个数
- **Down_Gene_id**: 与该Term相关的下调的差异基因ID

从DO富集分析结果中，选取最显著的20个Term绘制柱状图进行展示，若不足20个，则绘制所有Term，如下图所示。图中横坐标为DO Term，纵坐标为Term富集的显著性水平，数值越高越显著。见结果文件：Enrichment/DO

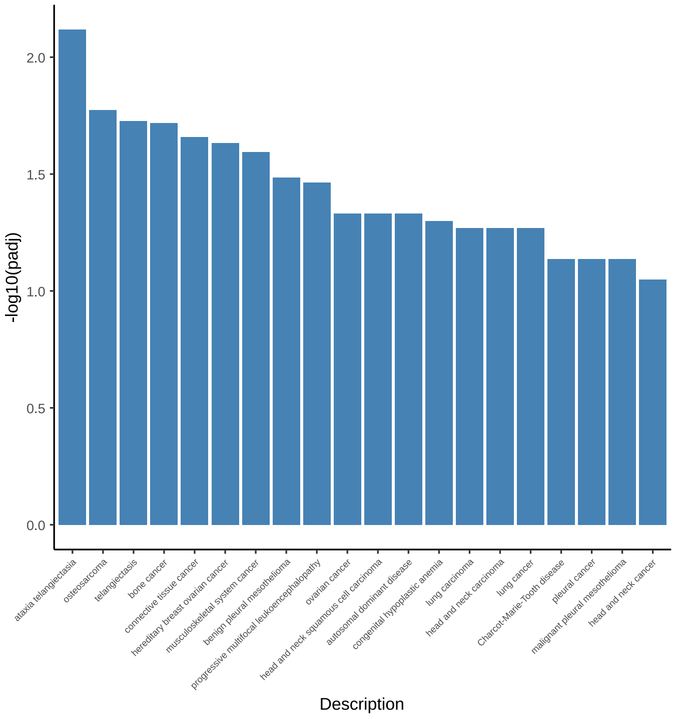

图3.21 DO富集分析柱状图

图中横坐标为DO Term，纵坐标为Term富集的显著性水平

从DO富集分析结果中，选取最显著的20个Term绘制散点图进行展示，若不足20个，则绘制所有Term，如下图所示。图中横坐标为注释到DO Term上的差异基因数与差异基因总数的比值，纵坐标为DO Term，点的大小代表注释到DO Term上的基因数，颜色从红到紫代表富集的显著性大小。见结果文件：Enrichment/DO

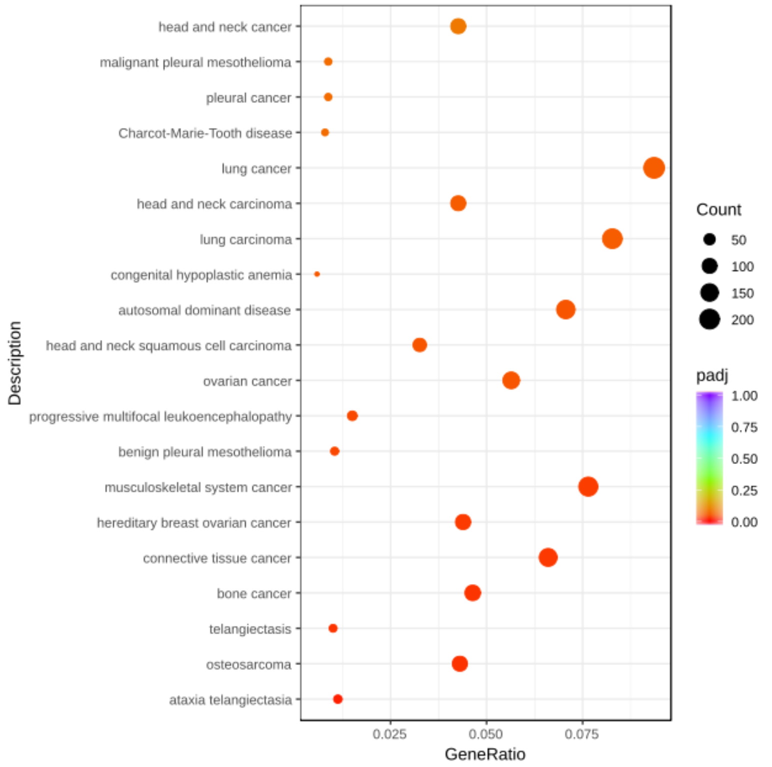

图3.22 DO富集分析散点图

图中横坐标为注释到DO Term上的差异基因数与差异基因总数的比值，纵坐标为DO Term

3.5.5 DisGeNET富集分析

DisGeNET数据库整合了人类疾病相关基因。DisGeNET富集以padj小于0.05作为显著性富集的阈值，富集结果如下表所示。见结果文件：Enrichment/DisGeNET

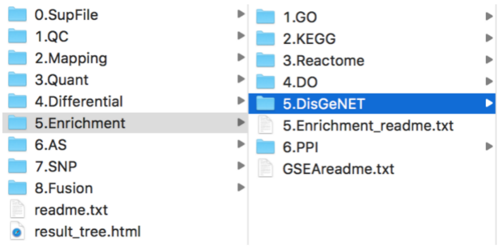

表3.13 DisGeNET富集分析部分结果展示

DisGeNETID	Description	GeneRatio	BgRatio	pvalue	padj
C0279000	Liver and Intrahepatic Biliary Tract Carcinoma	160/2759	459/11754	0.0e+00	0.0000585
C0025286	Meningioma	127/2759	357/11754	1.0e-07	0.0002125
C0023487	Acute Promyelocytic Leukemia	132/2759	377/11754	2.0e-07	0.0002125
C0040136	Thyroid Neoplasm	134/2759	384/11754	2.0e-07	0.0002125
C0016057	Fibrosarcoma	94/2759	249/11754	2.0e-07	0.0002267
C0266470	Cerebellar Hypoplasia	34/2759	65/11754	4.0e-07	0.0002884
C0006625	Cachexia	52/2759	117/11754	4.0e-07	0.0002884
C1512409	Hepatocarcinogenesis	147/2759	442/11754	1.2e-06	0.0007161

- **DisGeNETID**：DisGeNET数据库疾病编号
- **Description**：疾病编号对应的功能描述
- **GeneRatio**：注释到该疾病编号上的差异基因数与差异基因总数的比值
- **BgRatio**：注释到该疾病编号上的背景基因数与背景基因总数的比值
- **pvalue**：显著性检验p值

- **padj**：多重假设检验校正后的p值
- **geneID**：注释到疾病编号上的差异基因ID
- **geneName**：注释到疾病编号上的差异基因名称
- **Count**：注释到疾病编号上的差异基因数
- **Up**: 与该Term相关的上调的差异基因个数
- **Up_Gene_id**: 与该Term相关的上调的差异基因ID
- **Down**: 与该Term相关的下调的差异基因个数
- **Down_Gene_id**: 与该Term相关的下调的差异基因ID

从DisGeNET富集分析结果中，选取最显著的20个Term绘制柱状图进行展示，若不足20个，则绘制所有Term，如下图所示。图中横坐标为DisGeNET Term，纵坐标为Term富集的显著性水平，数值越高越显著。见结果文件：Enrichment/DisGeNET

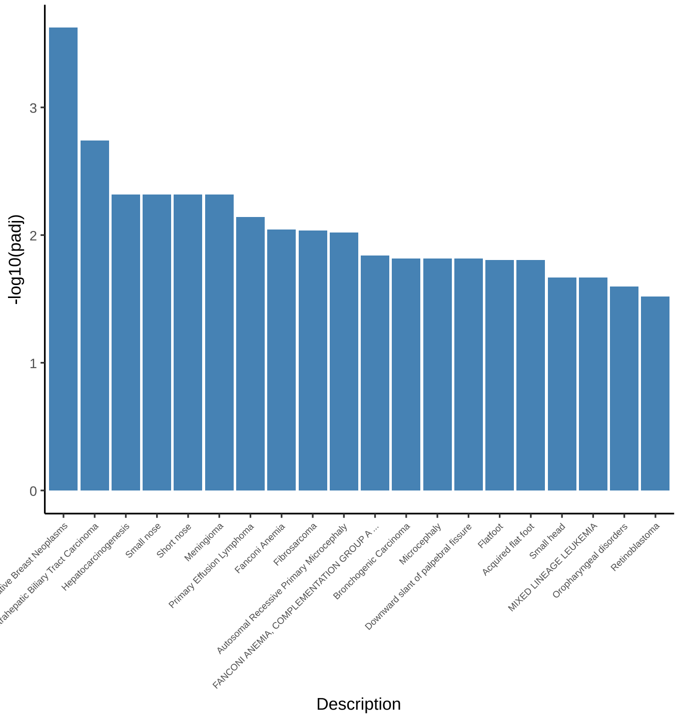

图3.23 DisGeNET富集分析柱状图

图中横坐标为DisGeNET Term，纵坐标为Term富集的显著性水平

从DisGeNET富集分析结果中，选取最显著的20个Term绘制散点图进行展示，若不足20个，则绘制所有Term，如下图所示。图中横坐标为注释到DisGeNET Term上的差异基因数与差异基因总数的比值，纵坐标为DisGeNET Term，点的大小代表注释到DisGeNET Term上的基因数，颜色从红到紫代表富集的显著性大小。见结果文件：Enrichment/DisGeNET

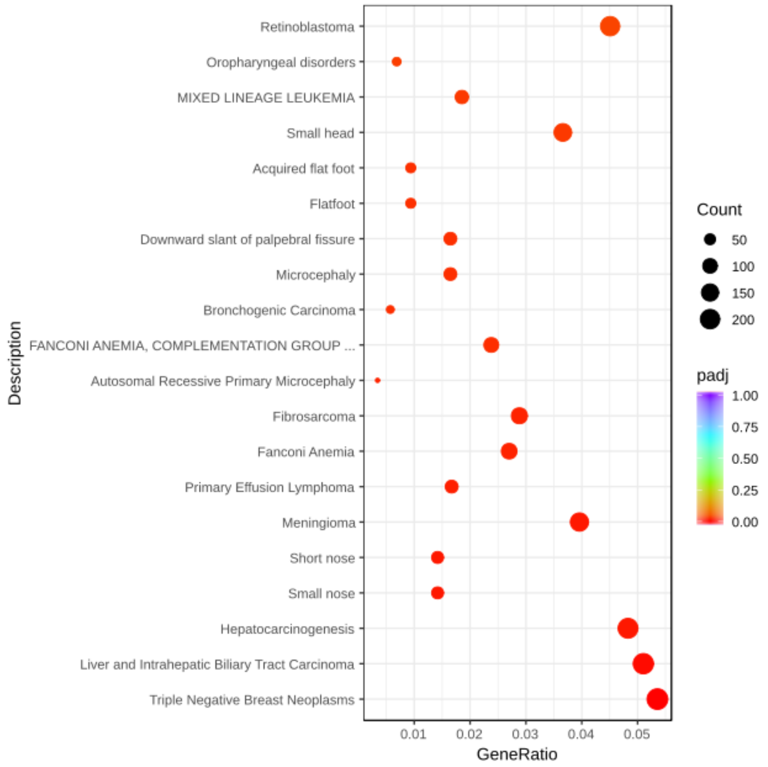

图3.24 DisGeNET富集分析散点图

图中横坐标为注释到DisGeNET Term上的差异基因数与差异基因总数的比值，纵坐标为DisGeNET Term

提供领先的基因科技解决方案
Providing leading genomic services & solutions
北京诺禾致源科技股份有限公司

3.5.6 GSEA富集分析

常规的基于超几何分布的富集分析依赖于显著上调或下调的基因，容易遗漏部分差异表达不显著但有重要生物学意义的基因。基因富集分析（GSEA）不需要指定明确的差异基因阈值，把基因按照在两组样本中的差异表达程度进行排序，然后采用统计学方法检验预先设定的基因集合是否在排序表的顶端或低段富集。GSEA主要包括三个步骤：计算富集得分（Enrichment Score）；估计富集得分的显著性水平；多重假设检验。见结果文件：Enrichment/{比较组合}/GSEA

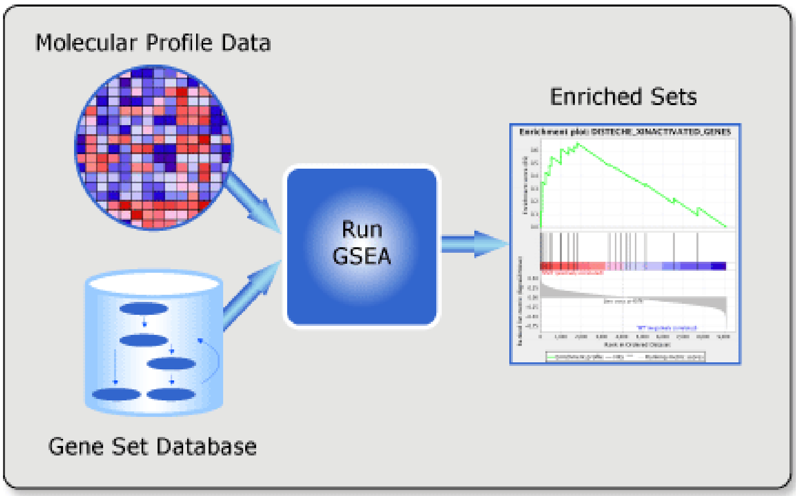

我们分别对该物种的GO、KEGG、Reactome、DO、DisGeNET等数据集进行GSEA分析，结果如下所示。

GSEA详细的富集原理和结果文件请点击GSEA (src/GSEA_ref_readme.pdf)

si_FGD3vssi_Control_DisGeN

GSEA Report for Dataset fpkm

Enrichment in phenotype: si_FGD3 (3 samples)

- 786 / 4286 gene sets are upregulated in phenotype **si_FGD3**
- 0 gene sets are significant at FDR < 25%
- 47 gene sets are significantly enriched at nominal pvalue < 1%
- 47 gene sets are significantly enriched at nominal pvalue < 5%
- Snapshot of enrichment results
- Detailed enrichment results in html format
- Detailed enrichment results in excel format (tab delimited text)
- Guide to interpret results

Enrichment in phenotype: si_Control (3 samples)

- 3500 / 4286 gene sets are upregulated in phenotype **si_Control**
- 0 gene sets are significantly enriched at FDR < 25%
- 1188 gene sets are significantly enriched at nominal pvalue < 1%
- 1188 gene sets are significantly enriched at nominal pvalue < 5%
- Snapshot of enrichment results
- Detailed enrichment results in html format
- Detailed enrichment results in excel format (tab delimited text)
- Guide to interpret results

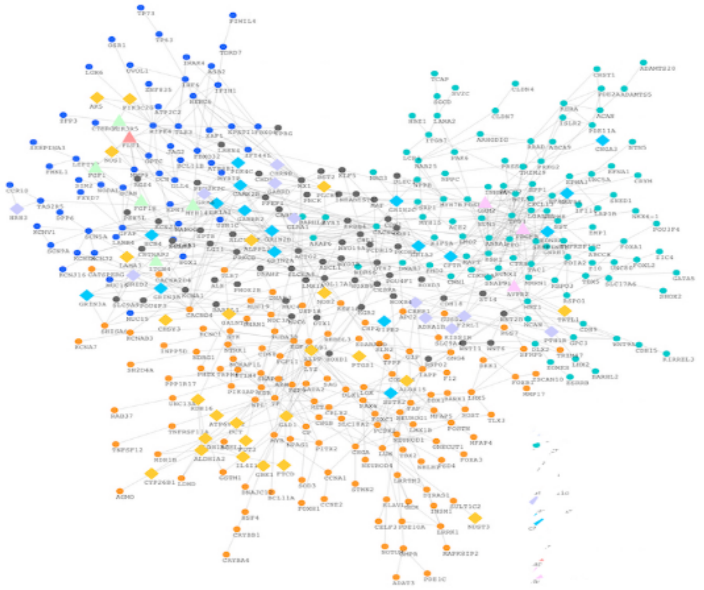

图3.25 蛋白互作网络图

注：图中每个节点表示一个蛋白，每一条连线表示相连蛋白间的相互作用

我们结果文件中分析的结果是利用R包中的STRINGdb进行PPI的互作网络图的绘制。由于STRINGdb包只能最多绘制400个蛋白的互作网络图，如果蛋白的个数大于400的话，我们选取蛋白对应的差异基因最显著的前400个基因进行互作网络图的绘制。

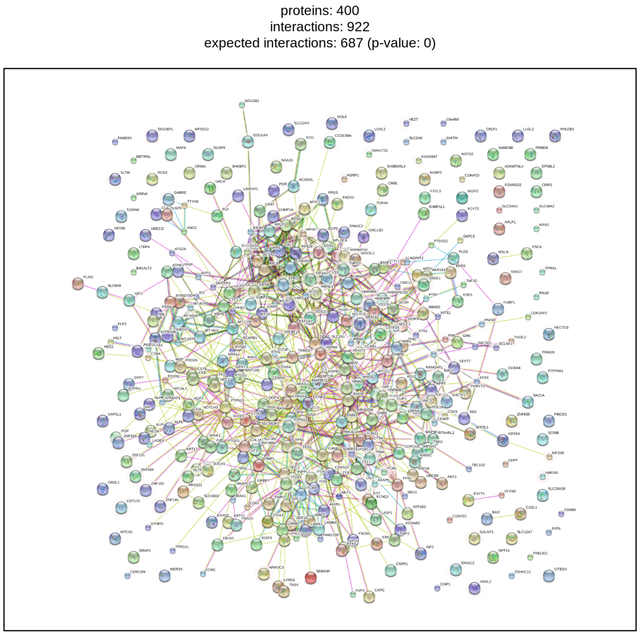

http://version10.string-db.org/10/p/9785577147

图3.26 蛋白互作网络图

图中的protiens表示能够注释到string数据库中的蛋白的个数，interactions表示的是两个蛋白之间相互作用的个数。我们插入一个pValue，它表示您可以通过随机的方式期望这样一个相等或更大数量的交互的可能性。此外，在底部，有一个简短的URL指向我们的Web界面上的相关页面

3.5.8 富集分析常见问题及解答

富集分析原理图中n，N，i，M的含义?

富集分析结果中，GeneRatio的这一列表示的是差异基因集信息。其中斜杠左侧的数字表示注释到某个通路的差异基因数，该值对应于富集原理图的i(某通路差异基因集)，斜杠右侧的数字表示注释到所有通路的差异基因数，对应于富集分析原理图的n(差异基因集)。BgRatio的这一列表示的是背景基因集信息，其中斜杠左侧的数字是注释到某个通路的背景基因数，对应于富集分析原理图的M(某通路基背景基因)，斜杠

右侧的注释到所有通路的背景基因数，对应于富集分析原理图的N(所以通路背景基因集)。

富集分析结果只看显著性富集的通路吗?

对富集分析结果的解读，应该从生物学意义的角度来看，pvalue和padj只是提供一个参考，只要结果可以解释，有生物学意义，那些不显著富集的通路也值得深入研究的，不用执着于pvalue或padj。

Novogene 诺禾致源 | 提供领先的基因科技解决方案 Providing leading genomic services & solutions　　北京诺禾致源科技股份有限公司

3.6 可变剪接分析

除了表达分析，根据reads与参考基因组比对情况，我们还可以利用RNA-seq数据进行可变剪接、SNP/indel等变异位点分析。

可变剪接(Alternative Splicing, AS)，是大多数真核生物细胞中普遍的一种基因表达方式。真核细胞的基因序列包含内含子(intron)与外显子(exon)，在基因转录成mRNA前体后内含子会被RNA剪切体移除，而外显子则保留于成熟mRNA中。一条未经剪切的RNA，可以具有多种外显子剪切形式，因此使得一个基因在不同时间、不同环境中可以翻译出不同的蛋白质，进而增加其生理状况下系统的复杂性或适应性。

rMATS (http://rnaseq-mats.sourceforge.net/index.html)是一款适用于RNA-Seq数据的可变剪接分析软件[16]，它不仅可以对变剪接事件进行分类，还可以进行不同样本间可变剪接事件的差异分析。rMATS软件对可变剪接事件分类如下图所示：

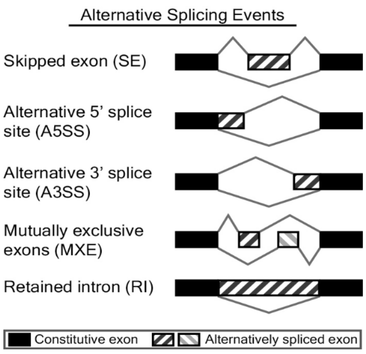

图3.27 可变剪接事件分类

- **Skipped exon**：外显子跳跃
- **MXE: Mutually exclusive exon**： 外显子互斥
- **A5SS: Alternative 5' splice site**：5'端外显子发生可变剪接
- **A3SS: Alternative 3' splice site**：3'端外显子发生可变剪接
- **RI: Retained intron**：内含子滞留

Novogene 诺禾致源 | 提供领先的基因科技解决方案 Providing leading genomic services & solutions　　北京诺禾致源科技股份有限公司

3.6.1 差异可变剪接事件

我们使用rMATS软件进行AS事件的定量及差异分析。rMATS软件可以将AS事件进行5种分类，并且可以对有生物学重复的样品进行差异AS分析。每个可变剪接事件对应两个Isoform，分别为Exon Inclusion Isoform和Exon Skipping Isoform，分别对两个Isoform进行表达量统计，并除以其有效长度，得到校正后的表达量，然后计算Exon Inclusion Isoform在两个Isoform总表达量的比值，比值即为结果文件中的IncLevel1（处理组）和IncLevel2（对照组），如下图所示。最后进行差异显著性分析。我们筛选差异显著性可变剪接事件的阈值是FDR小于0.05，SE类型可变剪接事件的差异分析结果如下表所示，见结果文件：AS/1.daslist。

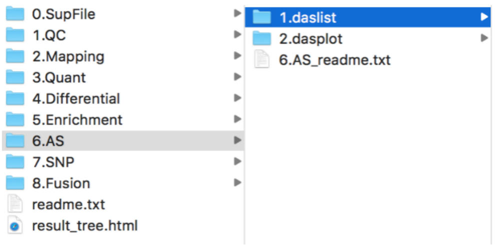

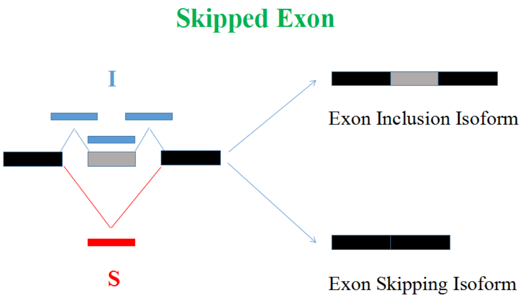

图3.28 SE类型可变剪接类型

注：图中灰色方框表示发生可变剪切的外显子，黑色为组成型外显子

表3.14 SE类型差异可变剪接分析部分结果展示

GeneID	geneSymbol	chr	strand	exonStart_0base	exonEnd	upstreamES	upstreamEE
ENSG00000130600	H19	11	•	1995875	1995941	1995175	1995794
ENSG00000122566	HNRNPA2B1	7	•	26190992	26191128	26190124	26190460
ENSG00000204120	GIGYF2	2	•	232700584	232700654	232697369	232697392
ENSG00000034677	RNF19A	8	•	100300527	100300651	100287500	100288267
ENSG00000256500	AL139300.1	14	•	103571622	103571820	103562961	103563124
ENSG00000015133	CCDC88C	14	•	91285394	91285836	91283328	91283517
ENSG00000167670	CHAF1A	19	•	4405911	4405962	4402661	4402814
ENSG00000129744	ART1	11	•	3655503	3655655	3645127	3645179

- **GeneID**：可变剪接事件所在基因编号
- **geneSymbol**：可变剪接事件所在基因名称
- **chr**：可变剪接事件所在染色体
- **strand**：可变剪接事件所在染色体链的方向
- **exonStart_0base**：可变剪接事件跳跃外显子起始位置，以0开始计数
- **exonEnd**：可变剪接事件跳跃外显子终止位置
- **upstreamES**：可变剪接事件跳跃外显子的上游exon起始位置
- **upstreamEE**：可变剪接事件跳跃外显子的上游exon终止位置
- **downstreamES**：可变剪接事件跳跃外显子的下游exon起始位置
- **downstreamEE**：可变剪接事件跳跃外显子的下游exon终止位置
- **IC_SAMPLE_1**：可变剪接事件Exon Inclusion Isoform在差异比较组合中处理组的表达量
- **SC_SAMPLE_1**：可变剪接事件Exon Skipping Isoform在差异比较组合中处理组的表达量
- **IC_SAMPLE_2**：可变剪接事件Exon Inclusion Isoform在差异比较组合中对照组的表达量
- **SC_SAMPLE_2**：可变剪接事件Exon Skipping Isoform在差异比较组合中对照组的表达量
- **IncFormLen**：可变剪接事件Exon Inclusion Isoform的有效长度
- **SkipFormLen**：可变剪接事件Exon Skipping Isoform的有效长度
- **PValue**：可变剪接事件表达差异显著性p值
- **FDR**：可变剪接事件表达差异显著性FDR值
- **IncLevel1**：处理组可变剪接事件Exon Inclusion Isoform在两个Isoform总表达量的比值
- **IncLevel2**：对照组可变剪接事件Exon Inclusion Isoform在两个Isoform总表达量的比值
- **IncLevelDifference**：IncLevel1与IncLevel2的差值

3.6.2 差异剪接可视化

对于表达差异显著性的可变剪接事件，进行可视化展示，SE类型可变剪接事件的可视化展示如下图所示：图中标题为可变剪接事件三个外显子所在的染色体坐标及正负链信息，跨外显子比对的reads使用连接外显子junction边界的弧线表示。弧线的粗细和比对到junction上的reads数成正比，同时弧线上的数字指出了junction reads的数目，右上方标注了各个样本可变剪接事件所在的基因及IncLevel值。见结果文件：AS/2.dasplot。

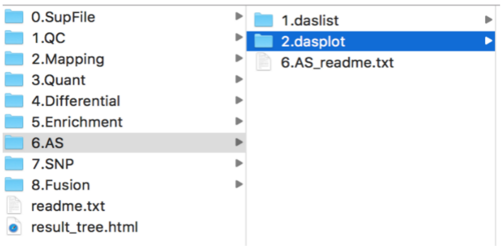

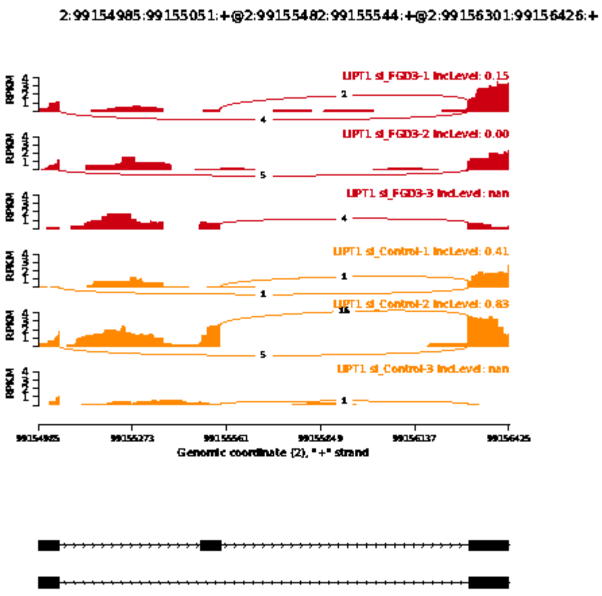

图3.29 SE类型可变剪接可视化展示

注：图中红色和橘黄色分别表示不同的样本，inclusion level值代表exon Inclusion Isoform在两个Isoform总表达量的比值，可以直接看出不同样本中可变剪接事件的表达差异

3.7 变异位点分析

SNP全称Single Nucleotide Polymorphisms，是指在基因组上由单个核苷酸变异形成的遗传标记，其数量很多，多态性丰富。一般而言，SNP是指变异频率大于1%的单核苷酸变异。InDel(insertion-deletion)是指相对于参考基因组，样本中发生的小片段的插入缺失，该插入缺失可能含一个或多个碱基。

变异位点分析是RNA-seq结构分析的重要内容，主要包括先天变异位点和后天体细胞突变位点的检测，对肿瘤等研究具有重要意义。我们使用GATK软件对样本数据进行变异位点分析，并用SnpEff软件对变异位点进行注释[18]。

3.7.1 变异位点检测

变异位点主要分为SNP与INDEL，每个位点的基因型及注释如下表所示，见结果文件：SNP/2.snpeff。

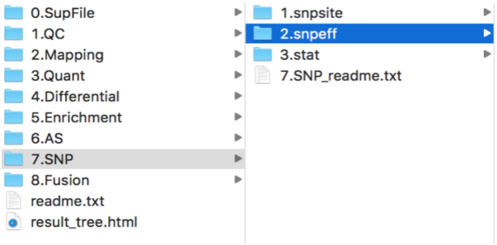

表3.15 变异位点类型及其注释

CHROM	POS	ID	REF	ALT	QUAL	DP	AD
1	14653	.	C	T	700.77	100	53,29
1	14653	.	C	T	700.77	100	53,29
1	14653	.	C	T	700.77	100	53,29
1	14653	.	C	T	700.77	100	53,29
1	15820	.	G	T	153.73	21	13,8
1	15820	.	G	T	153.73	21	13,8
1	15820	.	G	T	153.73	21	13,8
1	15820	.	G	T	153.73	21	13,8

- **CHROM**：变异位点所在的染色体编号
- **POS**：变异位点所在的染色体坐标
- **ID**：变异位点编号，同时对应着dbSNP数据库中的ID，若没有，则默认使用‘.’
- **REF**：变异位点在参考基因组中的碱基
- **ALT**：变异位点在样本中的实际碱基
- **QUAL**：变异位点质量，Phred格式的数值，数值越高，变异位点基因型的可信度越高
- **DP**：变异位点在样本中的测序深度
- **AD**：分别支持REF与ALT的reads数，中间以逗号相隔
- **GT**：变异位点的基因型
- **GeneID**：变异位点所在的基因编号
- **GeneName**：变异位点所在的基因名称
- **FeatureID**：变异位点所在的转录本编号
- **Biotype**：变异位点所在的转录本类型
- **HGVS_C**：变异位点在DNA水平的HGVS注释
- **HGVS_P**：变异位点在蛋白水平的HGVS注释，仅针对编码蛋白基因
- **EFFECT**：变异位点所造成的影响
- **IMPACT**：变异位点所造成的影响程度

3.7.2 变异位点统计

使用GATK进行变异位点的检测后，我们会根据SnpEff注释信息对每个变异位点进行统计，主要为变异位点功能统计、变异位点区域统计以及变异位点影响统计。变异位点功能（snp_function）从同义突变、错义突变、无义突变三个方面进行统计和绘图。变异位点区域（snp_region）从EXON、INTRON、INTERGENIC等基因结构区域进行统计和绘图。变异位点影响（snp_impact）从HIGH(高)、MODERATE（中）、LOW（低）、MODIFIER（自身无表型效应，和别的突变位点同时存在才会产生影响）四个层次进行统计和绘图，见结果文件：SNP/3.stat。

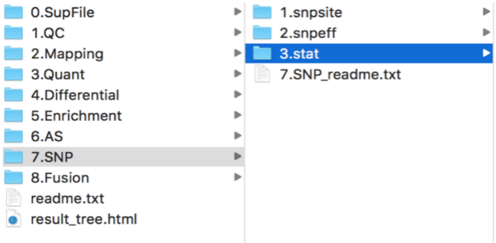

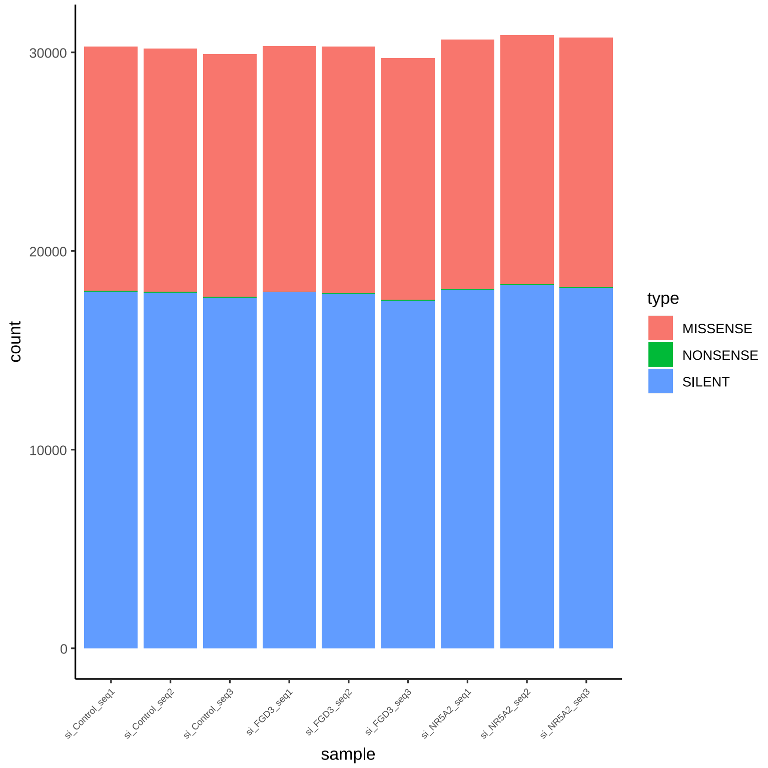

图3.30 变异位点区域统计

注：图中横坐标表示样本名称，纵坐标表示不同基因组区域（EXON、INTRON、INTERGENIC）变异的数量

3.7.3 变异位点分析常见问题及解答

SNP分析的参考序列是什么，即REF是指什么?

参考序列REF是选取的参考基因组序列，SNP是通过将reads比对到参考基因组上从而进行SNP calling。

SNP中一列中两个数字分别代表支持REF和ALT两种碱基的reads数目，为什么有些会是0呢?

以0,12为例，表示有0个reads支持REF的碱基，即没有支持该碱基的reads，12个reads支持ALT的碱基。

SNP具体指什么？其与InDel有何区别?

一般而言，SNP是指变异频率大于1%的单核苷酸变异。InDel(insertion-deletion)则是插入或者缺失，insert或者deletion。

GT列含义?

GT表示样本的基因型，假如一个二倍体生物样本，其基因组有两个拷贝，两个拷贝的同一位置是等位位点，GT值表示该样本在某位点携带的两个等位位点，主要用三个数值0，1，2来描述。0表示跟REF一致，1表示跟第一个ALT一致，2表示跟第二个ALT一致。0/0表示纯合，且该样本的两个拷贝的等位位点跟REF一致。0/1表示杂合，且该样本的一个拷贝跟REF一致，另一个拷贝跟ALT一致。0/2表示杂合，且该样本的一个拷贝跟REF一致，另一个拷贝跟第二个ALT一致。1/1表示纯合，且该样本的两个拷贝的等位位点跟ALT一致。1/2表示杂合，且该样本的一个拷贝跟第一个ALT一致，另一个拷贝跟第二个ALT一致。2/2表示纯合，且该样本的两个拷贝跟第二个ALT一致。根据遗传学理论，一个样本出现两个ALT的可能性是非常低的。

何为HGVS注释?

HGVS是Human Genome Variation Society的缩写，国际上一般以HGVS推荐的命令规则来对基因突变位点进行注释，具体规则和示例可以参考DNA (http://www.hgvs.org/mutnomen/examplesDNA.html)和protein (http://www.hgvs.org/mutnomen/examplesAA.html)

3.8 融合基因分析

融合基因是指两个基因的全部或部分序列融合而成的嵌合基因，一般由染色体易位、缺失等原因所致。融合基因首次发现于血液系统的恶性肿瘤中，其中以慢性粒细胞白血病中BCR-ABL的基因融合最为经典，治疗慢性粒细胞白血病的药物伊马替尼/格列卫，其作用靶点就是该融合基因。高通量RNA测序技术因其通量高、成本低、检测精度高检测范围广等优点大大加快了融合基因的研究，诺禾致源使用STAR-Fusion软件进行融合基因的检测，STAR-Fusion（链接）是利用STAR比对的融合输出结果来检测融合转录本的软件包，分为STAR比对，STAR-Fusion.predict，STAR-Fusion.filter，具体原理可参照附录：

3.8.1 融合基因列表

融合基因列表如下所示。其中JunctionReads和SpanningFrags是检测融合基因的主要指标；在双端测序中，可以把read1和read2看成是一个fragment片段（250bp-300bp）的两端，若read1或read2跨越融合事件的断点，这样的read称为JunctionRead。read1和read2中间的gap区域横跨融合事件的断点，这样的fragment片段称为SpanningFrag，见结果文件：Fusion/1.fusionlist。

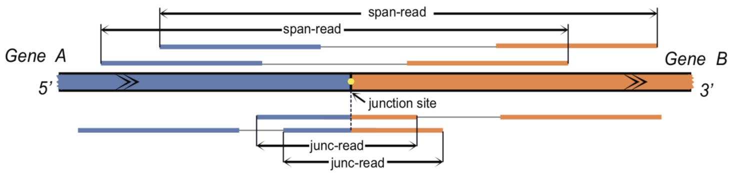

图3.23 JunctionReads和SpanningFrags示意图

见结果文件：Fusion/1.fusionlist。

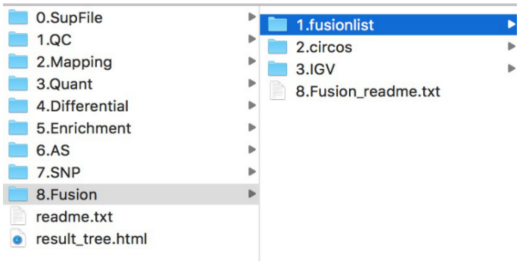

表3.16 融合基因列表

FusionName	JunctionReadCount	SpanningFragCount	LeftGene	LeftLocalBreakpoint	LeftBreakpoint
HNRNPUL2–C11orf49	45	1	HNRNPUL2^ENSG00000214753.2	1731	chr11:62726619:-
MFSD12–PIP5K1C	40	6	MFSD12^ENSG00000161091.12	13649	chr19:3542776:-
GLG1–FA2H	25	1	GLG1^ENSG00000090863.11	3741	chr16:74532121:-
MFSD12–PIP5K1C	25	1	MFSD12^ENSG00000161091.12	10485	chr19:3546074:-
B3GAT3–GANAB	17	1	B3GAT3^ENSG00000149541.9	2679	chr11:62620497:-
GNB1–NADK	16	5	GNB1^ENSG00000078369.17	1298	chr1:1890820:-
ASAP1–AC090987.1	12	0	ASAP1^ENSG00000153317.14	3356	chr8:130401885:-
MT-ND5–MT-ATP8	10	0	MT-ND5^ENSG00000198786.2	2730	chrM:14066:+

- **FusionName**：融合事件名称
- **JunctionReadCount**：支持融合事件发生的junction reads数目
- **SpanningFragCount**：支持融合事件发生的Spanning fragments数目
- **LeftGene**：融合事件的上游基因，包括基因ID和基因名称，其中基因ID为GENCODE数据库ID，去掉小数点和后面的数字即可与前面的一致。
- **LeftLocalBreakpoint**：上游基因在基因序列上的断点位置(断点位置是从每个基因序列的5'端开始)
- **LeftBreakpoint**：上游基因断点所在的基因组坐标和上游基因所在基因组的正负链信息
- **RightGene**：融合事件的下游基因
- **RightLocalBreakpoint**：下游基因在基因序列上的断点位置(断点位置是从每个基因序列的5'端开始)
- **RightBreakpoint**：下游基因断点所在的基因组坐标和下游基因所在基因组的正负链信息
- **SpliceType**：鉴定的融合断点是否位于已知基因结构的外显子剪接位点
- **annots**：融合事件上下游基因的功能注释信息
- **CDS_LEFT_ID**：上游基因具有CDS区域的转录本ID
- **CDS_LEFT_RANGE**：上游基因具有CDS区域的转录本的碱基范围(bp)
- **CDS_RIGHT_ID**：下游基因具有CDS区域的转录本的ID

- **CDS_RIGHT_RANGE**：下游基因具有CDS区域的转录本的碱基范围(bp)
- **PROT_FUSION_TYPE**：编码基因的融合类型，FRAMESHIFT(阅读框发生移码)，INFRAME(阅读框未发生移码)
- **FUSION_CDS**：融合基因的CDS序列，小写的碱基是上游融合基因的CDS序列，大写是碱基是下游融合基因的CDS序列
- **FUSION_TRANSL**：翻译得到的融合基因的氨基酸序列
- **PFAM_LEFT**：上游基因的PFAM注释，如DUF1515|572-611|0.073，其中DUF1515为PFAM预测的得到的蛋白结构域，572-611为预测的结构域的碱基范围，0.073是预测和DUF15的相似度
- **PFAM_RIGHT**：下游基因的PFAM注释

Novogene 诺禾致源 | 提供领先的基因科技解决方案 Providing leading genomic services & solutions 北京诺禾致源科技股份有限公司

3.8.2 融合基因染色体分布

融合基因事件在染色体上的分布用circos图进行展示，图中的环由多条染色体组成，环内每条线代表一个融合事件，线的两端代表融合事件的断点位置，红色表示融合基因的上下游基因来源于同一条染色体，蓝色表示融合基因的上下游基因来源于不同染色体，线的粗细代表融合基因的表达水平，见结果文件：Fusion/2.circos。

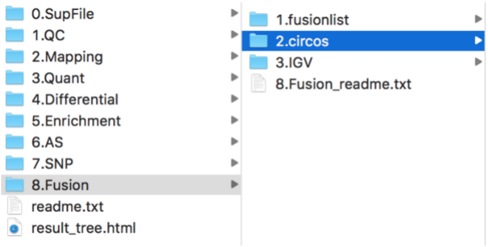

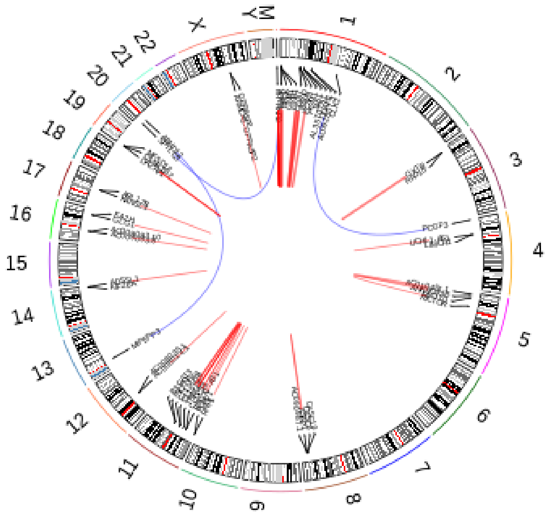

图3.31 融合基因染色体分布

Novogene 诺禾致源 | 提供领先的基因科技解决方案 Providing leading genomic services & solutions 北京诺禾致源科技股份有限公司

3.8.3 融合基因IGV可视化展示

融合基因事件可以通过IGV软件进行可视化，如下图所示。

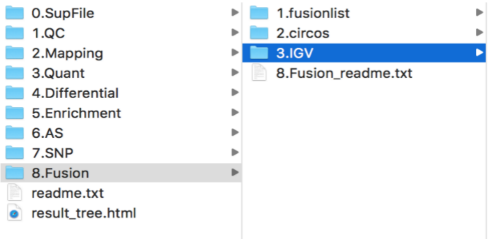

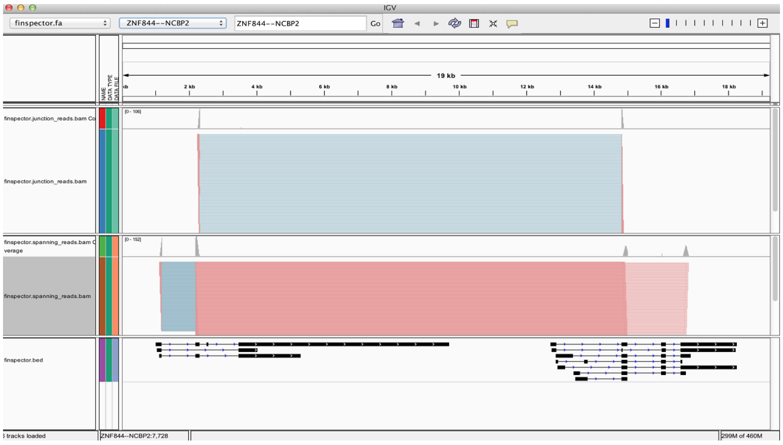

图3.32 融合基因染色体分布

将结果文件中融合基因文件夹里每个样本的融合基因序列文件，融合基因结构注释文件（提供bed和gtf两种格式的注释文件，使用其中一个即可），比对的bam文件分别导入到IGV软件，具体文件说明，如下所示，见结果文件：Fusion/3.IGV。

- **sample.fa**：融合基因序列文件，fasta格式
- **sample.bed**：融合基因结构注释文件，bed格式
- **sample.gtf**：融合基因结构注释文件，gtf格式
- **sample.junction_reads.bam**：支持融合事件的JunctionReads的bam文件
- **sample.spanning_reads.bam**：支持融合事件的SpanningFrags的bam文件

Novogene 诺禾致源 | 提供领先的基因科技解决方案 Providing leading genomic services & solutions 北京诺禾致源科技股份有限公司

3.8.4 融合基因分析常见问题及解答

融合基因验证该挑选哪些融合基因?

优先挑选高表达的融合基因进行验证，高表达的融合基因事件假阳性更低，也更容易实验验证，不过低表达的融合基因事件同样具有研究价值，比如很多抑癌基因都是低表达的，这些基因发生融合，可能导致重要功能丢失。

4 附录

4.1 结果文件解压方法

压缩格式	用户类型	方法
**file.tar.gz形式的压缩文件**	Unix/Linux/Mac用户	使用tar -zxvf file.tar.gz命令
	Windows用户	使用解压缩软件如WinRAR、7-Zip等
**file.gz形式的压缩文件**	Unix/Linux/Mac用户	使用gzip -d file.gz命令
	Windows用户	使用解压缩软件如WinRAR、7-Zip等
**file.zip形式的压缩文件**	Unix/Linux/Mac用户	使用unzip file.zip命令
	Windows用户	使用解压缩软件如WinRAR、7-Zip等

4.2 结果文件格式说明

文件类型	文件描述	打开方式
**file.fa/fasta**	序列文件，fasta格式，一般为基因序列或者基因组序列。因文件一般较大，打开较为困难	unix/Linux/Mac用户使用less或more命令
		windows用户使用高级文本编辑器Editplus/Notepad++等
**file.fq/fastq**	序列文件，fastq格式，一般为reads序列；因文件一般较大，打开较为困难	unix/Linux/Mac用户使用 less 或 more 命令
		windows用户使用高级文本编辑器Editplus/Notepad++等
**file.txt/xls**	结果数据表格文件；文件以制表符Tab分隔	unix/Linux/Mac用户使用less或more命令
		windows用户使用高级文本编辑器Editplus/Notepad++ 等，也可以用Microsoft Excel打开
**file.pdf/svg**	结果图像文件；矢量图，可以放大和缩小而不失真，方便用户查看和编辑处理，可使用Adobe Illustrator进行图片编辑，用于文章发表等	windows/Mac用户可以使用Adobe Reader/福昕阅读器/网页浏览器等打开
		unix/Linux用户使用evince命令打开
**file.png**	结果图像文件；位图，无损压缩	unix/Linux/Mac用户使用display命令打开
		windows用户可以使用图片浏览器打开，如photoshop等

4.3 分析软件列表及版本

分析	软件	版本
**比对分析**	hisat2	2.0.5
**定量分析**	featureCounts	1.5.0-p3
	stringtie	1.3.3b
**差异分析**	DESeq2	1.16.1
	edgeR	3.18.1
**富集分析**	clusterProfiler	3.4.4
**可变剪接**	rMATS	3.2.5
**变异分析**	GATK	3.7
	snpEff	4.3q
**融合基因**	star-fusion	1.2.0

4.4 methods

为了方便用户撰写文章，我们准备了数据分析所涉及到的中文版methods (src/methods_Chinese.pdf)和英文版methods (src/methods.pdf)

4.5 PCA分析说明

主成分分析，简写为PCA（Principle Component Analysis），是一种让数据降维的算法。PCA的思想是将维特征映射到维上（可以简单理解为投影），这个维特征称为主元，是重新构造出来的维特征（所以PC1不代表某个具体的特征）。PCA降维的算法涉及到很多线性代数的问题，非常复杂，在此不做展开。在转录组中，每个样本中都含有成千上万个基因，我们对这些基因计算了表达量值（FPKM），面对海量数据，我们很难从中看出来这些样品之间哪几个更为接近？谁更为特殊？假设样本中平均鉴定到3万个基因，那么也就意味着本数据有3万个维度，每个基因（维度）的定量信息的变化在样本中的分布都是不一样的，有些基因在样本中变异很小，有些很大，有些居中。应用PCA对整体数据进行降维，将3万个维度降至2-3个维度，从而简化了数据，突出了主要矛盾。

PCA是生物学重复好坏的另一种评价方式，图中点之间的距离即代表着样本之间的相似程度。PC1指排名第一的贡献率，是对变异量影响最大的一个因子，PC2就是排名第二的因子。因而我们往往更注重样本在PC1（横坐标）上的分布，如下图所示，TP_12h组的三个样本的PC1，PC2都非常接近，这样的样本实际上是很相似的。

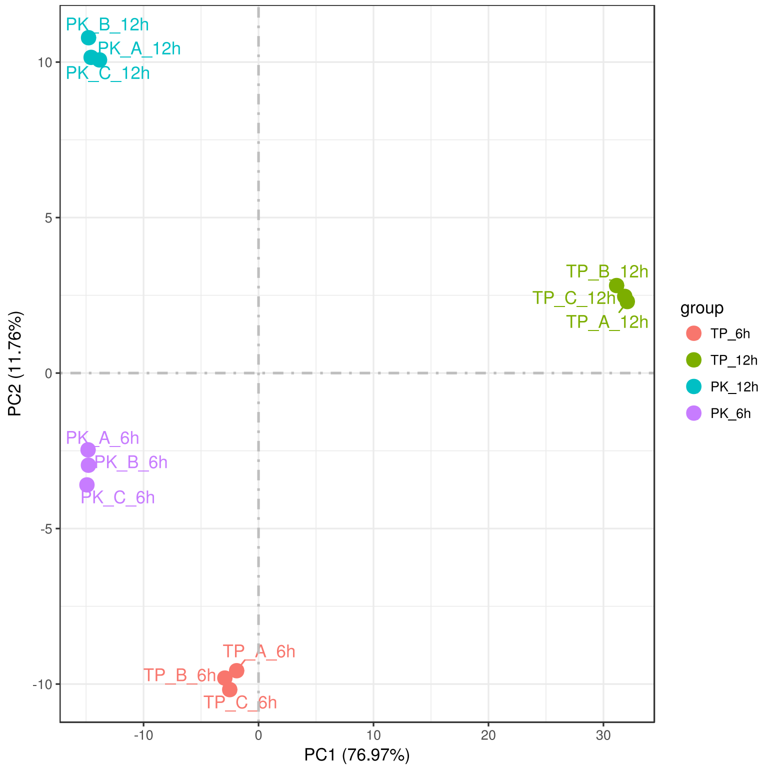

4.6 GO分析说明

GO(Gene Ontology，基因本体)是描述基因功能的综合性数据库，主要是利用基因的本质功能对基因进行分类，从而限定和描述基因和蛋白质的功能。GO数据库把基因的本体分为三种：生物过程（Biological Process, BP），细胞组成（Cellular Component, CC）和分子功能（Molecular Function, MF）。

BP本体论：分子功能的有序组合，达成更广的生物功能，如有丝分裂或嘌呤代谢等；

CC本体论：亚细胞结构、位置和大分子复合物，如核仁、端粒和识别起始的复合物等；

MF本体论：用于描述基因、基因产物的功能，如与碳水化合物结合或ATP水解酶活性等。

GO中最基本的概念是“标签（term）”，这些terms是用来描述基因和基因产物特性的，即GO数据库是给每个基因贴上标签，以便研究者能够通过标签快速寻找到目标基因。因为三种本体是可以交叉的，所以同一个基因注释到不同的类别中是很常见的，例如gene A定位于线粒体（CC）上，参与碳水化合物代谢（MF）。

4.7 KEGG分析说明

KEGG（Kyoto Encyclopedia of Genes and Genomes, 京都基因与基因组百科全书），是一个整合了基因组、化学和系统功能信息的数据库。

我们最常用的KEGG pathway数据库 (https://www.kegg.jp/kegg/pathway.html)存储了大量的功能信息，KEGG通路数据库中包含新陈代谢、遗传信息加工、环境信息加工、细胞过程、生物体系统、人类疾病和药物开发七种通路。一般，在KEGG中存在两种代谢图：

① reference pathway，根据已有的知识绘制的、概括的、详尽的具有一般参考意义的代谢图，为白色小框，在KEGG中名字以map开头，比如map00010；

② species-specific pathway，绿色小框为该物种特有的基因或酶，只有这些绿色的框有更详细的信息。KEGG中名字为特定物种种属英文缩写，比如酵母的糖酵解通路图，sce00010。

ko编号表示一个通路，这个通路是不分物种的，相当于所有物种的这一通路的并集。K编号表示一个基因，是ko通路中的基本单位，某一K编号代表的不是某一具体物种的基因，而是所有物种的某一同源基因的统称。

如何看懂通路图？参见官方说明 (https://www.genome.jp/kegg/document/help_pathway.html)

GO和KEGG Pathway富集分析结果的解读，应该从生物学意义的角度出发，P value和Q value只是个参考而已，那些不显著的通路也值得解读（从功能注释的角度解读，而不是从富集分析的角度解读）。只要结果可以解释，有意义，不用太执着。

4.8 Reactome分析说明

Reactome数据库 (https://reactome.org/)是一个免费的、开源的、手动整理的及经同行评审的pathway数据库。其目标是提供直观的生物信息学工具，包括对途径信息的可视化、解读及分析，以支持技术研究、基因组分析、建模、系统生物学和教育。

Reactome是反应（reaction）+组（ome）的合成词，涉及催化剂的结合、活化、易位、讲解和经典生物化学事件。Reactome数据模型的核心单元是反应（reaction），参与反应的实体（核酸、蛋白质、复合物、疫苗、抗癌治疗剂和小分子）形成生物相互作用的网络并呗分组成通路。Reactome中生物途径的实例包括经典的中间代谢、信号传导、转录调节、细胞凋亡和疾病。推断的直系同源反应可用于17种非人类物种，包括小鼠、大鼠、鸡、河豚、蠕虫、蝇、酵母、水稻和拟南芥。截止2018年12月13日，Reactome数据库包括人类pathway2256个，反应包括12788个，10792个蛋白，1827个小分子以及29454篇参考文献。引用了100多个不同的在线生物信息学资源库，包括NCBI、Ensembl、Uniport、UCSC、Pubmed数据库等。

4.9 DO分析说明

DO:疾病本体论 (http://www.disease-ontology.org/)是人类疾病的正式本体论。疾病本体论项目由马里兰大学医学院的基因组科学研究所主办。疾病本体项目最初于2003年在西北大学开发，旨在满足特定构建本体的需求，该本体论涵盖了在可扩展以满足社区需求的本体论框架内的生物医学资源库中注释的所有疾病概念。疾病本体论（DO）的使命是为与人类疾病相关的生物医学数据的整合提供开源本体论。

疾病本体标识符（DOID）由前缀DOID组成：后跟数字，例如，阿尔茨海默氏病标识符DOID：10652。

4.10 DisGeNET分析说明

DisGeNET数据库 (http://www.disgenet.org/)包含与人类疾病相关的最大公共基因和变体的信息。（Piñero等，2016;Piñero等，2015）。DisGeNET集成了专家策划存储库，GWAS目录，动物模型和科学文献中的数据。整合了多个数据库的gene-disease associations (GDAs)和大量的文献，并且采用文本挖掘技术对孟德尔疾病、复杂疾病和环境性疾病进行了相关性分析。具体技术包括对基因-疾病词汇的mapping、DisGeNET本体分析。

当前版本的DisGeNET（v6.0）包含628,685个基因-疾病关联（GDAs），17,549个基因和24,166个疾病，疾病，特征和临床或异常人类表型，以及210,498个变异疾病协会（VDA），介于117,337之间变异和10,358种疾病，特征和表型。

4.11 融合基因分析软件介绍

诺禾致源使用STAR-Fusion (https://github.com/STAR-Fusion/)软件进行融合基因的检测，STAR-Fusion是利用STAR比对的融合输出结果来检测融合转录本的软件包，STAR-Fusion分析流程如下图所示。分为SATR比对，STAR-Fusion.predict，STAR-Fusion.filter具体如下。

STAR比对：是STAR比对的标准比对过程中的一个延伸，第一步，利用MMP算法（连续最大可比对种子搜索）将种子准确的定位到参考基因组上，下一步，选择基因组比对窗口将固定的种子进行聚类。

STAR-Fusion.predict：将Split reads又称JunctionReads(含有两个基因融合断点的reads，流程图中S=3即为Split reads的数目)和Discordant pair又称SpanningFrags(不一致比对的reads，即reads的两端比对到不同的两个基因，图中J=2即为Discordant pair的数目）比对到参考基因组的注释文件。STAR-Fusion根据支持融合断点的Split reads和Discordant pair的数目应用最少reads支持准则对融合基因进行筛选。

STAR-Fusion.filter：这一步是融合基因检测的最后一步，主要是筛选出最可靠的融合基因，过滤到最初预测的不可靠的候选的融合基因。过滤的过程主要是先按照基因对和断点的距离进行分类，然后按照在推断的断点reads的支持和比对的范围以及推断的融合基因的序列的相似性，过滤掉配对混乱的融合基因对。

FusionInspector 是一个癌症转录分析工具包，软件会对STAR-Fusion的预测结果通过再检测，再次打分，进一步对融合基因的分析结果进行校正分析。具体方法：输入的文件是我们用STAR-Fusion得到的融合基因的列表，参考基因组文件，样品的clean data。FusionInspector会根据STAR-Fusion得到的融合基因，对每对融合基因提取在基因组上的区域并构建融合基因的最小融合的contig序列。将reads比对到这些候选的融合的contig上。最后对每个融合基因支持的JunctionReads与SpanningFrags进行识别。得到最终的融合基因列表。

STAR-Fusion官网推荐对STAR-Fusion的预测结果再次用校验工具FusionInspector进行进一步的矫正分析。通过两次的矫正保证了融合基因结果的准确性。我们融合基因分析的过程就是利用FusionInspector对STAR-Fusion预测结果进行校正。

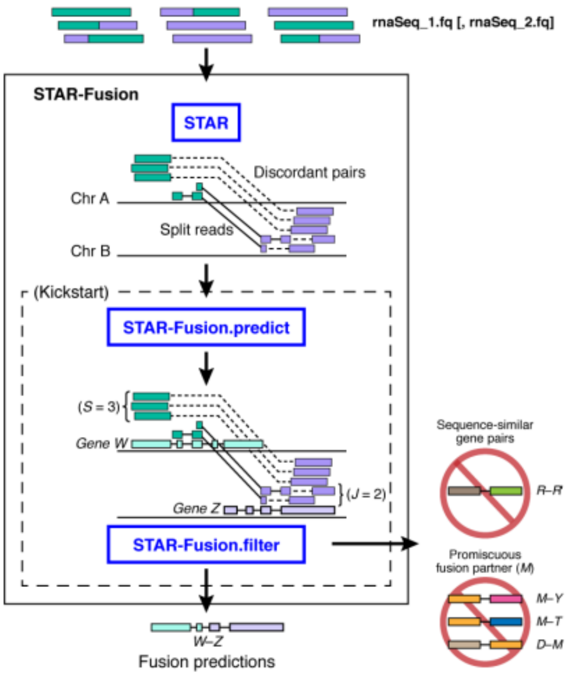

4.12 GSEA富集分析

Gene Set Enrichment Analysis(GSEA)不需要指定明确的差异基因阈值，把基因按照在两组样本中的差异表达程度进行排序，然后采用统计学方法检验预先设定的基因集合是否在排表的顶端或低段富集。更容 易囊括细微但协调性的变化对生物通路的影响。

GSEA分析原理：主要包括三个步骤:计算富集得分(Enrichment Score);估计富集得分的显著性水平;多重假设检验。

计算富集得分 (ES, enrichment score)。ES反应基因集成员s在排序列表L的两端富集的程度。计算方式是，从基因集L的第一个基因开始，计算一个累计统计值。当遇到一个落在s里面的基因，则增加统计值。遇到一个不在s里面的基因，则降低统计值。每一步统计值增加或减少的幅度与基因的表达变化程度(更严格的是与基因和表型的关联度)是相关的。富集得分ES最后定义为最大的峰值。正值ES表示基因集在列表的顶部富集，负值ES表示基因集在列表的底部富集。

评估富集得分(ES)的显著性(Normalized Enrichment Score)，NES通过基于表型而不改变基因之间关系的排列检验(permutation test)计算观察到的富集得分(ES)出现的可能性。GSEA考虑了gene set大小、gene set与基因表达水平的相关性的差异;因此，NES可用于比较不同gene set的分析结果多重假设检验矫正

多重假设检验。首先对每个基因子集s计算得到的ES根据基因集的大小进行标准化得到Normalized Enrichment Score (NES)。随后针对NES计算假阳性率。Leading-edge subset对富集得分贡献最大的基因成员。

4.13 数据挖掘和验证

转录组分析的内容中包含了数据质控、基因结构分析和基因表达水平分析三部分，其中的核心是基因表达水平分析。转录组数据的挖掘基本上遵循从整体到局部的方式，可以从表达量，功能和特定基因进行分析。

4.13.1 整体调控变化

（1）整体差异基因分析

首先我们一定要明确研究目的，为此采用了什么样的实验方案设计。在做差异分析之前，一定要根据我们的研究目的来确定比较组，差异分析的结果一定要结合实验设计。一般来说我们的实验设计都是根据表型来的，我们通过转录组数据得到的基因水平的变化，反应的就是表型的差异。差异表达基因（DEG）筛选是转录组测序分析的核心基础，往往那些表达量高且差异很大的基因，或者是共有的差异基因，就是我们可以重点关注的基因。

（2）基因表达模式分析

参与同一生物过程的基因通常受同一个调控系统的控制，即参与同一生物过程中的基因有着相似或相同的变化规律。因此，通过基因在不同样品中的表达模式的分析，获得表达模式相近的基因模块，进而联系表型筛选关键基因模块。常用的基因表达模式分析方法有聚类热图，趋势分析和WGCNA，聚类热图分析适用于已经初步锁定某些基因之后的分析，趋势分析需要具有至少3个以上的连续变量的实验设计，WGCNA则适合大样本量的实验设计。无论是哪一种方式，都是以找到与性状最相关的那个表达模块的基因为目标的，应该重点关注与性状变化一致或相反的表达模式的模块。

（3）差异基因功能富集

通过基因的表达量找到的基因，最终也要回归到功能上面来，富集分析就是通过不同的数据库对基因的功能进行归类。富集分析的思路，就是研究差异的基因显著富集在哪些通路之中。富集分析结果中那些显著富集的通路肯定是具有重要作用的关键通路，可以直接对这些通路展开讨论和分析。此外，我们可以根据研究目的，直接找到我们想要的通路，比如当我们研究植物的生长发育的时候，Plant Hormone Signal transduction pathway必然是我们需要重点去关注的。

（4）特定基因分析

我们通过上面的思路找到一些可能起到作用的基因，这些基因我们称为潜在的关键基因，下一步就是针对这些基因进行进一步分析，锁定目标基因。我们可以对候选的基因进行表达水平分析和功能分析，关键基因一定具有高表达量，且差异显著，还要具有和我们的研究目的相符的功能。一个常用的快速锁定目标基因的思路是寻找 “明星”分子，这个需要做文献收集的工作，从前人的研究中总结出来某方面的明星基因，再到我们的项目中重点看这些基因的表达和结构等信息。假设我们的项目是研究抗癌的，那么肯定得关注P53等抗癌基因。另一方面，除了基因层面，我们也可以关注Pathway层面，例如Pathways in cancer等和癌症相关的信号通路。还有一种方法是我们可以自己造星，如果我们寻找到的一些关键基因并非明星基因，我们可以想方设法往研究热点上去靠，让这些新的基因成为明星，比如热度一直不减的转录因子（TF）研究。

4.13.2 实验验证

（1）基因表达水平验证

qRT-PCR验证：验证基因表达水平，基于相较于内参基因（管家基因：维持细胞基因代谢活动所必须的基因，在各组织和细胞中表达相对稳定）表达水平，获得目标基因的相对定量水平，常见的内参基因GAPDH、β-Actin、18S rRNA等。注：因qRT-PCR与转录组定量原理不同，会有表达量差异，一般建议看基因表达趋势是否一致。

Western Blot：验证差异基因是否在翻译水平发生变化，应用特异性抗体，标记蛋白水平变化。常见的抗体类型包括兔抗、羊抗、鼠抗等。**注：文献调研蛋白与转录水平相关性较弱，因此建议老师研究功能层面，需蛋白层面的验证。**

Northern Blot：验证RNA的表达情况，且对目的片段大小、是否发生可变剪切等有较高的灵敏度：Northern Blot采用琼脂糖凝胶电泳，根据分子量大小不同将不同的RNA分离开来，将其原位转移至固相支持物（如尼龙膜、纤维膜等）上，再用标记过的DNA或RNA探针，依据碱基互补配对的原则进行杂交，后进行显影或显色，以检测目标RNA所在位置，而其显影强度则可指示目标RNA在所测样品中的相对含量。

（2）基因定位验证

原位杂交实验：利用RNA探针杂交技术，通过显影技术检测目标基因在细胞内的位置。

亚细胞定位：通过转染重组表达质粒载体，通过荧光显微电镜检测目标基因在细胞中表达位置，进而分析其发挥的功能。

（3）基因功能验证

基因过表达/敲除、沉默（RNAi）：构建过表达目标基因载体质粒/siRNA，转染细胞后，检测细胞形态、生长、增殖、迁移速度等指标，挖掘目标基因功能。

基因编辑：构建CRISPER-Cas 9系统，定向编辑目标基因，检测靶向基因表达、细胞形态等生理指标。

荧光素酶双报告系统：目标基因存在靶向调控RNA时，可通过构建双报告质粒载体，共转染至细胞中，通过检测荧光信号与对照组之间的差距，分析靶向作用机制。

为保证售后处理的及时性和专业性，我们特成立专门的售后组解决您的问题，如您在报告解读和数据挖掘处理中有任何疑问和需要协助解决的均可联系我们的售后组同事，售后受理邮箱：service-rna@novogene.com (mailto:service-rna@novogene.com)，电话：400-658-1585

4.14 参考文献

- [1] Wang Z, Gerstein M, Snyder M. RNA-Seq: a revolutionary tool for transcriptomics[J]. Nature Reviews Genetics, 2009, 10(1): 57-63.
- [2] Parkhomchuk D, Borodina T, Amstislavskiy V, et al. Transcriptome analysis by strand-specific sequencing of complementary DNA[J]. Nucleic acids research, 2009, 37(18): e123-e123.
- [3] Goldstein L D , Cao Y , Pau G , et al. Prediction and Quantification of Splice Events from RNA-Seq Data.[J]. Plos One, 2016, 11(5):e0156132.
- [4] Mortazavi A, Williams B A, McCue K, et al. Mapping and quantifying mammalian transcriptomes by RNA-Seq[J]. Nature methods, 2008, 5(7): 621-628.
- [5] Mihaela Pertea, Geo M Pertea, Corina M Antonescu1,et al.StringTie enables improved reconstruction of a transcriptome from RNA-seq reads[J].Nat Biotechnol. 2015 March ; 33(3):290-295(StringTie)
- [6] Liao Y1, Smyth GK, Shi W. featureCounts: an efficient general purpose program for assigning sequence reads to genomicfeatures.Bioinformatics.2014 ,30(7):923-30.(featureCounts)
- [7] Garber M, Grabherr M G, Guttman M, et al. Computational methods for transcriptome annotation and quantification using RNA-seq[J]. Nature methods, 2011, 8(6): 469-477.
- [8] Bray N, Pimentel H, Melsted P, et al. Near-optimal RNA-Seq quantification[J]. arXiv preprint arXiv:1505.02710, 2015.
- [9] Patro R, Mount S M, Kingsford C. Sailfish enables alignment-free isoform quantification from RNA-seq reads using lightweight algorithms[J]. Nature biotechnology, 2014, 32(5): 462-464.
- [10] Anders S, Huber W. Differential expression analysis for sequence count data[J]. Genome biol, 2010, 11(10): R106.
- [11] Love M I, Huber W, Anders S. Moderated estimation of fold change and dispersion for RNA-seq data with DESeq2[J]. Genome biology, 2014, 15(12): 1-21.(DESeq2)
- [12] Robinson M D, McCarthy D J, Smyth G K. edgeR: a Bioconductor package for differential expression analysis of digital gene expression data[J]. Bioinformatics, 2010, 26(1): 139-140.(edgeR)
- [13] Young M D, Wakefield M J, Smyth G K, et al. Method Gene ontology analysis for RNA-seq: accounting for selection bias[J]. Genome Biol, 2010, 11: R14.
- [14] He Z , Zhao X , Lu Z , et al. Comparative transcriptome and gene co-expression network analysis reveal genes and signaling pathways adaptively responsive to varied adverse stresses in the insect fungal pathogen, Beauveria bassiana[J]. Journal of Invertebrate Pathology, 2017:S0022201117304391.
- [15] Kanehisa M, Goto S. KEGG: kyoto encyclopedia of genes and genomes[J]. Nucleic acids research, 2000, 28(1): 27-30.(KEGG)
- [16] Shen S., Park JW., Lu ZX., Lin L., Henry MD., Wu YN., Zhou Q., Xing Y. rMATS: Robust and Flexible Detection of Differential Alternative Splicing from Replicate RNA-Seq Data.(rMATS).
- [17] Katz Y, Wang E T, Airoldi E M, et al. Analysis and design of RNA sequencing experiments for identifying isoform regulation[J]. Nature methods, 2010, 7(12): 1009-1015.
- [18] McKenna A, Hanna M, Banks E, et al. The Genome Analysis Toolkit: a MapReduce framework for analyzing next-generation DNA sequencing data[J]. Genome research, 2010, 20(9): 1297-1303.(GATK)
- [19] View ORCID ProfileBrian Haas, Alexander Dobin, Nicolas Stransky,et al. STAR-Fusion: Fast and Accurate Fusion Transcript Detection from RNA-Seq[J]. biorxiv, 2017.(STAR-Fusion)
